# The global burden of pediatric infective endocarditis (5–14 years): epidemiological patterns from 1990 to 2021 and projected trajectories

**DOI:** 10.3389/fcvm.2025.1657644

**Published:** 2025-10-20

**Authors:** Ying Xiao, Yin Li, YuLing Hou, Zugen Cheng, TingTing Sun, Yuan Liao, Yan Li

**Affiliations:** ^1^ Department of Cardiology, Kunming Children's Hospital, Kunming City, Yunnan, China; ^2^ Clinical Nutrition Department, Kunming Children’s Hospital, Kunming City, Yunnan, China; ^3^ Human Resources Department, Kunming Children’s Hospital, Kunming City, Yunnan, China

**Keywords:** infective endocarditis (IE), children, incidence, mortality, DALYs—disability-adjusted life years, ARIMA (auto regressive integrated moving average)

## Abstract

**Background:**

Pediatric infective endocarditis (IE) remains a rare but life-threatening condition, with substantial morbidity and mortality among children worldwide. Despite advances in cardiac care and infection control, the global burden, temporal trends, and regional disparities of pediatric IE remain poorly characterized.

**Methods:**

We conducted a comprehensive analysis of pediatric IE burden among children aged 5–14 years using the Global Burden of Disease (GBD) 2021 dataset. Annual incidence, mortality, and disability-adjusted life years (DALYs) attributable to IE were estimated across 204 countries and territories from 1990 to 2021. Analyses were stratified by sex, region, and socio-demographic index (SDI). An autoregressive integrated moving average (ARIMA) model was applied to forecast pediatric IE trends through 2035.

**Results:**

Globally, the incidence of pediatric IE declined by 27.6% (95% UI, −30.9% to −24.9%) and mortality by 21.4% (95% UI, −35.2% to −0.8%) between 1990 and 2021, with an estimated annual percentage change (EAPC) of −1.37% (95% CI, −1.49% to −1.24%) and −0.64% (95% CI, −0.75% to −0.54%), respectively. DALYs decreased by 19.2% (95% UI, −33.2% to 1.9%). Marked disparities persisted, with high-SDI regions, notably China, achieving the greatest reductions in burden, while sub-Saharan Africa and Eastern Europe exhibited stagnant or increasing rates. The ARIMA model projected a potential stabilization or slight rebound in the global pediatric IE burden by 2035, particularly among girls, although wide confidence intervals highlight the uncertainty of long-term forecasts.

**Conclusions:**

While significant progress has been made in reducing the global burden of pediatric IE, major geographic and socio-demographic inequalities persist. Sustained efforts to strengthen early detection, risk stratification, prevention (including rheumatic heart disease control and antibiotic prophylaxis), and equitable access to cardiac care are urgently required—especially in low- and middle-income countries. Enhanced surveillance and data collection will be crucial to monitor trends, evaluate interventions, and achieve further reductions in pediatric IE morbidity and mortality.

## Introduction

Pediatric infective endocarditis (IE) remains a rare yet severe cardiovascular infection in children, with an annual incidence below 1 case per 100,000 (1). Despite advances in treatment, contemporary studies report mortality rates ranging from 15% to 25%, alongside significant morbidity (2, 3). Clinical complications such as heart failure, embolic strokes, and organ damage are frequent in those who survive (4, 5). Once predominantly linked to rheumatic heart disease (RHD) in pediatric patients (6), the epidemiology of IE in children has evolved. Improved survival of infants with congenital heart disease (CHD) and the widespread use of invasive medical interventions have shifted pediatric IE toward a disease of iatrogenic and congenital risk factors (7). CHD has become the leading predisposing condition for pediatric IE in high-income countries (8), with 50%–70% of cases occurring in children with structural heart defects (9). Many patients are those who have undergone cardiac surgery or have indwelling intravascular devices, reflecting how advances in congenital cardiac care have inadvertently expanded the population at risk. At the same time, IE continues to affect both healthy children and those with preexisting conditions, while persisting as a complication of RHD in resource-limited settings (10). Pediatric IE, therefore, represents a small fraction of overall IE cases, but it is a frequently catastrophic disease with unique epidemiologic drivers and persistently poor outcomes.

Despite its severity, pediatric IE has been relatively neglected as a subject of global epidemiologic study. Our understanding of pediatric IE burden and trends is limited by the rarity of the disease and the paucity of comprehensive data, especially from low- and middle-income countries (LMICs). Most available literature consists of single-center case series or extrapolations from adult IE studies (11–13), which inherently constrain longitudinal and age-specific insights.

Here we present the first comprehensive global analysis of pediatric IE, addressing this evidence gap through a systematic examination of the Global Burden of Disease (GBD) 2021 data. The GBD study provides standardized estimates of disease incidence, mortality, and disability-adjusted life years (DALYs) across 204 countries and territories, over decades and by age and sex (14). Leveraging this robust dataset, we quantified the national, regional, and global burden of pediatric IE in children aged 5–14 years from 1990 to 2021, including annual case incidence, deaths, and DALYs attributable to IE. In doing so, we identified historical trends and geographic patterns in pediatric IE that have not been previously characterized on a global scale. Moreover, to anticipate future needs, we applied an autoregressive integrated moving average (ARIMA) time-series model to project pediatric IE burden through the year 2035.

In sum, quantifying the global burden of pediatric IE and its future trajectory is more than an academic exercise, it is a necessary step toward raising awareness of this rare but deadly disease, allocating resources appropriately, and ultimately improving cardiovascular outcomes for children worldwide.

## Methods

### Overview and methodological details

The GBD, Injuries, and Risk Factors Study 2021 analyzed 370 diseases and injuries across 204 countries and territories, reporting incidence, mortality, and DALYs (15). DALYs combine years of life lost (YLL) from premature death and years lived with disability (YLD) (16, 17), calculated as:

(1) YLL=Numberofdeaths×Standardlifeexpectancyatageofdeath

(2) YLD=prevalence×Disabilityweight

Data were extracted from authoritative public databases following rigorous quality control protocols (18). The GBD Collaborative Network annually updates these datasets, which undergo standardized cleaning, transformation, and modeling by participating institutions (https://www.healthdata.org/data-tools-practices/data-collection).

This study examined pediatric IEs cases, incidence, mortality, and DALYs among children aged 5–14 years from 1990 to 2021 using GBD data downloaded on December 3, 2024. Analyses stratified by sex, age, and geographical region were performed, though ethnicity and race could not be assessed due to data limitations. The methodology complied with the Strengthening the Reporting of Observational Studies in Epidemiology (STROBE) guidelines for observational studies (19).

### Sociodemographic index

The Socio-demographic Index (SDI) quantifies regional or national socioeconomic development through a composite measure integrating economic indicators, education levels, living conditions, and social welfare systems (20). Its standardized scale (0–1) reflects developmental status, where elevated values denote greater advancement. The GBD stratifies populations into five SDI tiers—low, low-middle, middle, high-middle, and high—enabling systematic analysis of socioeconomic and geographic influences on childhood IE epidemiology.

### Role of the funding source

The study funders were not involved in designing the research, collecting or analyzing data, interpreting results, or preparing the manuscript. All authors accessed the complete dataset and assume full responsibility for the publication decision.

### Statistical analysis

Based on the GBD database, the incidence rate, mortality rate, and DALY rate per 100,000 population were calculated along with their 95% uncertainty intervals (UIs). The Joinpoint regression model was employed to determine the annual percentage change (APC) and its 95% confidence interval (CI), enabling evaluation of temporal trends within each independent period (21). This approach provides a detailed understanding of annual rate fluctuations, offering granular insights into year-to-year variations.

A log-transformed linear regression model was utilized to compute the estimated average annual percentage change (EAPC) and its CI, analyzing temporal trends in the incidence, mortality, and DALYs of pediatric IE from 1990 to 2021 (22). The EAPC is particularly valuable for examining long-term trends, as it untangles whether occurrence rates generally increase or decrease over time, irrespective of short-term fluctuations. An EAPC value with its lower 95% CI bound >0 indicates an upward trend for the corresponding metric, whereas an EAPC value with its upper 95% CI bound <0 signifies a downward trend.

The relationship between disease burden indicators and the SDI was analyzed using fitted curves. Autoregressive Integrated Moving Average (ARIMA) modeling was applied to project disease burden estimates for 2035 (23). All analyses were conducted using R software (version 4.4.2), with statistical significance set at *p* < 0.05.

## Results

### Global trends

#### Incidence

**Figure 1:**
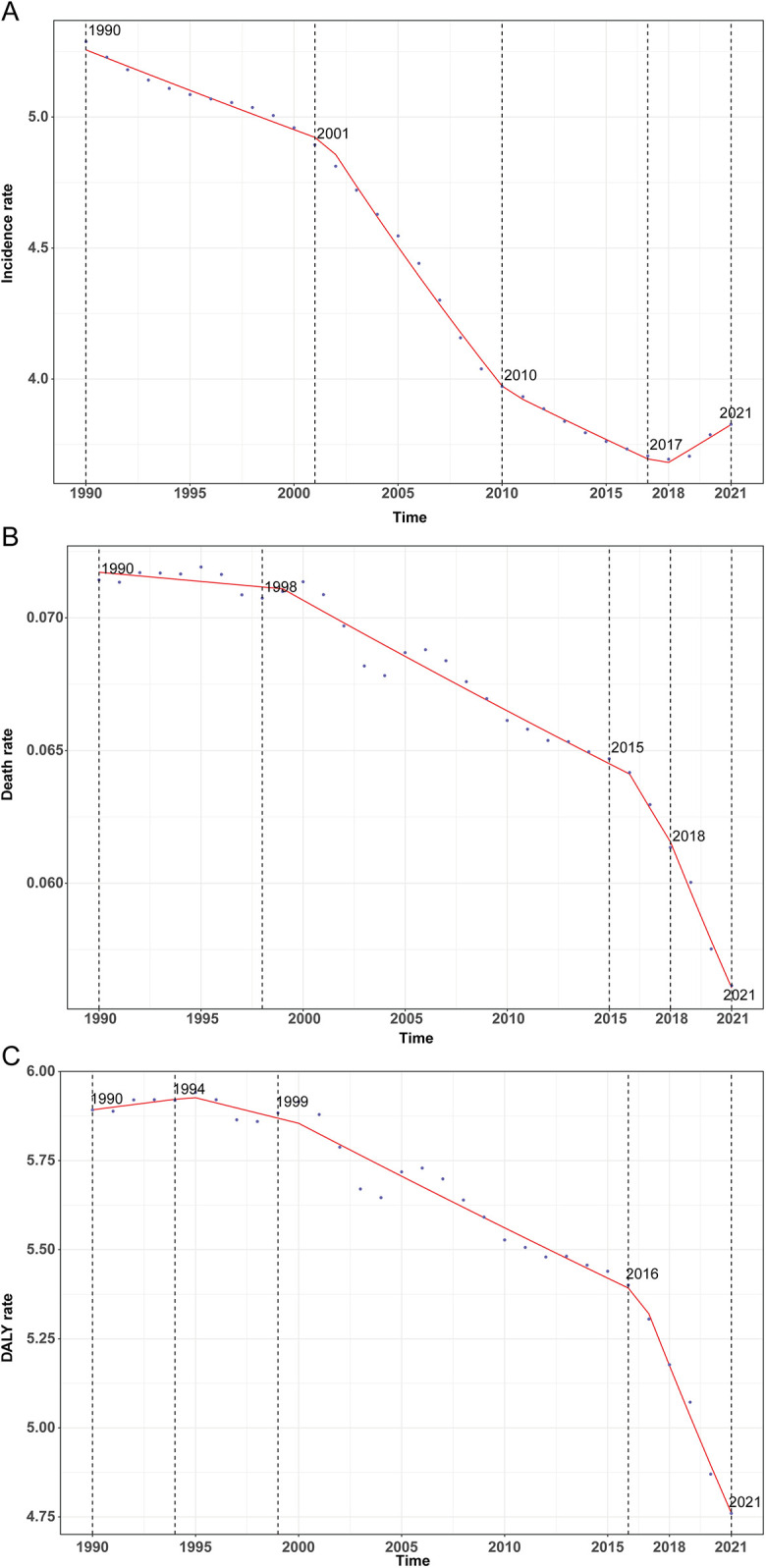
Annual percent change (APC) and trends in global childhood infective endocarditis incidence, mortality, and disability-adjusted life years (DALYs) from 1990 to 2021. **(A)** Incidence rate. **(B)** Mortality rate. **(C)** DALY rate.

Between 1990 and 2021, the global incidence and number of IE cases among children aged 5–14 years underwent substantial changes. The estimated number of new cases decreased from 59,183 (95% UI, 38,499–88,373) in 1990 to 51,807 (95% UI, 33,497–77,897) in 2021, representing a 12.46% reduction (95% UI, −16.36% to −9.11%). Correspondingly, the global incidence rate declined from 5.29 per 100,000 (95% UI, 3.44–7.90) in 1990 to 3.83 per 100,000 (95% UI, 2.47–5.75) in 2021, a relative decrease of 27.63% (95% UI, −30.85% to −24.85%) with an EAPC of −1.37% (95% CI, −1.49% to −1.24%) (Table 1). Notably, the incidence rate initially decreased, reaching its lowest annual percent change (APC) of −2.48% (95% CI, −2.61% to −2.35%) during 2001–2010. The lowest incidence rate was recorded in 2018 at 3.69 per 100,000 (95% UI, 2.39–5.56; Figure 1A).

**Table 1:** Incidence of infective endocarditis in children between 1990 and 2021 at the global and regional level.

location	1990		2021		1990–2021		
	Incident cases	Incidence rate	Incident cases	Incidence rate	Cases change^b^	Rate change^b^	EAPC^a^
Global	59183.53 (38498.56, 88373.42)	5.29 (3.44, 7.90)	51807.05 (33497.37, 77896.75)	3.83 (2.47, 5.75)	−12.46 (−16.36, −9.11)	−27.63 (−30.85, −24.85)	−1.37 (−1.49, −1.24)
High SDI	6549.43 (4187.80, 10128.35)	5.28 (3.37, 8.16)	5348.87 (3548.11, 8007.81)	4.51 (2.99, 6.75)	−18.33 (−23.73, −11.74)	−14.61 (−20.26, −7.73)	−1.41 (−1.85, −0.97)
High-middle SDI	13293.76 (8877.60, 20141.98)	7.36 (4.91, 11.15)	6844.06 (4410.22, 10,474.11)	4.26 (2.74, 6.51)	−48.52 (−52.27, −45.00)	−42.16 (−46.37, −38.21)	−2.28 (−2.57, −2.00)
Middle SDI	24909.08 (16576.18, 37114.13)	6.61 (4.40, 9.85)	15061.83 (9613.15, 22914.02)	3.86 (2.46, .87)	−39.53 (−43.42, −36.09)	−41.63 (−45.39, −38.31)	−2.12 (−2.29, −1.94)
Low-middle SDI	8271.83 (5233.85, 12761.10)	2.77 (1.75, 4.27)	11753.14 (7455.45, 17755.53)	3.03 (1.92, 4.57)	42.09 (37.63, 47.20)	9.28 (5.86, 13.22)	0.29 (0.21, 0.37)
Low SDI	6119.73 (4137.21, 8912.75)	4.43 (3.00, 6.45)	12757.13 (8595.43, 18599.36)	4.33 (2.92, 6.31)	108.46 (102.41, 114.35)	−2.28 (−5.12, 0.48)	−0.07 (−0.17, 0.03)
Regions							
Andean Latin America	334.25 (230.11, 485.30)	3.49 (2.40, 5.07)	449.47 (296.48, 659.44)	3.76 (2.48, 5.52)	34.47 (26.10, 42.26)	7.79 (1.08, 14.04)	0.28 (0.24, 0.31)
Australasia	84.72 (49.84, 136.01)	2.78 (1.64, 4.47)	111.33 (66.44, 177.77)	2.84 (1.70, 4.54)	31.41 (22.99, 40.98)	2.16 (−4.39, 9.60)	0.13 (0.05, 0.20)
Caribbean	420.63 (286.44, 610.11)	5.78 (3.93, 8.38)	426.48 (283.04, 624.79)	5.58 (3.71, 8.18)	1.39 (−3.89, 6.42)	−3.33 (−8.37, 1.46)	−0.17 (−0.26, −0.09)
Central Asia	270.60 (153.83, 446.34)	1.75 (0.99, 2.89)	350.05 (207.21, 561.53)	1.98 (1.17, 3.18)	29.36 (20.27, 37.21)	13.18 (5.22, 20.04)	0.50 (0.44, 0.56)
Central Europe	552.40 (335.28, 883.83)	2.71 (1.65, 4.34)	351.92 (215.17, 553.05)	2.90 (1.78, 4.56)	−36.29 (−38.50, −33.54)	7.02 (3.30, 11.63)	0.15 (0.11, 0.18)
Central Latin America	1589.40 (994.94, 2447.71)	3.84 (2.41, 5.92)	1820.81 (1133.68, 2785.53)	4.20 (2.61, 6.42)	14.56 (9.01, 20.56)	9.20 (3.91, 14.92)	0.26 (0.13, 0.38)
Central Sub-Saharan Africa	649.15 (417.07, 978.45)	4.35 (2.80, 6.56)	1704.26 (1120.47, 2546.28)	4.53 (2.98, 6.77)	162.54 (147.98, 181.01)	4.09 (−1.68, 11.42)	0.20 (0.14, 0.25)
East Asia	26083.75 (17559.69, 39056.92)	12.18 (8.20, 18.24)	8701.24 (5641.94, 13203.98)	4.65 (3.01, 7.05)	−66.64 (−70.24, −63.27)	−61.87 (−65.98, −58.01)	−3.79 (−4.22, −3.36)
Eastern Europe	1031.71 (618.20, 1640.70)	3.01 (1.81, 4.79)	972.64 (600.42, 1500.23)	3.84 (2.37, 5.92)	−5.73 (−11.77, 1.21)	27.39 (19.22, 36.76)	0.89 (0.80, 0.97)
Eastern Sub-Saharan Africa	2602.85 (1672.21, 3953.18)	4.78 (3.07, 7.26)	5319.59 (3436.57, 8007.70)	4.64 (3.00, 6.99)	104.38 (98.72, 112.43)	−2.86 (−5.55, 0.97)	−0.09 (−0.20, 0.02)
High-income Asia Pacific	1148.86 (722.61, 1778.76)	4.60 (2.89, 7.12)	570.09 (348.84, 899.28)	3.57 (2.18, 5.63)	−50.38 (−54.29, −47.66)	−22.39 (−28.51, −18.14)	−1.08 (−1.26, −0.90)
High-income North America	2708.42 (1710.38, 4226.25)	6.77 (4.28, 10.57)	2420.50 (1665.39, 3576.15)	5.36 (3.69, 7.93)	−10.63 (−20.83, 2.88)	−20.78 (−29.83, −8.80)	−2.75 (−3.72, −1.78)
North Africa and Middle East	3741.43 (2380.67, 5733.70)	4.19 (2.67, 6.42)	5035.18 (3157.39, 7727.21)	4.12 (2.58, 6.32)	34.58 (29.93, 39.05)	−1.69 (−5.09, 1.58)	−0.03 (−0.09, 0.03)
Oceania	67.64 (42.58, 104.84)	4.04 (2.54, 6.26)	119.52 (74.93, 180.79)	3.80 (2.38, 5.75)	76.70 (65.73, 88.61)	−5.90 (−11.74, 0.45)	−0.18 (−0.27, −0.08)
South Asia	4213.14 (2309.91, 7001.73)	1.52 (0.84, 2.53)	5583.95 (3102.72, 9034.07)	1.60 (0.89, 2.59)	32.54 (26.44, 40.39)	5.11 (0.28, 11.35)	0.20 (0.11, 0.29)
Southeast Asia	4555.76 (2861.25, 7054.09)	4.05 (2.54, 6.27)	4306.11 (2686.44, 6622.76)	3.70 (2.31, 5.69)	−5.48 (−8.71, −1.39)	−8.66 (−11.78, −4.70)	−0.27 (−0.39, −0.15)
Southern Latin America	326.60 (207.07, 510.71)	3.34 (2.12, 5.22)	493.42 (316.14, 754.67)	4.83 (3.09, 7.39)	51.08 (36.72, 70.64)	44.61 (30.86, 63.33)	1.27 (1.16, 1.39)
Southern Sub-Saharan Africa	610.04 (374.18, 963.13)	4.62 (2.83, 7.29)	638.38 (394.74, 998.04)	3.98 (2.46, 6.22)	4.64 (0.77, 9.32)	−13.76 (−16.95, −9.91)	−0.51 (−0.66, −0.36)
Tropical Latin America	1753.80 (1100.54, 2680.71)	4.80 (3.01, 7.34)	1517.52 (973.59, 2289.35)	4.60 (2.95, 6.94)	−13.47 (−17.10, −9.12)	−4.16 (−8.18, 0.66)	−0.14 (−0.30, 0.03)
Western Europe	1930.55 (1243.01, 3008.83)	4.02 (2.59, 6.26)	1803.70 (1189.58, 2679.79)	3.85 (2.54, 5.72)	−6.57 (−12.37, 0.58)	−4.23 (−10.18, 3.10)	−0.17 (−0.35, 0.01)
Western Sub-Saharan Africa	4507.83 (3348.10, 6140.99)	8.65 (6.42, 11.78)	9110.87 (6562.98, 12592.76)	6.76 (4.87, 9.34)	102.11 (89.70, 112.71)	−21.83 (−26.63, −17.74)	−0.85 (−1.00, −0.71)

EAPC, estimated annual percentage change; SDI, sociodemographic index; UI, uncertainty interval.

^a^ EAPC is expressed as 95% confidence interval.

^b^ Change shows the percentage change.

#### Mortality

Globally, annual deaths attributable to IE among children declined modestly from 799 (95% UI, 514–1,008) in 1990 to 760 (95% UI, 551–903) in 2021, reflecting a 4.93% decrease (95% UI, −21.63% to 19.95%). The mortality rate declined more sharply, from 0.07 per 100,000 (95% UI, 0.05–0.09) to 0.06 per 100,000 (95% UI, 0.04–0.07), a reduction of 21.39% (95% UI, −35.20% to −0.82%), with an EAPC of −0.64% (95% CI, −0.75% to −0.54%) (Table 2). Mortality rates exhibited a continuous downward trajectory, reaching the lowest observed APC of −3.07% (95% CI, −4.07% to −2.06%) during 2018–2021, and the lowest absolute mortality rate in 2021 (Figure 1B).

**Table 2:** Mortality of infective endocarditis in children Between 1990 and 2021 at the global and regional level.

location	1990 (95% UI)		2021 (95% UI)		1990–2021		
	Death cases	Death rate	Death cases	Death rate	Cases change^b^	Rate change^b^	EAPC^a^
Global	799.42 (514.13, 1007.54)	0.07 (0.05,0.09)	760.04 (551.02,902.68)	0.06 (0.04,0.07)	−4.93 (−21.63,19.95)	−21.39 (−35.20,−0.82)	−0.64 (−0.75,−0.54)
High SDI	29.41 (26.22,32.96)	0.02 (0.02,0.03)	20.35 (18.92,21.77)	0.02 (0.02,0.02)	−30.79 (−40.14,−20.97)	−27.64 (−37.42,−17.37)	−0.98 (−1.19,−0.76)
High-middle SDI	98.52 (66.12,126.75)	0.05 (0.04,0.07)	39.41 (33.65,50.39)	0.02 (0.02,0.03)	−60.00(−72.40,−40.38)	−55.05(−68.99,−33.01)	−2.57(−2.74,−2.40)
Middle SDI	309.18 (207.17,376.27)	0.08 (0.05,0.10)	185.58 (155.03,224.17)	0.05 (0.04,0.06)	−39.97 (−52.98,−18.74)	−42.06 (−54.61,−21.56)	−1.51 (−1.66,−1.35)
Low-middle SDI	199.83 (127.69,261.86)	0.07 (0.04,0.09)	214.42 (158.07,259.69)	0.06 (0.04,0.07)	7.30 (−14.54,38.70)	−17.47 (−34.27,6.68)	−0.48 (−0.62,−0.34)
Low SDI	161.72 (79.53,228.02)	0.12 (0.06,0.17)	299.30 (169.96,415.63)	0.10 (0.06,0.14)	85.07 (40.38,166.39)	−13.25 (−34.20,24.87)	−0.53 (−0.61,−0.46)
Regions							
Andean Latin America	11.93 (8.01,15.95)	0.12 (0.08,0.17)	10.39 (8.03,13.61)	0.09 (0.07,0.11)	−12.95 (−40.61,25.36)	−30.22 (−52.39,0.49)	−0.92 (−1.15,−0.70)
Australasia	0.32 (0.28,0.37)	0.01 (0.01,0.01)	0.44 (0.37,0.53)	0.01 (0.01,0.01)	37.55 (6.92,76.06)	6.93 (−16.87,36.87)	0.10 (−0.27,0.47)
Caribbean	7.97 (6.01,10.91)	0.11 (0.08,0.15)	10.10 (6.42,14.40)	0.13 (0.08,0.19)	26.71(−12.36,85.34)	20.81(−16.44,76.70)	0.74 (0.67,0.81)
Central Asia	3.16 (2.55,4.09)	0.02 (0.02,0.03)	2.27 (1.93,2.64)	0.01 (0.01,0.01)	−28.03 (−46.07,−5.01)	−37.03 (−52.82,−16.89)	−1.59 (−2.00,−1.19)
Central Europe	3.80 (3.48,4.29)	0.02 (0.02,0.02)	1.72 (1.52,1.88)	0.01 (0.01,0.02)	−54.81 (−60.19,−48.89)	−24.08 (−33.13,−14.15)	−0.43 (−0.99,0.13)
Central Latin America	29.25 (27.33,31.52)	0.07 (0.07,0.08)	28.57 (24.99,32.68)	0.07 (0.06,0.08)	−2.33 (−15.48,12.38)	−6.90 (−19.44,7.12)	0.54 (−0.01,1.10)
Central Sub-Saharan Africa	13.69 (5.36,24.09)	0.09 (0.04,0.16)	26.82 (13.31,45.05)	0.07 (0.04,0.12)	95.85 (21.36,222.04)	−22.34 (−51.88,27.69)	−0.55 (−0.71,−0.39)
East Asia	139.72 (71.53,190.53)	0.07 (0.03,0.09)	27.15 (18.86,42.88)	0.01 (0.01,0.02)	−80.57 (−89.72,−55.86)	−77.79 (−88.25,−49.54)	−5.68 (−6.07,−5.29)
Eastern Europe	7.09 (6.30,8.23)	0.02 (0.02,0.02)	7.01 (6.57,7.43)	0.03 (0.03,0.03)	−1.12 (−14.32,13.49)	33.61 (15.77,53.35)	1.17 (0.62,1.73)
Eastern Sub-Saharan Africa	98.30 (45.31,142.16)	0.18 (0.08,0.26)	156.34 (93.13,225.87)	0.14 (0.08,0.20)	59.05 (9.13,153.41)	−24.41 (−48.13,20.44)	−0.95 (−1.02,−0.89)
High-income Asia Pacific	8.32 (5.32,11.62)	0.03 (0.02,0.05)	2.42 (2.04,2.98)	0.02 (0.01,0.02)	−70.87 (−80.61,−51.35)	−54.44 (−69.67,−23.91)	−3.24 (−3.56,−2.91)
High-income North America	8.16 (7.17,9.68)	0.02 (0.02,0.02)	6.49 (6.06,6.98)	0.01 (0.01,0.02)	−20.48 (−36.09,−5.30)	−29.51 (−43.35,−16.05)	−1.18 (−1.36,−1.01)
North Africa and Middle East	94.65 (61.90,132.09)	0.11 (0.07,0.15)	67.11 (50.90,88.31)	0.05 (0.04,0.07)	−29.10 (−49.82,−0.17)	−48.21 (−63.35,−27.08)	−1.88 (−2.01,−1.76)
Oceania	1.99 (1.18,3.38)	0.12 (0.07,0.20)	6.13 (3.48,10.29)	0.19 (0.11,0.33)	208.41 (82.67,439.45)	64.25 (−2.71,187.29)	2.01 (1.79,2.22)
South Asia	117.75 (62.84,176.83)	0.04 (0.02,0.06)	127.63 (82.93,165.21)	0.04 (0.02,0.05)	8.39 (−21.61,49.24)	−14.04 (−37.83,18.36)	−0.42 (−0.63,−0.20)
Southeast Asia	124.67 (82.03,167.05)	0.11 (0.07,0.15)	90.00 (71.44,124.17)	0.08 (0.06,0.11)	−27.81 (−49.13,9.91)	−30.23 (−50.84,6.21)	−0.92 (−1.03,−0.81)
Southern Latin America	4.51 (3.92,5.19)	0.05 (0.04,0.05)	2.43 (2.07,2.80)	0.02 (0.02,0.03)	−46.15 (−56.65,−34.98)	−48.45 (−58.51,−37.76)	−1.85 (−2.09,−1.60)
Southern Sub-Saharan Africa	19.14 (12.93,24.57)	0.14 (0.10,0.19)	21.38 (16.27,26.88)	0.13 (0.10,0.17)	11.70 (−21.78,45.82)	−7.95 (−35.54,20.17)	−1.03 (−1.49,−0.57)
Tropical Latin America	46.06 (41.50,50.52)	0.13 (0.11,0.14)	24.40 (20.48,27.78)	0.07 (0.06,0.08)	−47.03 (−55.36,−38.80)	−41.33 (−50.56,−32.22)	−1.03 (−1.55,−0.51)
Western Europe	7.62 (6.72,8.94)	0.02 (0.01,0.02)	9.37 (8.60,9.97)	0.02 (0.02,0.02)	22.91 (4.74,43.16)	25.98 (7.36,46.74)	1.21 (0.71,1.71)
Western Sub-Saharan Africa	51.32 (24.69,72.70)	0.10 (0.05,0.14)	131.89 (62.94,186.41)	0.10 (0.05,0.14)	157.00 (93.98,238.39)	−0.61 (−24.98,30.87)	−0.05 (−0.21,0.10)

EAPC, estimated annual percentage change; SDI, sociodemographic index; UI, uncertainty interval.

^a^ EAPC is expressed as 95% confidence interval.

^b^ Change shows the percentage change.

#### DALYs

Total global DALYs due to IE in this age group fell from 65,944.7 (95% UI, 43,484.0–82,413.1) in 1990 to 64,434.2 (95% UI, 48,112.3–76,349.7) in 2021, a 2.29% reduction (95% UI, −19.16% to 23.19%). The DALY rate dropped from 5.89 per 100,000 (95% UI, 3.89–7.36) to 4.76 per 100,000 (95% UI, 3.55–5.64), a decrease of 19.22% (95% UI, −33.17% to 1.86%) with an EAPC of −0.56% (95% CI, −0.66% to −0.46%; Table 3). This decline was particularly marked between 2016 and 2021 (APC −2.74%, 95% CI, −3.10% to −2.32%), with the lowest DALY rate observed in 2021 (Figure 1C).

**Table 3:** DALYs of infective endocarditis in children Between 1990 and 2021 at the global and regional level.

location	1990 (95% UI)		2021 (95% UI)		1990–2021		
	DALYs cases	DALY rate	DALYs cases	DALY rate	Cases change^b^	Rate change^b^	EAPC^a^
Global	65944.74 (43484.04,82413.14)	5.89 (3.89,7.36)	64434.18 (48112.32,76349.69)	4.76 (3.55,5.64)	−2.29 (−19.16,23.19)	−19.22 (−33.17,1.86)	−0.56 (−0.66,−0.46)
High SDI	2590.72 (2301.25, 2915.61)	2.09 (1.85,2.35)	2183.03 (1926.28, 2535.24)	1.84 (1.62,2.14)	−15.74 (−27.24,−3.24)	−11.90 (−23.93,1.16)	−0.33 (−0.54,−0.12)
High-middle SDI	8193.72 (5587.53, 10486.82)	4.53 (3.09,5.80)	3628.50 (3130.15, 4518.41)	2.26 (1.95,2.81)	−55.72 (−68.40,−35.20)	−50.24 (−64.49,−27.20)	−2.25 (−2.40,−2.11)
Middle SDI	25486.87 (17420.47,31095.37)	6.77 (4.62,8.26)	15776.00 (13404.07,18860.85)	4.04 (3.43,4.83)	−38.10 (−51.04,−16.81)	−40.25 (−52.74,−19.70)	−1.42 (−1.56,−1.27)
Low-middle SDI	16411.02 (10570.33,21357.74)	5.50 (3.54,7.15)	17988.06 (13579.22,21671.78)	4.63 (3.50,5.58)	9.61 (−12.18,40.46)	−15.69 (−32.45,8.04)	−0.42 (−0.55,−0.29)
Low SDI	13199.16 (6643.40, 18463.85)	9.56 (4.81,13.37)	24777.46 (14474.43,33642.99)	8.41 (4.91,11.42)			
Regions					87.72 (43.26,168.69)	−12.01 (−32.85,25.95)	−0.48 (−0.55,−0.41)
Andean Latin America	990.02 (676.67,1313.85)	10.34 (7.07,13.73)	895.93 (703.75,1157.47)	7.50 (5.89,9.69)	−9.50 (−37.69,29.01)	−27.46 (−50.05,3.42)	−0.82 (−1.04,−0.60)
Australasia	28.24 (24.53,32.44)	0.93 (0.81,1.07)	46.41 (38.38,56.35)	1.19 (0.98,1.44)	64.38 (35.23,100.70)	27.79 (5.13,56.03)	0.74 (0.35,1.13)
Caribbean	659.84 (501.35,898.76)	9.06 (6.89,12.34)	839.92 (550.49, 1175.07)	11.00 (7.21,15.39)	27.29 (−10.29,82.33)	21.36 (−14.47,73.83)	0.75 (0.68,0.82)
Central Asia	265.50 (216.74,339.91)	1.72 (1.40,2.20)	194.36 (164.09,226.47)	1.10 (0.93,1.28)	−26.79 (−44.05,−5.09)	−35.95 (−51.05,−16.96)	−1.53 (−1.92,−1.14)
Central Europe	344.06 (314.35,390.39)	1.69 (1.54,1.92)	203.38 (172.72,245.57)	1.68 (1.43,2.03)	−40.89 (−49.60,−31.40)	−0.70 (−15.34,15.23)	0.30 (−0.19,0.79)
Central Latin America	2475.42 (2310.73, 2666.36)	5.98 (5.59,6.45)	2476.53 (2189.70, 2861.99)	5.71 (5.05,6.60)	0.05 (−12.83,14.72)	−4.64 (−16.91,9.35)	0.59 (0.04,1.15)
Central Sub-Saharan Africa	1125.17 (455.70, 1958.75)	7.54 (3.06,13.13)	2250.77 (1147.52, 3747.98)	5.98 (3.05,9.96)	100.04 (25.57,219.97)	−20.69 (−50.21,26.87)	−0.49 (−0.64,−0.34)
East Asia	11485.88 (5959.29, 15585.43)	5.37 (2.78,7.28)	2378.71 (1713.19, 3657.11)	1.27 (0.91,1.95)	−79.29 (−88.74,−54.82)	−76.33 (−87.13,−48.36)	−5.48 (−5.87,−5.09)
Eastern Europe	618.76 (547.16,719.72)	1.81 (1.60,2.10)	701.48 (633.23,793.05)	2.77 (2.50,3.13)	13.37(−3.16,30.60)	53.19 (30.85,76.47)	1.57 (1.11,2.04)
Eastern Sub-Saharan Africa	8068.90 (3900.23, 11583.52)	14.81 (7.16,21.26)	13100.95 (8165.77, 18516.87)	11.43 (7.12,16.15)	62.36 (12.83,154.40)	−22.83 (−46.38,20.91)	−0.88 (−0.94,−0.82)
High-income Asia Pacific	701.36 (460.31,953.91)	2.81 (1.84,3.82)	258.35 (217.55,313.17)	1.62 (1.36,1.96)	−63.16 (−75.46,−42.32)	−42.39 (−61.62,−9.78)	−2.43 (−2.74,−2.13)
High-income North America	744.18 (645.81,887.81)	1.86 (1.61,2.22)	669.92 (597.93,769.66)	1.48 (1.33,1.71)	−9.98 (−26.78,5.75)	−20.20 (−35.10,−6.26)	−0.81 (−0.97,−0.64)
North Africa and Middle East	7739.26 (5106.98, 10,763.54)	8.67 (5.72,12.06)	5655.06 (4330.79, 7340.26)	4.63 (3.54,6.01)	−26.93 (−47.43,2.36)	−46.62 (−61.60,−25.23)	−1.79 (−1.90,−1.67)
Oceania	156.38 (93.40,265.19)	9.33 (5.57,15.83)	481.99 (275.40,804.99)	15.32 (8.75,25.58)	208.22 (83.99,435.81)	64.15 (−2.01,185.36)	2.00 (1.79,2.22)
South Asia	9645.53 (5252.84, 14466.44)	3.49 (1.90,5.24)	10685.60 (7164.89, 13697.61)	3.07 (2.06,3.93)	10.78 (−19.95,52.17)	−12.14 (−36.51,20.69)	−0.35 (−0.56,−0.14)
Southeast Asia	10139.93 (6697.25, 13608.44)	9.02 (5.96,12.10)	7409.54 (5929.89, 10132.09)	6.37 (5.10,8.71)	−26.93 (−48.01,10.07)	−29.38 (−49.76,6.37)	−0.89 (−1.00,−0.78)
Southern Latin America	382.30 (336.13,437.15)	3.91 (3.44,4.47)	219.76 (189.39,252.05)	2.15 (1.85,2.47)	−42.52 (−52.80,−31.51)	−44.98 (−54.82,−34.44)	−1.69 (−1.91,−1.47)
Southern Sub-Saharan Africa	1606.24 (1093.99, 2060.33)	12.15 (8.28,15.59)	1802.21 (1399.19, 2245.79)	11.24 (8.72,14.00)	12.20 (−19.54,44.87)	−7.53 (−33.69,19.40)	−0.94 (−1.35,−0.52)
Tropical Latin America	3854.75 (3472.09, 4225.41)	10.55 (9.50,11.57)	2145.88 (1811.24, 2448.33)	6.51 (5.49,7.42)	−44.33 (−52.81,−36.05)	−38.34 (−47.74,−29.17)	−0.92 (−1.41,−0.42)
Western Europe	699.25 (610.18,827.00)	1.45 (1.27,1.72)	1070.00 (924.12, 1281.66)	2.28 (1.97,2.73)	53.02 (27.26,80.80)	56.85 (30.44,85.32)	1.95 (1.51,2.40)
Western Sub-Saharan Africa	4213.77 (2059.93, 5914.92)	8.08 (3.95,11.35)	10947.43 (5457.36, 15273.82)	8.12 (4.05,11.33)	159.80 (97.01,240.39)	0.48 (−23.81,31.64)	−0.02 (−0.17,0.13)

DALYs, disability adjusted life years; EAPC, estimated annual percentage change; SDI, sociodemographic index; UI, uncertainty interval.

^a^ EAPC is expressed as 95% confidence interval.

^b^ Change shows the percentage change.

### Trends by sociodemographic index (SDI)

#### Incidence

**Figure 2:**
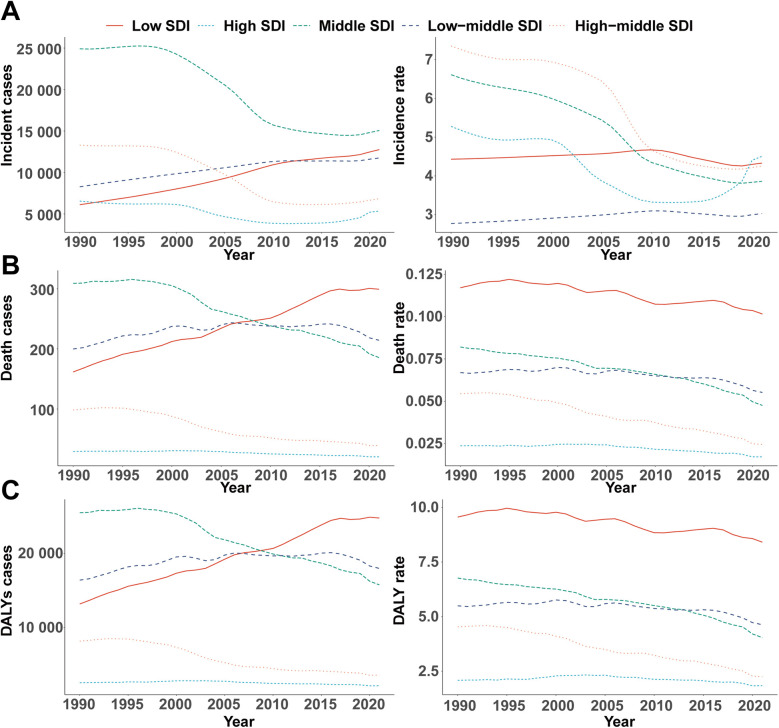
Epidemiologic trends in childhood infective endocarditis incidence, mortality, and disability-adjusted life year (DALY) rates across five sociodemographic index areas from 1990 to 2021. **(A)** Incidence cases and rates. **(B)** Death cases and rates. **(C)** DALYs cases and rates.

Incidence patterns diverged across SDI quintiles. High-SDI regions experienced a modest reduction in case numbers (from 6,549 [95% UI, 4,188–10,128] to 5,349 [95% UI, 3,548–8,008]) and a slight decline in incidence rate (from 5.28 [95% UI, 3.37–8.16] to 4.51 [95% UI, 2.99–6.75] per 100,000). Notably, incidence rates decreased from 1990 to 2011 before rebounding after 2015, reaching 4.51 per 100,000 in 2021 (Figure 2A). Middle-high and middle SDI regions saw the most pronounced declines (−42.16% and −41.63%, respectively), whereas incidence rose in low-middle SDI regions by 9.28% (95% UI, 5.86–13.22%). EAPC varied, with high (−1.41%), middle-high (−2.28%), and middle (−2.12%) SDI areas experiencing significant declines, and low-middle (0.29%) and low SDI regions (−0.07%) exhibiting stable or minimal change (Table 1).

#### Mortality

Marked disparities emerged across SDI quintiles. High-SDI regions saw a decline in deaths (from 29 [95% UI, 26–33] to 20 [95% UI, 19–22]), while deaths in low-SDI regions nearly doubled (from 162 [95% UI, 80–228] to 299 [95% UI, 170–416]). EAPC in high (−0.98%), middle-high (−2.57%), and middle (−1.51%) SDI regions reflected greater declines, while low (−0.53%) and low-middle (−0.48%) SDI areas exhibited smaller reductions (Table 2). Throughout 1990–2021, mortality rates in low-SDI regions remained five times higher than those in high-SDI regions (Figure 2B).

#### DALYs

DALY trends paralleled those observed for incidence and mortality. High-SDI regions experienced a 15.74% reduction in DALYs (from 2,591 [95% UI, 2,301–2,916] to 2,183 [95% UI, 1,926–2,535]), with the steepest relative decrease in middle-high SDI regions (−55.72%, 95% UI, −68.40% to −35.20%). Conversely, DALYs in low-SDI regions surged by 87.72% (95% UI, 43.26%–168.69%). In 2021, the DALY rate in low-SDI areas was 8.41 per 100,000 (95% UI, 4.91–11.42), more than 4.6 times higher than in high-SDI regions (1.84 per 100,000, 95% UI, 1.62–2.14; Figure 2C).

### Regional and national patterns

#### Regional incidence, mortality, and DALYs

**Figure 3:**
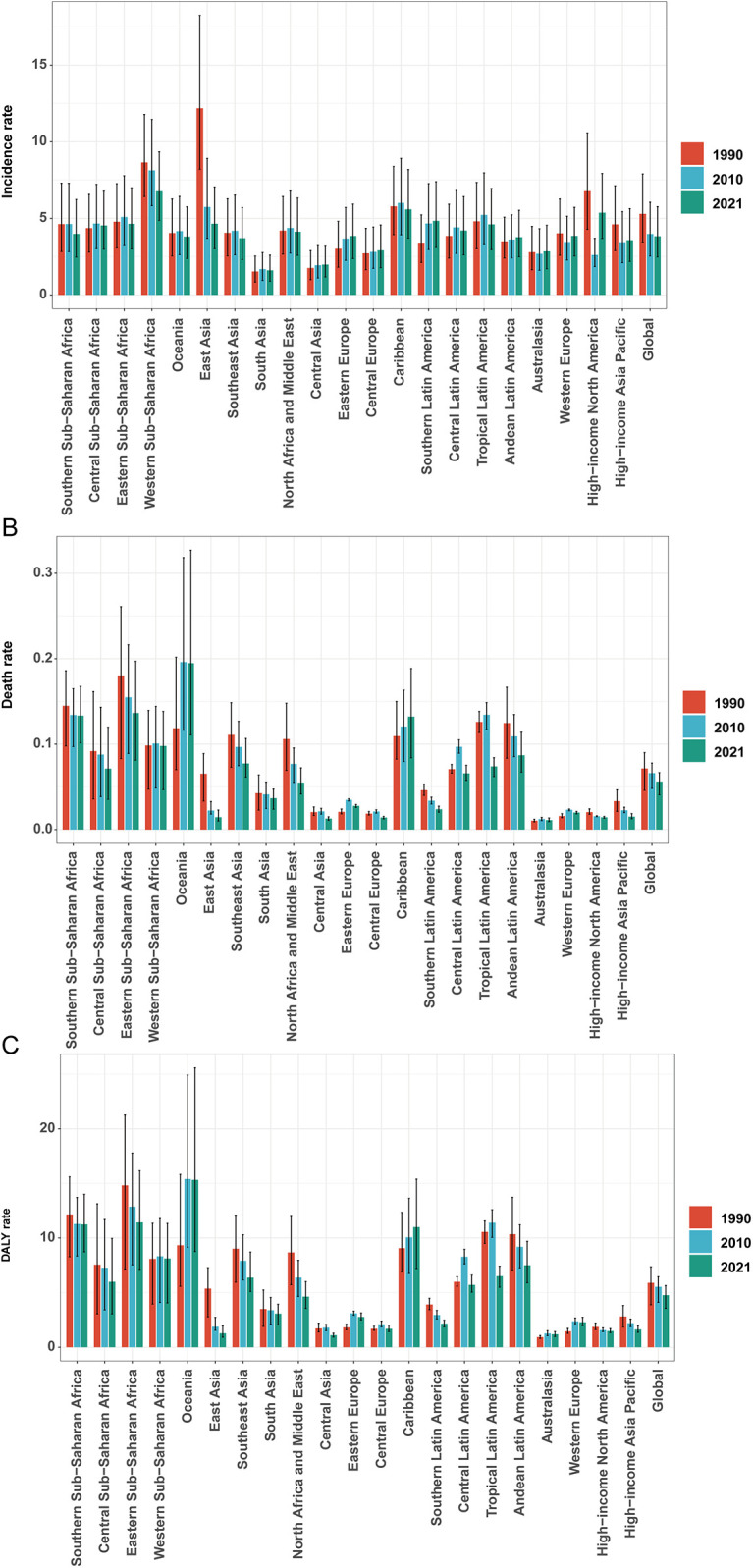
Incidence/mortality/DALY rate of infective endocarditis in children by different years. **(A)** Incidence rate. **(B)** Mortality rate. **(C)** DALY rate.

**Figure 4:**
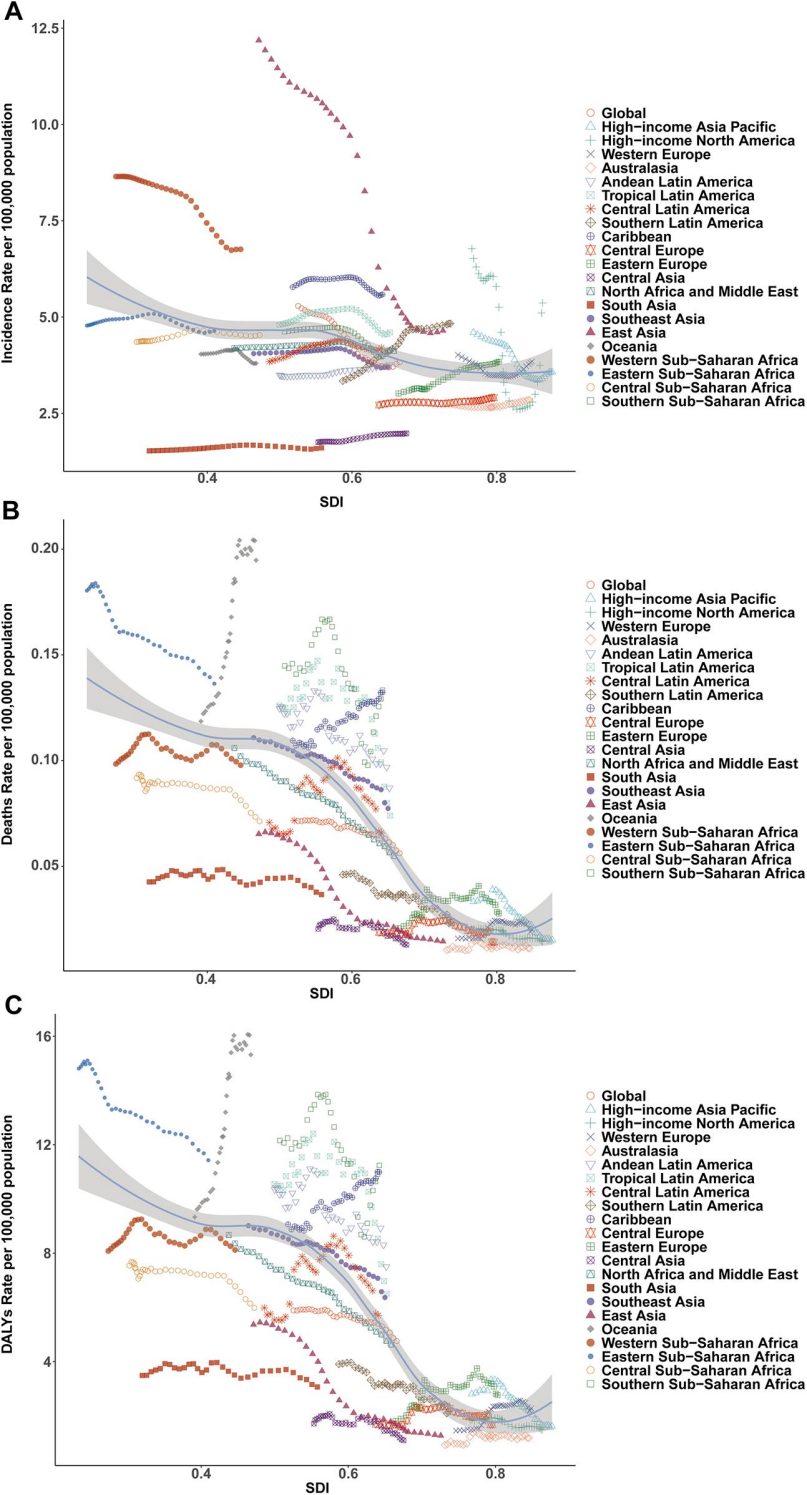
Association of incidence, mortality, and disability-adjusted life year (DALY) rates of pediatric infective endocarditis with the socio-demographic Index (SDI) by region, 1990–2021. **(A)** Incidence rate. **(B)** Mortality rate. **(C)** DALY rate.

East Asia exhibited the largest reduction in incidence (from 12.18 [95% UI, 8.20–18.24] to 4.65 [95% UI, 3.01–7.05] per 100,000), while Western Sub-Saharan Africa had the highest rate in 2021 (6.76 [95% UI, 4.87–9.34] per 100,000), with case numbers more than doubling. Incidence rates rose markedly in Eastern Europe (27.39%), Southern Latin America (44.61%), and Central Asia (13.18%), but fell in East Asia (−61.87%) and Western Sub-Saharan Africa (−21.83%). Declines in EAPC were most marked in East Asia (−3.79%) and high-income North America (−2.75%), whereas Southern Latin America (1.27%) and Eastern Europe (0.89%) displayed increasing trends (Table 1, Figures 3A, 4A).

**Figure 5:**
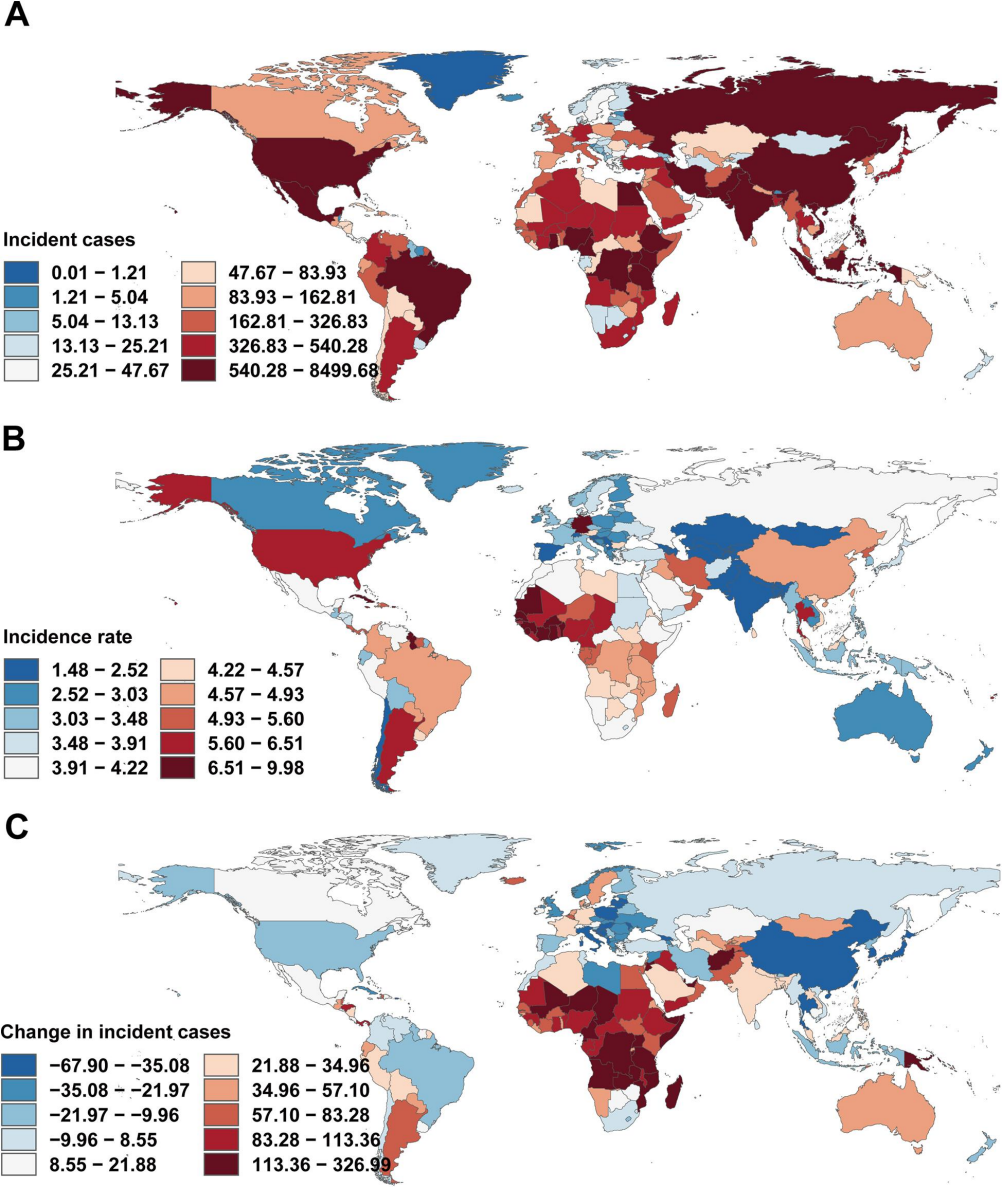
Incidence of infective endocarditis in children across 204 countries and territories. **(A)** Number of incidence cases. **(B)** Incidence rate. **(C)** Change in incident cases. World maps generated using the rnaturalearth R package, https://github.com/ropensci/rnaturalearth.

Mortality rates fell most in East Asia (−77.79%), but rose steeply in Oceania (64.25%) and Sub-Saharan Africa. The greatest reductions in EAPC were observed in East Asia (−5.68%) and high-income Asia Pacific (−3.24%), while Oceania (2.01%), the Caribbean (0.74%), and Eastern Europe (1.17%) saw increases (Table 2, Figures 3B, 4B).

DALY trends mirrored incidence and mortality, with East Asia showing the greatest decrease (−79.29%), and substantial increases in Western Sub-Saharan Africa (159.80%) and Oceania (208.22%). DALY rates rose sharply in Western Europe (56.85%) and increased in Oceania and Eastern Europe, while East Asia demonstrated the greatest decline (EAPC −5.48%; Table 3, Figures 3C, 4C).

#### National variation

At the national level, India (4,053 cases), China (8,416 cases), and Nigeria (4,200 cases) had the highest IE case counts in 2021. Incidence increased substantially in India and Nigeria, but declined sharply in China (−67.22%). Notably, trends in high-income countries with SDI >0.8 were heterogeneous, with declines in Norway (EAPC −2.14) and increases in Malta (EAPC 2.18). Most low-income countries (SDI <0.3) exhibited negative EAPC values (e.g., Niger, EAPC −1.23) (Figure 5A).

For mortality, the most marked decreases were observed in China (−56.10%) and Thailand, while Nigeria and Pakistan saw pronounced increases. EAPC for mortality rates correlated positively with absolute mortality, especially in middle-high SDI countries (Figure 5B).

**Figure 6:**
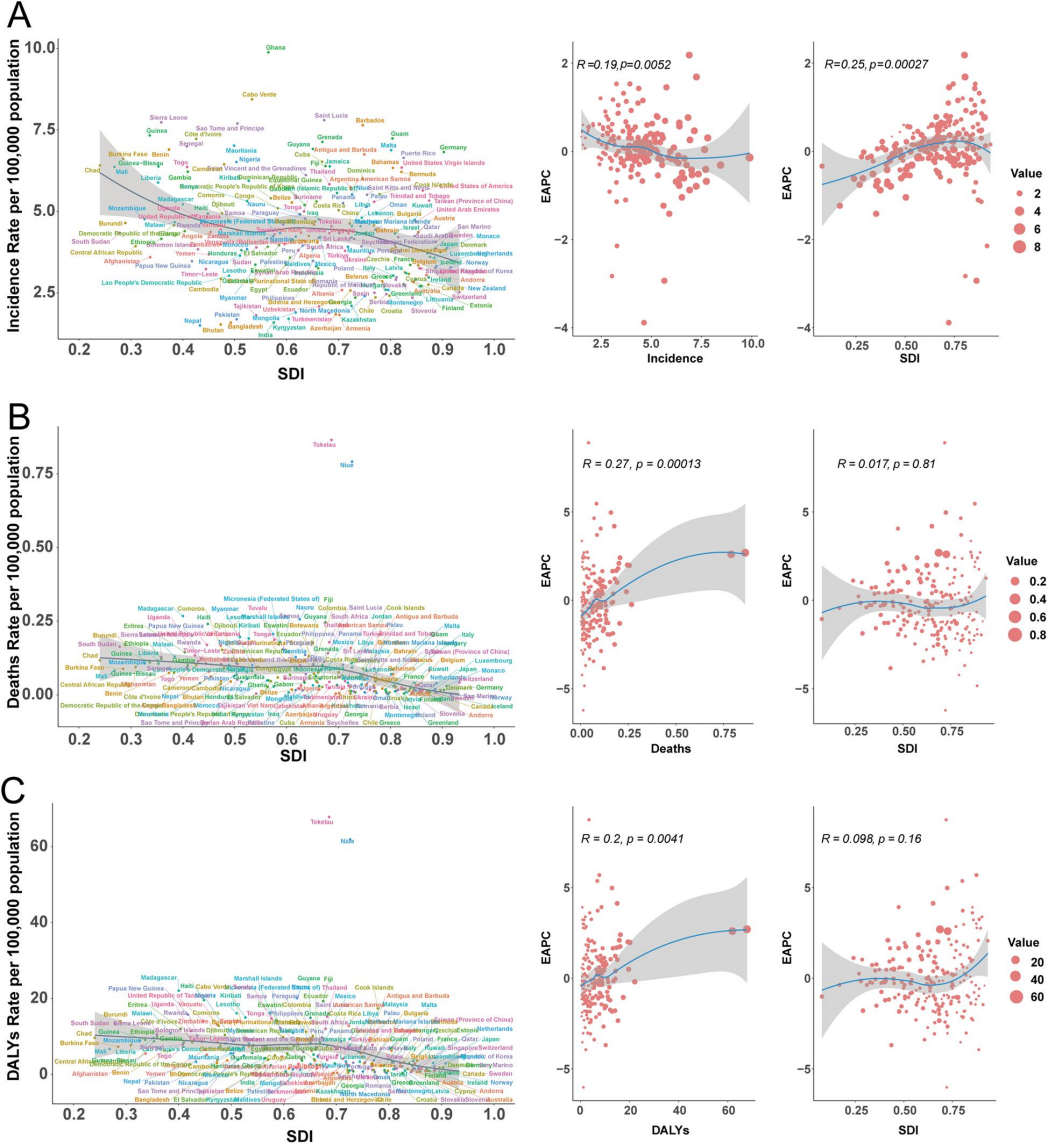
Incidence, mortality, and disability-adjusted life year (DALY) rates of pediatric infective endocarditis by socio-demographic index (SDI) in 204 countries, 2021. **(A)** Incidence rate. **(B)** Mortality rate. **(C)** DALY rate.

DALY rates decreased sharply in China (−6.02%), Egypt, and Thailand, but increased in Australia, Germany, and the UK (Figure 5C). The strongest DALY increases were seen in American Samoa (EAPC 4.97). EAPC for DALY rate displayed a non-linear relationship with SDI, with higher EAPC in both some high-SDI (e.g., Germany) and certain middle-SDI (e.g., Zimbabwe) countries, but generally negative in low-SDI settings (Figures 6A–C).

### Projected trends to 2035 (ARIMA analysis)

#### Incidence

Time series analysis using ARIMA models projected distinct trends by sex. Among males, incidence rose steadily during 1990–1996 (annual increase ∼1.0%), declined between 1997 and 2011 (annual decrease 1.4%), and stabilised thereafter, with a minor uptick post-2012. A transient peak was observed in 2020 (+3.4% over the prior year), followed by a slow projected increase, reaching 30,194 (95% CI: 22,750–37,638) by 2035.

Among females, the incidence trend was more variable, with modest growth from 1990 to 1996 (annual +0.2%), sharp decline from 1997 to 2009 (annual −2.5%), and renewed growth after 2010. Projections suggest a higher future growth rate for females (annual +1.5% during 2022–2035), but with widening uncertainty intervals. By 2035, the predicted incidence is 27,355 (95% CI: 14,973–39,736) for females.

**Figure 7:**
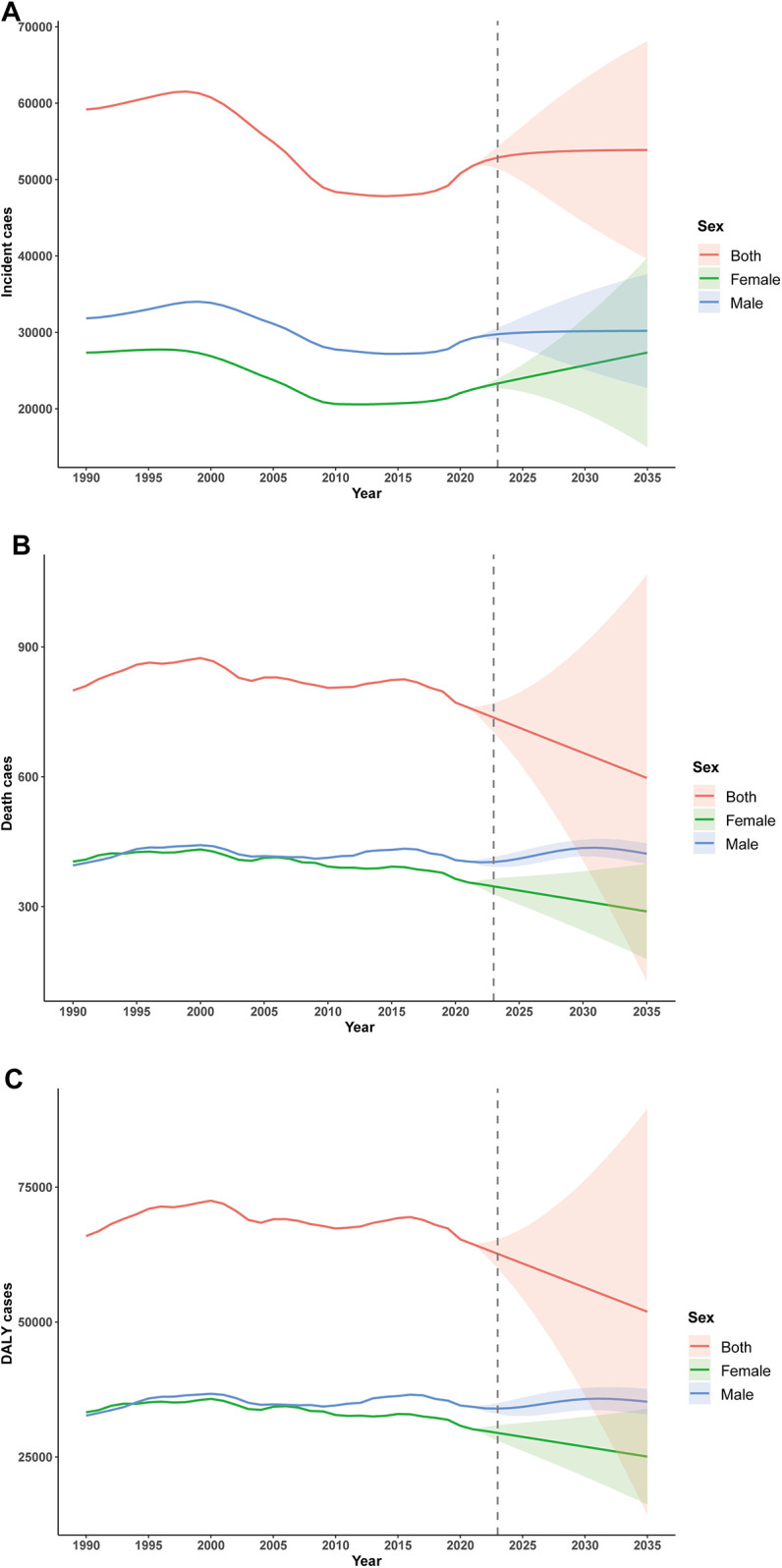
ARIMA-based forecasting of childhood infective endocarditis incidence, mortality and disability-adjusted life years (DALYs) until 2035. **(A)** Incident cases. **(B)** Number of deaths. **(C)** Number of DALYs.

Combined, the overall trend mimics that of males, with a projected value of 53,875 (95% CI: 39,627–68,124) in 2035. The forecast intervals broaden with time, indicating increasing long-term uncertainty (Figure 7A).

#### Mortality

Projected mortality displayed sex-specific patterns. For males, mortality rose from 1990 to 1996, plateaued through 2000, and declined after 2001, reaching 407.5 in 2020. Projections indicate a renewed increase, peaking at 435.5 in 2030 before a gradual decline. Among females, mortality decreased consistently from 404.1 in 1990 to 355.9 in 2021, with projections indicating a continued annual decline to 288.9 by 2035, albeit with increasing uncertainty (Figure 7B).

#### DALYs

DALY projections reflected similar sex-dependent patterns. In males, DALYs increased from 1990 to 1996 (annual +1.8%), declined from 1997 to 2009 (annual −0.6%), and fluctuated thereafter, with an anomalous drop in 2020, potentially related to the COVID-19 pandemic. Projections suggest stable values between 35,000 anf 36,000 through 2035, though uncertainty intervals widen substantially (interval width 4,726.6 in 2035). In females, DALYs declined 9.4% overall (annual −0.3%), with a marked acceleration post-2008 (annual −1.1%). The projected decline continues through 2035 (predicted value 25,068; 16.8% decrease from 2021), again with expanding uncertainty (interval width 17,759.9 in 2035; Figure 7C).

## Discussion

This global analysis of pediatric IE in children aged 5–14 demonstrates a significant decline in incidence, mortality, and DALYs between 1990 and 2021, though considerable regional disparities persist. Notably, progress has not been uniform across socio-demographic development levels. High-SDI regions have achieved the greatest reductions in pediatric IE rates, whereas lower-SDI regions continue to experience relatively high burdens. East Asia—primarily driven by the Chinese population—serves as a successful exemplar of rapid improvement: this region demonstrated the most pronounced decline in mortality rates (62.83%) from 1990 to 2021, which aligns with the marked reduction in DALYs during the study period. By contrast, regions such as sub-Saharan Africa and Eastern Europe have seen far more modest gains, and in some metrics, even regressions. For example, Eastern Europe experienced one of the highest increases in IE burden (as measured by DALYs) since 1990, signaling that pediatric IE remains an escalating problem there. In sub-Saharan Africa, our findings indicate a rising or at best plateauing pediatric IE burden, which aligns with broader evidence that IE morbidity and mortality in developing regions have not decreased in recent years. In aggregate, children in low-SDI settings still face a substantially higher risk of dying from IE and suffer more years of healthy life lost per infection, compared to their peers in high-SDI countries. This disparity is reflected in high DALY-to-incidence ratios in many low- and lower-middle-income countries, where limited access to diagnostics and treatment often results in poor outcomes for each case of IE.

The shifting epidemiology of pediatric IE likely reflects both improvements in underlying risk factors and emergent new risks associated with modern medical care. Historically, RHD was a dominant precursor of endocarditis in children (24); repeated streptococcal infections leading to valvular damage created a fertile ground for viridans streptococci and other bacteria to seed the heart valves (25). Today, RHD has nearly vanished in high-income countries, and even globally, its role as a predisposing factor in childhood IE has diminished with socio-economic development and widespread antibiotic use (26). Concurrently, the nature of pediatric heart disease has changed. Far more children with CHD now survive infancy and early childhood due to advances in surgery and intensive care. While this is a public health triumph, it also means a growing cohort of children living with repaired or palliated CHD who remain at risk for IE (7, 27, 28). In high-income settings, the majority of pediatric IE cases now occur in children with congenital cardiac lesions (especially those with prosthetic material or residual defects from surgery) (29, 30). The improved survival of CHD patients into mid-childhood has thus shifted the pediatric IE landscape toward healthcare-associated cases, whereas the traditional post-streptococcal (RHD-driven) endocarditis has receded in many regions.

Healthcare advances have also introduced new iatrogenic risk factors for endocarditis in children. The increased use of central venous catheters, long-term indwelling lines, and other invasive devices in pediatric intensive care and oncology has led to more cases of nosocomial IE caused by opportunistic and hospital-acquired pathogens (31). In developing countries, too, the microbiological pattern is evolving. Over the past few decades, the proportion of staphylococcal IE in low-income regions has significantly increased (from ∼15% to ∼24% of cases) while the fraction attributed to viridans streptococci has also grown with better detection (32). This likely reflects greater awareness and diagnostic capacity for IE, as well as the ongoing challenges of healthcare-associated bacteremia. Meanwhile, the decline of RHD in some regions has reduced the pool of classic streptococcal endocarditis, although RHD-related IE persists where rheumatic fever remains common. In summary, pediatric IE etiology is transitioning from a disease largely triggered by community-acquired bacteremia in predisposed hosts (RHD or unrepaired CHD) to one often driven by healthcare-related factors—survival with medical hardware, frequent hospital contact, and recurrent bacteremias in medically complex children.

The pathophysiology of IE in children mirrors that in adults and is rooted in the interplay between an altered cardiac endothelium and circulating microbes. Endocardial endothelial surfaces are normally resistant to infection, but any disruption, such as the turbulent blood flow across a malformed or damaged valve, or the presence of a foreign body (e.g., prosthetic patch or catheter), can initiate a cascade leading to IE (33). Turbulence or trauma to the endocardium causes microscopic injury and triggers the deposition of fibrin and platelets, forming a sterile thrombus known as non-bacterial thrombotic endocarditis (NBTE) (34). This fibrin-platelet matrix provides an ideal nidus for bacteria. During episodes of bacteremia, even of brief duration and mundane origin (such as tooth brushing or minor skin infections), circulating microbes can adhere to the NBTE via surface adhesin proteins (35, 36). *S. aureus* and *Streptococcus* species possess adhesins (e.g., MSCRAMMs) that bind host matrix molecules like fibrin, allowing them to latch onto injured valve tissue. Once attached, bacteria become enmeshed in the growing vegetation, which matures into a complex biofilm. Indeed, IE vegetations are essentially biofilms on the endocardium, aggregates of microorganisms encased in a self-produced extracellular matrix of platelets, fibrin, and cellular debris (37). This biofilm architecture confers remarkable protection: bacteria deep within the vegetation are metabolically quiescent and shielded from both antibiotics and host immune effectors. Even prolonged, high-dose antibiotic therapy may fail to fully eradicate these entrenched microcolonies, which explains the frequent need for surgical debridement in IE and the propensity for relapse if any infectious nidus remains (38). The host immune response, while well-intentioned, can exacerbate the pathology. Platelet activation and recruited neutrophils form extracellular traps (NETs) within the vegetation, contributing to valve tissue destruction and embolism risk (39). Thus, the pathogenesis of IE involves a vicious cycle: endothelial injury begets a thrombotic focus for infection, bacteria colonize and create a biofilm, and the ensuing immune/inflammatory reaction further damages the valve and facilitates persistent infection. These mechanisms underscore why prevention is paramount—once a mature IE vegetation has formed, it is difficult to cure and prone to cause severe complications.

Looking ahead, our time-series forecasts (using ARIMA modeling) project a potential stagnation or even modest rebound in the global pediatric IE burden by 2035. After decades of decline, the incidence of pediatric IE is predicted to plateau, and in certain regions or subpopulations, it may increase. One notable projection is a relative uptick in IE among female children. Historically, IE has been more common in males, a pattern often attributed to the higher prevalence of congenital heart anomalies in boys and possibly gender differences in healthcare exposure or behavior (40). However, our model suggests that the gap between sexes could narrow in the coming years. By 2035, the incidence and DALYs in 5–14-year-old girls are forecasted to rise slightly faster than in boys, potentially eroding the male predominance seen in past data. This finding resonates with some recent observations in adult IE: while men have higher incidence, female patients tend to have worse outcomes and increasing representation in older age groups (41). In pediatrics, a convergence might indicate that improvements that benefited male children (e.g., early intervention for critical CHD, which boys more commonly have) are now reaching female patients as well, equalizing exposure to IE risk. It could also reflect sociocultural shifts, for instance, better access to care for girls in regions where they previously faced healthcare disparities. Nonetheless, caution is warranted in interpreting this sex-specific trend. The absolute differences projected are small, and the reasons are not fully elucidated; they may partly stem from model uncertainty. Further studies are needed to see if female pediatric IE truly rises or if this is an artifact of forecasting.

This study, based on GBD 2021 estimates and forecasts, has several limitations that must be acknowledged when interpreting the results. Data quality and availability present the foremost challenge. Reliable statistics on pediatric IE are scarce in many low- and middle-income countries. GBD compensates for missing data through modeling and covariate adjustments, but these approaches can only approximate reality. If primary data are absent or of poor quality (for example, due to misclassification of IE as generic sepsis or underdiagnosis), the GBD estimates for those locations may carry substantial uncertainty. The modeling process might not capture localized outbreaks or unique risk factors. For instance, if a country has an undiagnosed increase in IE due to injection drug use among teenagers, that signal could be missed in global estimates. Coding variability is another concern: the diagnosis of “IE” can be inconsistently recorded across health systems, especially where advanced diagnostic tools are lacking. Some children who died of IE might be coded as having rheumatic fever, heart failure, or septicemia on death certificates, leading to underestimation of true IE mortality. Conversely, improvements in surveillance over time can create an artificial impression of rising incidence (detection bias). We attempted to mitigate these issues by focusing on age-specific trends and using validated modeling techniques, but the potential for bias remains.

In summary, from 1990 to 2021, the global incidence rate, mortality, and DALYs of IE in children demonstrated an overall declining trend. However, the disease burden continued to rise in certain low SDI regions. Consequently, policymakers must develop more effective prevention and control measures to reduce the disease burden of pediatric IE, improve family well-being, and alleviate socioeconomic pressures.

## Conclusion

This global analysis reveals a significant decline in pediatric IE incidence, mortality, and DALY rates among children aged 5–14 years over the past 30 years, though substantial disparities persist across SDI quintiles and geographic regions. While high-middle SDI nations, notably China, have made exceptional strides, low- and middle-income regions, particularly in sub-Saharan Africa and Eastern Europe, continue to face disproportionately high or rising burdens. The results highlight critical gaps in prevention strategies, diagnostic capacity, and specialized cardiac care for children in resource-poor settings. Strengthening health systems, implementing rigorous surveillance, and formulating evidence-based policies are crucial to maintaining global gains and addressing inequities in IE outcomes. National and international child health initiatives must prioritize RHD control, CHD management, and endocarditis prophylaxis to mitigate this preventable yet devastating condition worldwide.

## Data Availability

Publicly available datasets were analyzed in this study. This data can be found here: https://vizhub.healthdata.org/gbd-results/.

## References

[1] Mahony M Lean D Pham L Horvath R Suna J Ward C Infective endocarditis in children in Queensland, Australia: epidemiology, clinical features and outcome. Pediatr Infect Dis J. (2021) 40(7):617–22. 10.1097/inf.0000000000003110 33902079

[2] Pediatric Bacterial Endocarditis. (2019). Available online at: https://emedicine.medscape.com/article/896540-overview#a6 (Accessed May 16, 2025).

[3] Cahill TJ Prendergast BD. Infective endocarditis. Lancet. (2016) 387(10021):882–93. 10.1016/s0140-6736(15)00067-7 26341945

[4] Tonelli A Lumngwena EN Ntusi NAB. The oral microbiome in the pathophysiology of cardiovascular disease. Nat Rev Cardiol. (2023) 20(6):386–403. 10.1038/s41569-022-00825-3 36624275

[5] Sambola A Lozano-Torres J Boersma E Olmos C Ternacle J Calvo F Predictors of embolism and death in left-sided infective endocarditis: the European Society of Cardiology EURObservational research programme European infective endocarditis registry. Eur Heart J. (2023) 44(43):4566–75. 10.1093/eurheartj/ehad507 37592753

[6] Karthikeyan G Ntsekhe M Islam S Rangarajan S Avezum A Benz A Mortality and morbidity in adults with rheumatic heart disease. JAMA. (2024) 332(2):133–40. 10.1001/jama.2024.8258 38837131 PMC11154374

[7] Havers-Borgersen E Østergaard L Holgersson CK Stahl A Schmidt MR Smerup M Infective endocarditis with or without congenital heart disease: clinical features and outcomes. Eur Heart J. (2024) 45(44):4704–15. 10.1093/eurheartj/ehae548 39217474

[8] Baltimore RS Gewitz M Baddour LM Beerman LB Jackson MA Lockhart PB Infective endocarditis in childhood: 2015 update: a scientific statement from the American heart association. Circulation. (2015) 132(15):1487–515. 10.1161/cir.0000000000000298 26373317

[9] Luca AC Curpan AS Adumitrachioaiei H Ciobanu I Dragomirescu C Manea RS Difficulties in diagnosis and therapy of infective endocarditis in children and adolescents-cohort study. Healthcare. (2021) 9(6):760. 10.3390/healthcare9060760 34205298 PMC8235031

[10] Woodruff RC Eliapo-Unutoa I Chiou H Gayapa M Noonan S Podila PSB Period prevalence of rheumatic heart disease and the need for a centralized patient registry in American Samoa, 2016 to 2018. J Am Heart Assoc. (2021) 10(20):e020424. 10.1161/jaha.120.020424 34612073 PMC8751893

[11] Kalezi ZE Simbila AN Nkya DA Kubhoja SD Majani NG Furia FF Infective endocarditis in children with heart diseases at Jakaya Kikwete cardiac institute, Tanzania: a cross-sectional study. BMC Pediatr. (2024) 24(1):612. 10.1186/s12887-024-05091-5 39342252 PMC11438279

[12] Lin L Xu J Chai Y Wu W. Global, regional, and national burden of infective endocarditis from 2010 to 2021 and predictions for the next five years: results from the global burden of disease study 2021. BMC Public Health. (2025) 25(1):1115. 10.1186/s12889-025-22100-z 40128765 PMC11934598

[13] Miao H Zhou Z Yin Z Li X Zhang Y Zhang Y Global trends and regional differences in the burden of infective endocarditis, 1990–2021: an analysis of the global burden of disease study 2021. J Epidemiol Glob Health. (2025) 15(1):69. 10.1007/s44197-025-00413-x 40327304 PMC12055685

[14] Murray CJL. The global burden of disease study at 30 years. Nat Med. (2022) 28(10):2019–26. 10.1038/s41591-022-01990-1 36216939

[15] Rezaei F Mazidimoradi A Rayatinejad A Allahqoli L Salehiniya H. Temporal trends of tracheal, bronchus, and lung cancer between 2010 and 2019, in Asian countries by geographical region and sociodemographic index, comparison with global data. Thorac Cancer. (2023) 14(18):1668–706. 10.1111/1759-7714.14912 37127553 PMC10290923

[16] Zi H Liu MY Luo LS Huang Q Luo PC Luan HH Global burden of benign prostatic hyperplasia, urinary tract infections, urolithiasis, bladder cancer, kidney cancer, and prostate cancer from 1990 to 2021. Mil Med Res. (2024) 11(1):64. 10.1186/s40779-024-00569-w 39294748 PMC11409598

[17] Zhou M Wang H Zeng X Yin P Zhu J Chen W Mortality, morbidity, and risk factors in China and its provinces, 1990–2017: a systematic analysis for the global burden of disease study 2017. Lancet. (2019) 394(10204):1145–58. 10.1016/s0140-6736(19)30427-1 31248666 PMC6891889

[18] Vos T Lim SS Abbafati C Abbas KM Abbasi M Abbasifard M Global burden of 369 diseases and injuries in 204 countries and territories, 1990–2019: a systematic analysis for the global burden of disease study 2019. Lancet. (2020) 396(10258):1204–22. 10.1016/s0140-6736(20)30925-9 33069326 PMC7567026

[19] Ben Khadda Z Fagroud M El Karmoudi Y Ezrari S Elhanafi L Radu AF Association between pesticide exposure and end-stage renal disease: a case-control study from Morocco based on the STROBE guidelines. Ecotoxicol Environ Saf. (2024) 288:117360. 10.1016/j.ecoenv.2024.117360 39566262

[20] Kocarnik JM Compton K Dean FE Fu W Gaw BL Harvey JD Cancer incidence, mortality, years of life lost, years lived with disability, and disability-adjusted life years for 29 cancer groups from 2010 to 2019: a systematic analysis for the global burden of disease study 2019. JAMA Oncol. (2022) 8(3):420–44. 10.1001/jamaoncol.2021.6987 34967848 PMC8719276

[21] Qiu H Cao S Xu R. Cancer incidence, mortality, and burden in China: a time-trend analysis and comparison with the United States and United Kingdom based on the global epidemiological data released in 2020. Cancer Commun. (2021) 41(10):1037–48. 10.1002/cac2.12197 PMC8504144 34288593

[22] Kang L Jing W Liu J Liu M. Trends of global and regional aetiologies, risk factors and mortality of lower respiratory infections from 1990 to 2019: an analysis for the global burden of disease study 2019. Respirology. (2023) 28(2):166–75. 10.1111/resp.14389 36210345

[23] Wafa HA Marshall I Wolfe CDA Xie W Johnson CO Veltkamp R Burden of intracerebral haemorrhage in Europe: forecasting incidence and mortality between 2019 and 2050. Lancet Reg Health Eur. (2024) 38:100842. 10.1016/j.lanepe.2024.100842 38362494 PMC10867656

[24] Sogukpinar Onsuren A Gullu UU İpek S. Oral health experiences of Turkish children with acute rheumatic fever or rheumatic heart disease. Eur Oral Res. (2022) 56(1):28–34. 10.26650/eor.2022868100 35478704 PMC9012219

[25] Chamat-Hedemand S Dahl A Østergaard L Arpi M Fosbøl E Boel J Prevalence of infective endocarditis in streptococcal bloodstream infections is dependent on streptococcal Species. Circulation. (2020) 142(8):720–30. 10.1161/circulationaha.120.046723 32580572

[26] Watkins DA Johnson CO Colquhoun SM Karthikeyan G Beaton A Bukhman G Global, regional, and national burden of rheumatic heart disease, 1990–2015. N Engl J Med. (2017) 377(8):713–22. 10.1056/NEJMoa1603693 28834488

[27] Caselli S Attenhofer Jost C Greutmann M. Infective endocarditis in congenital heart disease: the expected and the unexpected. Eur Heart J. (2024) 45(44):4716–8. 10.1093/eurheartj/ehae603 39217475

[28] Havers-Borgersen E Butt JH Østergaard L Petersen JK Torp-Pedersen C Køber L Long-term incidence of infective endocarditis among patients with congenital heart disease. Am Heart J. (2023) 259:9–20. 10.1016/j.ahj.2023.01.012 36681172

[29] Noubiap JJ Nkeck JR Kwondom BS Nyaga UF. Epidemiology of infective endocarditis in Africa: a systematic review and meta-analysis. Lancet Glob Health. (2022) 10(1):e77–86. 10.1016/s2214-109x(21)00400-9 34919859

[30] Xiao J Yin L Lin Y Zhang Y Wu L Wang Z. A 20-year study on treating childhood infective endocarditis with valve replacement in a single cardiac center in China. J Thorac Dis. (2016) 8(7):1618–24. 10.21037/jtd.2016.06.15 27499950 PMC4958789

[31] Musci T Grubitzsch H. Healthcare-associated infective endocarditis-surgical perspectives. J Clin Med. (2022) 11(17):4957. 10.3390/jcm11174957 36078887 PMC9457102

[32] Mutagaywa RK Vroon JC Fundikira L Wind AM Kunambi P Manyahi J Infective endocarditis in developing countries: an update. Front Cardiovasc Med. (2022) 9:1007118. 10.3389/fcvm.2022.1007118 36172579 PMC9510687

[33] Li M Kim JB Sastry BKS Chen M. Infective endocarditis. Lancet. (2024) 404(10450):377–92. 10.1016/s0140-6736(24)01098-5 39067905

[34] Ahmed O King NE Qureshi MA Choudhry AA Osama M Zehner C Non-bacterial thrombotic endocarditis: a clinical and pathophysiological reappraisal. Eur Heart J. (2025) 46(3):236–49. 10.1093/eurheartj/ehae788 39565324

[35] Zmaili M Alzubi J Lo Presti Vega S Ababneh E Xu B. Non-bacterial thrombotic endocarditis: a state-of-the-art contemporary review. Prog Cardiovasc Dis. (2022) 74:99–110. 10.1016/j.pcad.2022.10.009 36279942

[36] Werdan K Dietz S Löffler B Niemann S Bushnaq H Silber RE Mechanisms of infective endocarditis: pathogen-host interaction and risk states. Nat Rev Cardiol. (2014) 11(1):35–50. 10.1038/nrcardio.2013.174 24247105

[37] Johannessen M Sollid JE Hanssen AM. Host- and microbe determinants that may influence the success of S. aureus colonization. Front Cell Infect Microbiol. (2012) 2:56. 10.3389/fcimb.2012.00056 22919647 PMC3417514

[38] Lerche CJ Schwartz F Theut M Fosbøl EL Iversen K Bundgaard H Anti-biofilm approach in infective endocarditis exposes new treatment strategies for improved outcome. Front Cell Dev Biol. (2021) 9:643335. 10.3389/fcell.2021.643335 34222225 PMC8249808

[39] Moser C Pedersen HT Lerche CJ Kolpen M Line L Thomsen K Biofilms and host response - helpful or harmful. APMIS. (2017) 125(4):320–38. 10.1111/apm.12674 28407429

[40] Slouha E Al-Geizi H Albalat BR Burle VS Clunes LA Kollias TF. Sex differences in infective endocarditis: a systematic review. Cureus. (2023) 15(12):e49815. 10.7759/cureus.49815 38169615 PMC10758535

[41] Stahl A Oestergaard L Havers-Borgersen E Emanuel Strange J Voldstedlund M Kober L Sex differences in characteristics, microbiology, management, and outcomes in infective endocarditis. Eur Heart J. (2023) 44(2):ehad655-1775. 10.1093/eurheartj/ehad655.1775

