# Supplementary material for: The global burden of pediatric infective endocarditis (5–14 years): epidemiological patterns from 1990 to 2021 and projected trajectories
Source: Front Cardiovasc Med. 2025 Oct 20;12:1657644. doi: 10.3389/fcvm.2025.1657644 (PMC12580122; doi:10.3389/fcvm.2025.1657644)
Supplement: Supplementary file 1 [file Table1.pdf]

Table S1. Incidence of Infective Endocarditis in Children Between 1990 and 2021 at the national level

location	1990		2021		1990-2021	
	Incident cases	Incidence rate	Incident cases	Incidence rate	Cases change	EAPC
Afghanistan	97.06(62.23,150.58)	3.74(2.40,5.81)	313.23(199.70,487.65)	3.59(2.29,5.59)	222.70(194.07,250.41)	-0.22(-0.30,-0.14)
Albania	15.97(9.67,25.79)	2.24(1.36,3.61)	7.78(4.77,12.18)	2.58(1.58,4.05)	-51.30(-54.84,-46.50)	0.55(0.45,0.65)
Algeria	276.76(173.82,429.68)	3.96(2.49,6.15)	348.09(223.07,536.73)	4.05(2.59,6.24)	25.77(13.61,36.67)	0.11(0.08,0.15)
American Samoa	0.55(0.35,0.84)	4.80(3.03,7.36)	0.59(0.39,0.90)	5.68(3.68,8.62)	8.71(-4.14,23.77)	0.69(0.60,0.78)
Andorra	0.17(0.10,0.28)	2.54(1.49,4.19)	0.23(0.14,0.37)	3.04(1.82,4.88)	34.96(25.01,50.86)	0.62(0.52,0.71)
Angola	116.39(74.17,176.76)	4.21(2.68,6.40)	420.19(272.18,644.23)	4.37(2.83,6.70)	261.00(226.22,292.90)	0.17(0.11,0.23)
Antigua and Barbuda	0.88(0.62,1.28)	7.22(5.08,10.54)	0.78(0.53,1.17)	6.75(4.52,10.09)	-10.43(-18.81,-0.59)	-0.31(-0.43,-0.20)
Argentina	245.58(157.32,375.07)	3.67(2.35,5.60)	412.73(260.35,641.47)	5.74(3.62,8.93)	68.07(47.87,91.25)	1.45(1.27,1.63)
Armenia	10.27(5.53,17.25)	1.55(0.84,2.61)	7.40(4.14,12.67)	1.82(1.02,3.12)	-27.90(-32.84,-20.65)	0.55(0.51,0.60)
Australia	62.34(35.72,101.31)	2.47(1.42,4.01)	91.87(54.25,147.12)	2.83(1.67,4.53)	47.37(35.42,60.09)	0.39(0.33,0.44)
Austria	31.57(22.35,43.56)	3.50(2.48,4.83)	37.74(26.44,53.28)	4.36(3.06,6.16)	19.54(5.24,34.35)	1.43(0.87,1.99)
Azerbaijan	25.14(13.63,43.22)	1.65(0.89,2.84)	30.17(16.70,50.57)	1.84(1.02,3.08)	20.00(10.92,32.47)	0.48(0.41,0.54)
Bahamas	3.61(2.45,5.27)	6.55(4.44,9.55)	3.81(2.51,5.74)	6.33(4.18,9.55)	5.45(-3.91,14.10)	-0.10(-0.21,0.01)
Bahrain	4.40(2.64,6.94)	4.32(2.59,6.81)	8.80(5.25,14.37)	4.33(2.59,7.08)	99.99(82.97,119.89)	0.04(-0.04,0.12)
Bangladesh	404.14(216.99,686.98)	1.35(0.72,2.29)	497.81(277.34,805.22)	1.59(0.88,2.56)	23.18(9.97,38.37)	0.60(0.56,0.64)
Barbados	3.22(2.25,4.64)	7.53(5.27,10.84)	2.56(1.76,3.79)	7.65(5.26,11.33)	-20.58(-27.65,-13.12)	0.04(-0.06,0.13)
Belarus	37.86(21.56,61.57)	2.37(1.35,3.85)	31.68(18.74,51.22)	2.85(1.69,4.61)	-16.33(-23.14,-8.14)	0.72(0.65,0.79)
Belgium	26.90(15.40,44.64)	2.22(1.27,3.69)	44.11(26.94,70.27)	3.34(2.04,5.32)	63.98(48.97,82.99)	1.05(0.69,1.40)
Belize	2.74(1.73,4.20)	5.23(3.31,8.03)	4.48(2.79,7.03)	5.27(3.28,8.28)	63.70(46.74,80.12)	-0.01(-0.13,0.12)
Benin	128.20(97.07,172.15)	8.93(6.76,12.00)	257.80(188.05,350.94)	6.90(5.03,9.39)	101.09(80.29,122.46)	-0.88(-0.97,-0.79)
Bermuda	0.50(0.33,0.75)	6.52(4.33,9.89)	0.37(0.23,0.58)	6.21(3.92,9.81)	-26.51(-33.94,-19.24)	-0.21(-0.23,-0.18)

location	1990		2021		1990-2021	
	Incident cases	Incidence rate	Incident cases	Incidence rate	Cases change	EAPC
Bhutan	2.29(1.21,3.95)	1.37(0.73,2.37)	1.93(1.07,3.14)	1.53(0.85,2.49)	-15.74(-24.82,-4.25)	0.44(0.37,0.51)
Bolivia (Plurinational State of)	57.34(39.27,83.49)	3.42(2.34,4.98)	77.16(51.40,115.99)	3.37(2.24,5.06)	34.56(20.81,46.38)	0.01(-0.05,0.06)
Bosnia and Herzegovina	17.36(10.13,28.95)	2.33(1.36,3.89)	8.72(5.05,14.62)	2.57(1.49,4.31)	-49.75(-54.68,-45.19)	0.36(0.30,0.43)
Botswana	17.10(10.39,27.47)	4.53(2.75,7.28)	19.64(12.06,31.06)	4.24(2.61,6.71)	14.87(1.76,31.09)	-0.21(-0.23,-0.18)
Brazil	1710.97(1075.28,2615.21)	4.82(3.03,7.37)	1452.67(933.82,2193.97)	4.59(2.95,6.94)	-15.10(-18.81,-10.80)	-0.16(-0.33,0.01)
Brunei Darussalam	1.87(1.20,2.87)	3.34(2.15,5.12)	2.48(1.66,3.70)	3.90(2.60,5.82)	32.71(22.22,45.79)	0.40(0.33,0.47)
Bulgaria	41.74(28.98,60.68)	3.48(2.42,5.06)	30.79(21.88,43.74)	4.57(3.25,6.49)	-26.25(-32.59,-20.20)	1.15(1.02,1.29)
Burkina Faso	226.19(164.82,309.33)	7.96(5.80,10.88)	414.57(308.64,561.47)	6.62(4.93,8.96)	83.28(65.93,100.64)	-0.61(-0.81,-0.41)
Burundi	72.24(47.47,107.51)	4.67(3.07,6.95)	171.54(114.66,257.25)	4.64(3.10,6.96)	137.46(119.59,154.45)	-0.02(-0.07,0.03)
Cabo Verde	9.53(7.00,13.09)	9.75(7.16,13.38)	8.37(6.01,11.59)	8.44(6.06,11.69)	-12.25(-22.95,-3.61)	-0.31(-0.38,-0.25)
Cambodia	86.93(51.42,142.63)	3.07(1.81,5.03)	98.61(58.15,158.64)	2.93(1.73,4.71)	13.44(3.26,23.76)	-0.15(-0.21,-0.08)
Cameroon	220.49(163.03,304.02)	7.67(5.67,10.57)	554.50(405.51,787.80)	6.45(4.71,9.16)	151.48(127.38,175.54)	-0.61(-0.75,-0.47)
Canada	103.36(61.18,167.84)	2.70(1.60,4.39)	117.58(69.52,187.94)	2.75(1.63,4.40)	13.76(5.09,22.14)	0.08(0.06,0.10)
Central African Republic	29.95(19.32,45.63)	4.17(2.69,6.35)	56.79(37.09,86.56)	3.93(2.57,5.99)	89.62(75.07,107.70)	-0.14(-0.22,-0.07)
Chad	130.64(96.19,177.62)	7.76(5.71,10.55)	344.84(258.89,476.10)	6.41(4.81,8.85)	163.97(137.36,190.08)	-0.62(-0.72,-0.52)
Chile	64.16(39.63,102.55)	2.53(1.56,4.05)	60.52(42.19,86.07)	2.36(1.65,3.36)	-5.67(-21.90,13.47)	-0.14(-0.37,0.10)
China	25676.43(17282.29,38474.52)	12.43(8.37,18.63)	8415.52(5459.88,12778.98)	4.62(3.00,7.02)	-67.22(-70.84,-63.78)	-3.88(-4.33,-3.44)
Colombia	313.25(211.88,464.79)	4.20(2.84,6.23)	336.12(226.67,497.01)	4.69(3.16,6.93)	7.30(-1.77,19.12)	0.29(0.17,0.41)
Comoros	6.50(4.19,9.77)	4.99(3.22,7.50)	7.85(5.23,11.65)	4.94(3.29,7.33)	20.79(11.49,30.76)	0.03(-0.03,0.09)
Congo	30.95(20.05,47.52)	4.70(3.05,7.22)	66.05(44.66,97.33)	5.09(3.44,7.51)	113.36(97.73,132.43)	0.37(0.32,0.43)
Cook Islands	0.26(0.18,0.38)	5.92(4.04,8.84)	0.15(0.10,0.22)	5.53(3.57,8.44)	-42.91(-49.97,-36.18)	-0.31(-0.38,-0.24)
Costa Rica	30.13(18.32,47.17)	4.20(2.55,6.57)	35.69(22.05,56.77)	5.03(3.11,8.01)	18.45(4.50,30.42)	0.63(0.55,0.70)
Croatia	17.66(10.68,28.14)	2.58(1.56,4.10)	10.01(6.52,14.83)	2.41(1.57,3.57)	-43.34(-50.34,-35.71)	-0.30(-0.51,-0.08)

location	1990		2021		1990-2021	
	Incident cases	Incidence rate	Incident cases	Incidence rate	Cases change	EAPC
Cuba	114.06(78.84,165.52)	7.09(4.90,10.29)	80.40(53.47,122.06)	6.52(4.34,9.90)	-29.51(-36.10,-21.86)	-0.49(-0.55,-0.42)
Cyprus	2.96(1.75,4.62)	2.21(1.30,3.45)	4.42(2.65,7.00)	3.08(1.84,4.87)	49.56(36.35,65.23)	1.17(1.04,1.30)
Czechia	47.16(28.81,74.95)	3.03(1.85,4.82)	36.07(21.96,58.51)	3.12(1.90,5.07)	-23.52(-29.97,-14.84)	0.25(0.18,0.32)
C 么 te d'Ivoire	308.71(233.87,410.80)	9.10(6.89,12.11)	521.60(385.26,708.45)	7.23(5.34,9.82)	68.96(50.49,86.32)	-0.79(-0.82,-0.76)
Democratic People's Republic of Korea	199.41(134.90,293.29)	5.52(3.73,8.12)	176.17(121.09,255.89)	5.40(3.71,7.85)	-11.65(-18.69,-3.44)	-0.11(-0.14,-0.08)
Democratic Republic of the Congo	454.91(292.12,690.34)	4.37(2.81,6.63)	1118.05(731.57,1672.43)	4.58(2.99,6.84)	145.78(127.67,167.03)	0.22(0.16,0.28)
Denmark	15.56(9.02,26.04)	2.62(1.52,4.39)	24.08(14.42,38.15)	3.74(2.24,5.93)	54.72(39.37,71.93)	1.13(0.99,1.27)
Djibouti	5.34(3.44,8.16)	4.88(3.15,7.45)	12.62(8.43,18.69)	4.72(3.15,6.99)	136.34(115.73,159.61)	-0.09(-0.14,-0.05)
Dominica	0.99(0.64,1.49)	6.13(3.98,9.23)	0.61(0.41,0.92)	5.99(4.01,9.00)	-38.29(-43.63,-31.14)	-0.17(-0.35,0.01)
Dominican Republic	84.92(55.95,122.98)	5.00(3.29,7.24)	103.50(69.70,152.06)	5.43(3.66,7.98)	21.88(11.34,32.78)	0.21(0.13,0.28)
Ecuador	81.46(56.02,118.33)	3.23(2.22,4.70)	111.00(77.73,157.80)	3.26(2.28,4.63)	36.26(23.76,49.76)	-0.06(-0.31,0.19)
Egypt	486.19(311.17,729.69)	3.56(2.28,5.35)	852.66(522.19,1335.32)	3.58(2.19,5.61)	75.38(56.78,92.57)	0.08(-0.01,0.18)
El Salvador	45.61(29.09,70.60)	3.29(2.10,5.09)	47.69(29.72,74.20)	3.92(2.44,6.09)	4.57(-6.78,13.83)	0.45(0.26,0.65)
Equatorial Guinea	4.97(3.17,7.59)	4.34(2.76,6.62)	21.08(13.91,31.68)	5.30(3.50,7.97)	323.76(290.73,365.12)	0.78(0.64,0.91)
Eritrea	46.36(30.60,68.74)	4.79(3.16,7.10)	70.58(45.90,105.94)	4.39(2.86,6.59)	52.26(38.13,67.15)	-0.30(-0.35,-0.25)
Estonia	5.66(3.34,9.15)	2.48(1.46,4.00)	4.18(2.47,6.72)	2.84(1.68,4.57)	-26.18(-31.13,-19.24)	0.55(0.49,0.61)
Eswatini	10.00(5.97,16.06)	4.16(2.48,6.68)	10.79(6.63,17.33)	3.96(2.44,6.37)	7.94(-2.75,18.81)	-0.17(-0.21,-0.12)
Ethiopia	620.26(397.02,957.23)	4.21(2.70,6.50)	1179.80(737.88,1824.29)	4.16(2.60,6.43)	90.21(82.79,100.62)	0.06(-0.09,0.20)
Fiji	10.42(7.14,15.62)	5.57(3.81,8.34)	11.60(8.11,16.97)	6.39(4.47,9.35)	11.27(-1.31,25.79)	0.57(0.50,0.64)
Finland	18.18(10.78,29.85)	2.79(1.65,4.58)	16.37(10.01,26.20)	2.71(1.66,4.34)	-9.96(-19.61,-3.27)	-0.07(-0.11,-0.02)
France	203.44(121.79,329.61)	2.60(1.56,4.21)	270.94(169.91,418.84)	3.36(2.10,5.19)	33.18(21.75,46.46)	0.87(0.79,0.95)
Gabon	11.97(7.85,18.28)	4.76(3.12,7.27)	22.11(14.64,33.11)	5.20(3.44,7.78)	84.70(70.42,100.03)	0.21(0.16,0.26)
Gambia	21.42(15.53,29.96)	7.77(5.63,10.87)	39.54(27.46,56.63)	6.22(4.32,8.91)	84.62(65.47,103.36)	-0.69(-0.75,-0.63)

location	1990		2021		1990-2021	
	Incident cases	Incidence rate	Incident cases	Incidence rate	Cases change	EAPC
Georgia	20.48(12.15,33.44)	2.27(1.35,3.72)	11.59(7.04,18.36)	2.35(1.43,3.73)	-43.37(-48.54,-35.49)	0.21(0.14,0.29)
Germany	413.65(290.28,612.37)	4.89(3.43,7.23)	540.28(389.11,761.93)	6.82(4.91,9.62)	30.61(19.61,45.75)	0.77(0.60,0.93)
Ghana	408.38(315.98,540.10)	10.00(7.74,13.23)	814.72(637.83,1055.58)	9.88(7.74,12.81)	99.50(80.18,120.64)	-0.14(-0.26,-0.02)
Greece	39.86(23.48,66.30)	2.71(1.60,4.51)	29.04(17.80,46.04)	2.99(1.83,4.74)	-27.15(-34.37,-20.79)	0.29(0.21,0.36)
Greenland	0.23(0.14,0.37)	2.66(1.61,4.23)	0.21(0.13,0.33)	2.76(1.71,4.27)	-8.20(-15.67,-0.76)	0.15(0.06,0.25)
Grenada	1.30(0.89,1.90)	6.08(4.18,8.89)	1.07(0.74,1.55)	7.13(4.96,10.33)	-17.99(-24.72,-9.98)	0.47(0.36,0.57)
Guam	1.18(0.76,1.83)	4.64(2.97,7.17)	1.72(1.24,2.49)	7.24(5.22,10.48)	45.37(25.88,71.86)	1.69(1.56,1.81)
Guatemala	76.86(48.55,116.95)	3.04(1.92,4.63)	113.56(69.92,179.26)	3.37(2.07,5.31)	47.75(30.40,62.69)	0.31(0.16,0.45)
Guinea	138.85(105.35,185.95)	8.73(6.62,11.69)	278.30(209.42,377.93)	7.33(5.52,9.96)	100.43(84.09,122.28)	-0.51(-0.61,-0.42)
Guinea-Bissau	23.66(17.64,31.88)	8.04(5.99,10.83)	36.07(26.00,50.47)	6.38(4.60,8.92)	52.43(35.09,67.50)	-0.78(-0.85,-0.71)
Guyana	10.90(7.50,15.47)	6.02(4.14,8.54)	9.61(6.89,13.73)	6.92(4.96,9.89)	-11.91(-20.86,-3.06)	0.26(0.18,0.34)
Haiti	85.05(58.94,123.95)	5.15(3.57,7.50)	137.21(93.29,196.81)	4.93(3.35,7.07)	61.34(46.99,76.44)	-0.05(-0.16,0.06)
Honduras	44.99(26.20,73.80)	3.25(1.89,5.32)	83.09(49.22,135.86)	3.81(2.26,6.23)	84.68(63.50,105.70)	0.56(0.42,0.71)
Hungary	38.39(22.28,63.36)	2.54(1.47,4.19)	24.21(14.37,39.90)	2.59(1.54,4.27)	-36.92(-42.72,-28.52)	-0.03(-0.06,-0.00)
Iceland	1.02(0.61,1.58)	2.40(1.44,3.74)	1.64(1.02,2.54)	3.61(2.23,5.58)	61.47(45.26,80.45)	1.32(1.16,1.47)
India	3212.19(1766.49,5339.79)	1.53(0.84,2.54)	4052.64(2238.73,6594.79)	1.59(0.88,2.59)	26.16(20.72,34.02)	0.15(0.04,0.26)
Indonesia	1731.41(1038.52,2783.89)	3.81(2.29,6.13)	1503.10(903.29,2377.84)	3.31(1.99,5.24)	-13.19(-16.43,-9.63)	-0.43(-0.60,-0.25)
Iran (Islamic Republic of)	891.13(580.20,1364.61)	5.36(3.49,8.21)	780.34(502.91,1184.11)	5.56(3.59,8.44)	-12.43(-15.16,-9.51)	0.30(0.21,0.40)
Iraq	219.35(141.28,335.35)	4.31(2.77,6.58)	435.19(279.05,662.34)	4.75(3.04,7.22)	98.40(81.16,117.34)	0.34(0.27,0.40)
Ireland	17.38(10.14,28.32)	2.51(1.46,4.08)	20.79(12.55,32.89)	2.97(1.80,4.71)	19.64(9.97,32.48)	0.47(0.37,0.57)
Israel	47.00(33.47,68.33)	4.62(3.29,6.72)	73.85(49.90,113.55)	4.32(2.92,6.64)	57.10(42.63,70.81)	-0.52(-0.76,-0.28)
Italy	449.80(273.10,701.28)	6.94(4.21,10.82)	164.45(107.59,244.80)	3.03(1.98,4.51)	-63.44(-68.00,-56.21)	-2.83(-3.51,-2.15)
Jamaica	28.93(18.44,45.18)	5.20(3.31,8.12)	26.34(16.84,40.08)	6.38(4.08,9.71)	-8.94(-16.60,1.32)	0.65(0.53,0.77)

location	1990		2021		1990-2021	
	Incident cases	Incidence rate	Incident cases	Incidence rate	Cases change	EAPC
Japan	824.24(495.07,1316.14)	5.02(3.01,8.01)	402.86(242.43,646.98)	3.71(2.23,5.96)	-51.12(-54.98,-48.32)	-1.10(-1.32,-0.89)
Jordan	45.37(28.19,70.82)	4.39(2.73,6.86)	106.40(64.23,166.84)	4.20(2.53,6.58)	134.54(109.90,156.86)	-0.11(-0.19,-0.02)
Kazakhstan	60.63(33.60,102.12)	1.83(1.01,3.08)	73.06(43.34,118.22)	2.10(1.25,3.40)	20.49(7.86,33.62)	0.59(0.52,0.65)
Kenya	417.44(262.52,642.16)	6.07(3.82,9.34)	676.03(430.79,1041.53)	5.32(3.39,8.19)	61.95(56.27,68.87)	-0.54(-0.72,-0.37)
Kiribati	1.04(0.72,1.50)	5.84(4.08,8.44)	1.65(1.20,2.27)	5.94(4.34,8.18)	58.70(43.02,78.81)	-0.00(-0.03,0.02)
Kuwait	16.60(10.63,25.72)	4.74(3.04,7.35)	27.49(17.60,43.22)	4.74(3.03,7.45)	65.58(50.40,82.10)	0.08(-0.01,0.17)
Kyrgyzstan	16.31(8.62,28.18)	1.58(0.83,2.72)	25.21(14.16,41.49)	1.70(0.96,2.80)	54.56(40.02,70.07)	0.30(0.25,0.36)
Lao People's Democratic Republic	36.35(21.78,58.26)	3.21(1.92,5.15)	44.37(26.70,71.23)	3.03(1.82,4.86)	22.06(10.62,36.62)	-0.21(-0.29,-0.13)
Latvia	9.73(5.69,15.98)	2.66(1.55,4.36)	6.31(3.73,10.11)	3.10(1.83,4.97)	-35.14(-40.75,-28.67)	0.34(-0.07,0.76)
Lebanon	31.08(20.84,46.66)	4.74(3.18,7.11)	41.89(27.06,63.84)	4.80(3.10,7.32)	34.79(24.47,47.61)	0.21(0.15,0.27)
Lesotho	17.81(10.84,28.14)	4.08(2.48,6.45)	15.54(9.56,25.26)	3.64(2.24,5.92)	-12.75(-20.25,-2.92)	-0.33(-0.39,-0.27)
Liberia	61.29(47.25,81.65)	9.21(7.10,12.26)	83.54(58.01,121.07)	5.88(4.09,8.53)	36.29(15.09,58.99)	-1.42(-1.52,-1.32)
Libya	66.31(48.65,92.27)	5.65(4.14,7.86)	48.57(33.31,69.94)	4.55(3.12,6.55)	-26.75(-34.93,-17.93)	-0.67(-0.71,-0.64)
Lithuania	12.82(7.50,21.01)	2.37(1.38,3.88)	7.46(4.33,12.00)	2.70(1.57,4.35)	-41.84(-47.89,-35.19)	0.90(0.50,1.31)
Luxembourg	1.31(0.83,2.02)	3.04(1.92,4.67)	2.44(1.61,3.73)	3.58(2.37,5.47)	85.47(70.15,101.43)	0.46(0.42,0.49)
Madagascar	173.52(117.44,262.20)	5.26(3.56,7.94)	398.77(271.97,578.96)	5.22(3.56,7.57)	129.81(111.25,151.19)	0.04(-0.02,0.09)
Malawi	121.28(78.72,183.82)	4.58(2.97,6.93)	252.08(161.22,387.23)	4.67(2.99,7.17)	107.85(91.37,133.42)	0.09(-0.05,0.22)
Malaysia	191.36(121.40,296.89)	4.57(2.90,7.08)	234.30(145.19,365.52)	4.54(2.82,7.09)	22.44(12.22,35.84)	0.07(0.02,0.12)
Maldives	2.42(1.56,3.67)	3.83(2.47,5.81)	2.63(1.70,3.93)	3.85(2.48,5.74)	8.83(-0.08,17.84)	0.04(-0.04,0.11)
Mali	196.13(146.30,263.40)	8.17(6.09,10.97)	445.28(330.15,609.84)	6.37(4.72,8.72)	127.03(106.18,147.68)	-0.76(-0.90,-0.61)
Malta	2.17(1.48,3.26)	3.67(2.49,5.50)	2.89(2.14,4.00)	6.88(5.09,9.53)	33.00(18.04,54.43)	2.18(2.04,2.33)
Marshall Islands	0.59(0.37,0.92)	4.10(2.57,6.33)	0.50(0.33,0.73)	4.23(2.77,6.22)	-16.04(-25.02,-4.95)	0.17(0.07,0.27)
Mauritania	43.93(31.61,61.94)	7.94(5.71,11.19)	83.93(59.22,118.89)	7.02(4.95,9.94)	91.06(73.49,109.68)	-0.53(-0.58,-0.49)

location	1990		2021		1990-2021	
	Incident cases	Incidence rate	Incident cases	Incidence rate	Cases change	EAPC
Mauritius	8.62(5.30,13.77)	3.84(2.36,6.13)	5.60(3.49,8.60)	3.91(2.44,6.01)	-35.08(-41.88,-28.47)	-0.03(-0.14,0.08)
Mexico	853.42(521.79,1329.86)	3.95(2.41,6.15)	935.14(584.37,1443.31)	4.21(2.63,6.50)	9.57(3.41,16.51)	0.19(0.06,0.33)
Micronesia (Federated States of)	1.24(0.79,1.92)	4.11(2.60,6.35)	0.91(0.59,1.38)	4.33(2.80,6.53)	-26.46(-34.33,-18.29)	0.21(0.16,0.26)
Monaco	0.09(0.06,0.13)	3.74(2.46,5.58)	0.13(0.08,0.20)	3.86(2.48,5.97)	47.54(33.26,60.94)	0.13(0.07,0.18)
Mongolia	9.25(5.10,16.09)	1.65(0.91,2.87)	13.21(7.47,22.01)	1.90(1.07,3.16)	42.87(27.82,55.87)	0.54(0.48,0.60)
Montenegro	2.50(1.44,4.06)	2.29(1.32,3.72)	1.95(1.13,3.27)	2.59(1.50,4.34)	-21.97(-28.01,-14.84)	0.50(0.43,0.57)
Morocco	252.96(163.00,396.13)	4.06(2.62,6.36)	272.82(173.00,421.63)	4.17(2.64,6.45)	7.85(0.67,16.59)	0.15(0.08,0.21)
Mozambique	183.68(117.97,281.58)	4.84(3.11,7.42)	432.85(280.12,677.54)	4.76(3.08,7.46)	135.65(117.43,160.13)	-0.11(-0.25,0.02)
Myanmar	301.08(180.55,479.32)	3.09(1.85,4.92)	326.83(196.45,511.32)	3.15(1.89,4.92)	8.55(-0.98,20.02)	0.15(0.04,0.26)
Namibia	16.52(10.06,26.23)	4.41(2.69,7.01)	23.11(14.12,36.37)	4.22(2.58,6.65)	39.84(27.16,55.45)	-0.16(-0.18,-0.13)
Nauru	0.12(0.08,0.18)	4.58(2.97,6.98)	0.13(0.09,0.19)	4.91(3.36,7.42)	6.85(-4.98,20.81)	0.20(0.14,0.25)
Nepal	69.88(38.16,119.85)	1.36(0.74,2.34)	91.67(50.43,153.26)	1.50(0.82,2.50)	31.18(18.70,46.93)	0.33(0.22,0.45)
Netherlands	43.95(25.93,70.23)	2.46(1.45,3.93)	60.43(38.43,93.65)	3.32(2.11,5.14)	37.49(24.91,52.40)	0.86(0.74,0.97)
New Zealand	22.38(12.99,36.45)	4.30(2.50,7.00)	19.46(11.62,30.58)	2.91(1.74,4.57)	-13.05(-20.39,-4.63)	-0.91(-1.52,-0.31)
Nicaragua	38.18(23.33,60.92)	3.30(2.02,5.26)	51.06(31.32,79.64)	3.84(2.36,5.99)	33.75(19.73,48.21)	0.55(0.46,0.64)
Niger	186.88(141.29,253.70)	7.87(5.95,10.68)	429.09(305.40,609.67)	5.60(3.98,7.95)	129.60(102.50,155.51)	-1.23(-1.44,-1.03)
Nigeria	2047.79(1487.79,2881.82)	8.82(6.40,12.41)	4199.86(2922.09,5983.38)	6.51(4.53,9.28)	105.09(91.82,117.03)	-1.04(-1.23,-0.86)
Niue	0.03(0.02,0.04)	5.00(3.27,7.65)	0.01(0.01,0.02)	5.52(3.70,8.21)	-46.08(-51.36,-40.18)	0.37(0.29,0.45)
North Macedonia	7.33(4.15,12.30)	2.06(1.17,3.45)	5.45(3.16,9.02)	2.40(1.39,3.97)	-25.64(-31.43,-18.65)	0.61(0.52,0.71)
Northern Mariana Islands	0.33(0.20,0.52)	4.44(2.68,7.06)	0.36(0.22,0.56)	4.44(2.71,6.89)	8.71(1.13,17.60)	-0.07(-0.15,0.00)
Norway	30.33(18.49,48.26)	5.80(3.54,9.24)	21.07(11.83,35.90)	3.28(1.84,5.59)	-30.53(-41.34,-20.38)	-2.14(-2.57,-1.72)
Oman	23.52(16.38,34.27)	4.59(3.20,6.68)	39.54(26.96,57.82)	4.95(3.37,7.24)	68.11(53.16,83.45)	0.40(0.33,0.47)
Pakistan	524.64(296.02,884.15)	1.70(0.96,2.87)	939.90(541.72,1500.17)	1.69(0.97,2.69)	79.15(67.63,93.08)	0.07(-0.00,0.14)

location	1990		2021		1990-2021	
	Incident cases	Incidence rate	Incident cases	Incidence rate	Cases change	EAPC
Palau	0.15(0.10,0.22)	4.73(3.09,7.25)	0.12(0.08,0.18)	5.14(3.38,7.75)	-18.36(-25.27,-10.68)	0.29(0.22,0.37)
Palestine	23.25(13.80,36.75)	4.02(2.39,6.36)	49.53(29.90,80.08)	3.95(2.38,6.38)	113.01(96.68,131.21)	-0.08(-0.16,0.00)
Panama	22.41(14.38,33.84)	4.09(2.62,6.17)	41.12(27.46,61.05)	5.26(3.51,7.81)	83.51(67.84,101.52)	0.80(0.78,0.82)
Papua New Guinea	38.38(23.53,61.24)	3.65(2.24,5.82)	83.13(51.08,129.01)	3.47(2.13,5.38)	116.62(99.03,137.24)	-0.16(-0.27,-0.04)
Paraguay	42.83(26.38,67.14)	4.07(2.51,6.38)	64.85(40.84,98.90)	4.78(3.01,7.28)	51.41(39.23,64.65)	0.60(0.47,0.73)
Peru	195.46(133.09,288.14)	3.64(2.48,5.36)	261.32(170.12,385.33)	4.19(2.73,6.18)	33.69(21.77,44.41)	0.55(0.49,0.61)
Philippines	598.64(350.47,973.98)	3.75(2.20,6.10)	793.04(488.73,1207.72)	3.48(2.14,5.30)	32.47(20.96,48.89)	-0.26(-0.40,-0.12)
Poland	211.56(130.56,337.94)	3.18(1.96,5.08)	119.12(71.37,187.60)	2.98(1.78,4.69)	-43.70(-47.33,-40.79)	-0.39(-0.44,-0.33)
Portugal	38.03(23.13,58.95)	2.47(1.50,3.83)	36.76(22.98,59.29)	3.92(2.45,6.33)	-3.34(-13.92,9.32)	1.53(1.33,1.73)
Puerto Rico	45.85(30.78,68.37)	6.78(4.55,10.11)	22.53(14.45,33.78)	6.64(4.26,9.96)	-50.86(-56.34,-44.90)	-0.16(-0.35,0.03)
Qatar	3.14(1.90,5.02)	4.23(2.56,6.76)	13.13(7.78,20.57)	4.24(2.51,6.64)	318.53(288.68,367.84)	0.04(-0.05,0.13)
Republic of Korea	314.67(212.87,470.22)	3.91(2.64,5.84)	147.63(93.12,222.20)	3.26(2.06,4.91)	-53.08(-59.19,-48.26)	-1.14(-1.38,-0.90)
Republic of Moldova	18.26(10.54,30.83)	2.27(1.31,3.83)	10.12(6.00,16.36)	2.75(1.63,4.44)	-44.58(-50.70,-38.28)	0.80(0.71,0.88)
Romania	86.58(48.24,147.52)	2.28(1.27,3.88)	57.51(33.61,92.95)	2.78(1.62,4.49)	-33.58(-39.50,-25.92)	0.43(0.35,0.50)
Russian Federation	710.34(427.79,1123.37)	3.08(1.85,4.87)	736.11(457.23,1125.55)	3.99(2.48,6.10)	3.63(-4.29,12.27)	0.93(0.84,1.01)
Rwanda	96.33(64.22,146.01)	4.71(3.14,7.14)	149.51(97.51,224.23)	4.64(3.03,6.96)	55.21(41.15,73.84)	-0.05(-0.10,-0.00)
Saint Kitts and Nevis	0.54(0.35,0.82)	5.68(3.67,8.67)	0.37(0.23,0.59)	5.51(3.45,8.69)	-30.12(-36.60,-22.36)	-0.20(-0.32,-0.09)
Saint Lucia	2.36(1.64,3.45)	6.96(4.83,10.18)	1.63(1.13,2.33)	7.80(5.41,11.18)	-30.99(-36.68,-24.55)	0.30(0.20,0.41)
Saint Vincent and the Grenadines	1.67(1.13,2.48)	5.89(4.01,8.75)	1.09(0.72,1.62)	6.11(4.07,9.14)	-34.97(-41.12,-28.41)	0.08(-0.01,0.18)
Samoa	2.01(1.30,3.10)	4.40(2.85,6.77)	2.33(1.50,3.53)	4.59(2.96,6.97)	15.66(2.89,27.48)	0.11(0.01,0.21)
San Marino	0.10(0.07,0.15)	3.45(2.32,5.30)	0.13(0.09,0.20)	4.07(2.72,6.19)	29.11(18.90,41.49)	0.48(0.44,0.52)
Sao Tome and Principe	3.32(2.48,4.54)	9.18(6.85,12.55)	4.07(2.92,5.72)	7.70(5.52,10.81)	22.58(8.88,36.79)	-0.41(-0.48,-0.34)
Saudi Arabia	170.07(101.15,276.64)	4.11(2.45,6.69)	216.03(130.24,343.82)	4.21(2.54,6.70)	27.03(17.91,37.21)	0.05(-0.05,0.15)

location	1990		2021		1990-2021	
	Incident cases	Incidence rate	Incident cases	Incidence rate	Cases change	EAPC
Senegal	169.15(125.78,234.90)	7.74(5.75,10.74)	286.08(208.73,392.91)	7.00(5.10,9.61)	69.13(53.35,87.67)	-0.42(-0.48,-0.36)
Serbia	26.22(14.81,42.50)	1.76(0.99,2.86)	23.59(13.44,38.41)	2.46(1.40,4.00)	-10.04(-18.01,2.24)	1.20(1.13,1.28)
Seychelles	0.70(0.44,1.08)	4.45(2.79,6.89)	0.64(0.41,0.99)	4.15(2.64,6.39)	-7.50(-14.83,0.69)	-0.22(-0.27,-0.18)
Sierra Leone	96.22(73.84,127.45)	9.26(7.11,12.27)	172.68(132.31,228.74)	7.73(5.92,10.24)	79.46(61.30,96.85)	-0.68(-0.84,-0.52)
Singapore	8.08(5.69,11.10)	1.81(1.28,2.49)	17.12(10.68,26.13)	3.25(2.03,4.97)	111.88(76.78,150.51)	1.53(1.20,1.86)
Slovakia	25.01(14.87,41.26)	2.73(1.62,4.50)	16.56(9.86,27.18)	2.90(1.73,4.76)	-33.80(-39.23,-28.72)	0.21(-0.05,0.47)
Slovenia	6.08(3.43,10.01)	2.09(1.18,3.45)	5.04(2.94,8.17)	2.35(1.37,3.81)	-17.03(-23.47,-5.70)	0.26(0.15,0.37)
Solomon Islands	3.82(2.45,5.83)	3.98(2.55,6.07)	6.32(4.11,9.57)	3.84(2.50,5.81)	65.38(50.15,84.19)	-0.12(-0.17,-0.06)
Somalia	105.66(68.21,160.53)	4.49(2.90,6.83)	250.35(163.48,378.60)	4.04(2.64,6.11)	136.94(119.03,156.11)	-0.34(-0.47,-0.20)
South Africa	413.86(251.46,655.89)	4.74(2.88,7.51)	406.49(245.81,640.03)	3.97(2.40,6.25)	-1.78(-7.76,2.96)	-0.58(-0.77,-0.40)
South Sudan	72.81(46.51,112.87)	4.53(2.90,7.03)	118.54(76.69,180.17)	4.34(2.81,6.59)	62.82(51.87,76.32)	-0.12(-0.25,-0.00)
Spain	142.66(85.29,233.06)	2.48(1.48,4.05)	116.06(79.02,165.98)	2.50(1.70,3.58)	-18.65(-31.98,-4.65)	-0.18(-0.39,0.04)
Sri Lanka	164.05(104.69,255.20)	4.36(2.78,6.78)	153.33(97.76,231.41)	4.33(2.76,6.54)	-6.54(-13.47,3.18)	0.05(-0.02,0.13)
Sudan	202.94(129.71,318.86)	3.75(2.40,5.89)	426.59(271.43,655.74)	3.90(2.48,5.99)	110.21(88.92,127.26)	0.14(0.06,0.23)
Suriname	4.24(2.75,6.37)	4.91(3.18,7.37)	4.93(3.16,7.62)	5.00(3.20,7.72)	16.31(7.27,27.00)	0.09(-0.02,0.20)
Sweden	34.14(19.71,54.64)	3.48(2.01,5.57)	47.67(28.70,75.97)	3.85(2.32,6.14)	39.63(30.14,50.56)	0.30(0.14,0.46)
Switzerland	18.70(11.02,30.80)	2.47(1.45,4.07)	22.44(13.24,36.29)	2.52(1.49,4.07)	19.97(11.93,27.80)	0.16(-0.18,0.50)
Syrian Arab Republic	148.77(91.52,239.31)	3.95(2.43,6.36)	101.05(61.43,160.76)	3.80(2.31,6.05)	-32.07(-38.40,-26.04)	-0.05(-0.21,0.11)
Taiwan (Province of China)	207.91(133.29,315.74)	5.33(3.42,8.09)	109.54(67.84,165.40)	5.33(3.30,8.05)	-47.31(-53.81,-40.73)	-0.16(-0.35,0.03)
Tajikistan	22.51(11.86,38.32)	1.64(0.86,2.78)	40.58(23.28,69.05)	1.81(1.04,3.07)	80.26(65.23,99.53)	0.41(0.35,0.48)
Thailand	705.23(503.00,996.23)	6.05(4.31,8.55)	404.66(284.06,581.26)	5.83(4.09,8.38)	-42.62(-47.55,-36.97)	-0.13(-0.18,-0.09)
Timor-Leste	6.55(3.89,10.73)	3.40(2.02,5.57)	10.84(6.45,17.25)	3.23(1.92,5.14)	65.46(51.00,83.23)	-0.09(-0.21,0.03)
Togo	86.88(64.28,119.98)	8.07(5.97,11.15)	135.93(98.61,196.75)	6.36(4.62,9.21)	56.47(41.10,72.16)	-0.75(-0.85,-0.64)

location	1990		2021		1990-2021	
	Incident cases	Incidence rate	Incident cases	Incidence rate	Cases change	EAPC
Tokelau	0.02(0.01,0.03)	4.33(2.73,6.66)	0.01(0.01,0.02)	4.55(2.88,7.00)	-24.51(-31.83,-14.16)	0.21(0.13,0.29)
Tonga	1.25(0.80,1.91)	4.70(3.02,7.19)	1.21(0.79,1.82)	4.91(3.20,7.39)	-3.07(-11.06,7.28)	0.19(0.11,0.27)
Trinidad and Tobago	13.25(8.18,21.09)	4.85(3.00,7.73)	10.16(6.23,16.38)	5.29(3.25,8.54)	-23.34(-28.97,-15.48)	0.40(0.24,0.56)
Tunisia	82.97(54.84,124.84)	4.07(2.69,6.12)	81.13(51.43,124.85)	4.33(2.74,6.66)	-2.22(-9.95,6.06)	0.31(0.23,0.39)
Turkey	14.91(8.19,26.03)	1.63(0.90,2.84)	18.54(10.15,30.90)	1.88(1.03,3.14)	24.32(12.88,36.36)	0.57(0.52,0.63)
Turkmenistan	0.08(0.05,0.13)	4.19(2.67,6.43)	0.10(0.07,0.16)	4.29(2.70,6.64)	27.19(15.76,42.21)	0.04(-0.07,0.16)
Tuvalu	517.91(322.30,803.88)	3.83(2.38,5.94)	491.20(296.80,778.31)	3.79(2.29,6.00)	-5.16(-12.70,2.43)	-0.16(-0.25,-0.08)
Uganda	245.78(158.52,378.96)	5.09(3.28,7.85)	608.31(400.06,920.70)	4.86(3.20,7.36)	147.50(123.09,174.58)	-0.21(-0.29,-0.13)
Ukraine	237.04(140.24,377.72)	3.12(1.84,4.97)	176.79(106.61,278.21)	3.72(2.24,5.85)	-25.42(-33.02,-16.33)	0.67(0.55,0.79)
United Arab Emirates	18.77(12.77,28.06)	5.19(3.53,7.76)	42.45(27.35,64.16)	4.69(3.02,7.08)	126.12(104.64,145.72)	-0.24(-0.28,-0.20)
United Kingdom	349.99(216.02,552.51)	4.94(3.05,7.80)	264.18(157.67,422.15)	3.25(1.94,5.19)	-24.52(-32.25,-17.57)	-0.70(-1.15,-0.25)
United Republic of Tanzania	339.28(213.10,517.80)	4.67(2.93,7.12)	750.11(494.86,1146.95)	4.82(3.18,7.37)	121.09(103.25,148.35)	0.12(0.03,0.20)
United States of America	2604.77(1646.82,4047.52)	7.20(4.55,11.19)	2302.67(1593.19,3388.00)	5.64(3.90,8.30)	-56.69(-60.60,-52.83)	-2.93(-3.97,-1.89)
United States Virgin Islands	1.39(0.94,2.06)	6.63(4.47,9.84)	0.60(0.39,0.92)	6.34(4.13,9.72)	-11.60(-21.99,2.34)	-0.26(-0.41,-0.11)
Uruguay	16.85(10.47,26.38)	3.09(1.92,4.83)	20.14(12.69,31.39)	4.33(2.73,6.74)	19.56(8.97,31.80)	1.09(0.96,1.23)
Uzbekistan	91.10(55.30,147.42)	1.76(1.07,2.84)	130.29(78.56,203.74)	2.08(1.26,3.26)	43.01(28.59,60.60)	0.63(0.57,0.68)
Vanuatu	1.86(1.15,2.97)	4.53(2.79,7.23)	3.31(2.09,5.06)	4.44(2.80,6.80)	77.79(62.25,97.19)	-0.01(-0.10,0.08)
Venezuela (Bolivarian Republic of)	164.54(101.27,265.40)	3.61(2.22,5.82)	177.33(106.02,286.11)	3.99(2.39,6.45)	7.77(-0.69,19.48)	0.30(0.17,0.42)
Viet Nam	715.84(461.56,1075.47)	4.19(2.70,6.29)	722.15(468.13,1086.69)	4.34(2.82,6.54)	0.88(-7.33,10.58)	0.22(0.12,0.31)
Yemen	160.81(101.34,254.71)	3.72(2.35,5.90)	334.35(212.31,521.96)	3.68(2.34,5.74)	107.91(90.08,125.09)	0.06(-0.05,0.16)
Zambia	94.52(58.84,145.84)	4.21(2.62,6.50)	236.01(154.34,356.64)	4.41(2.89,6.67)	149.70(131.55,172.84)	0.22(0.16,0.28)
Zimbabwe	134.75(82.84,211.08)	4.41(2.71,6.91)	162.81(102.79,251.16)	3.99(2.52,6.15)	20.83(10.03,35.24)	-0.42(-0.52,-0.33)
