# Supplementary material for: The global burden of pediatric infective endocarditis (5–14 years): epidemiological patterns from 1990 to 2021 and projected trajectories
Source: Front Cardiovasc Med. 2025 Oct 20;12:1657644. doi: 10.3389/fcvm.2025.1657644 (PMC12580122; doi:10.3389/fcvm.2025.1657644)
Supplement: Supplementary file 2 [file Table2.pdf]

Table S2. Mortality of infective endocarditis in children between 1990 and 2021 at the national level

location	1990		2021		1990-2021	
	Death cases	Death rate	Death cases	Death rate	Cases change	EAPC
Afghanistan	1.98(1.11,3.25)	0.08(0.04,0.13)	5.90(3.59,9.01)	0.07(0.04,0.10)	-82.40(-90.95,-59.00)	-0.28(-0.57,0.01)
Albania	0.35(0.19,0.53)	0.05(0.03,0.07)	0.06(0.03,0.10)	0.02(0.01,0.03)	-42.17(-77.25,17.49)	-1.22(-2.34,-0.08)
Algeria	6.63(3.88,10.88)	0.09(0.06,0.16)	3.64(2.33,5.67)	0.04(0.03,0.07)	49.66(22.27,80.12)	-2.60(-2.74,-2.46)
American Samoa	0.01(0.00,0.02)	0.07(0.03,0.13)	0.02(0.01,0.03)	0.16(0.09,0.26)	-7.12(-59.63,73.84)	4.97(4.11,5.83)
Andorra	0.00(0.00,0.00)	0.02(0.01,0.03)	0.00(0.00,0.00)	0.01(0.00,0.01)	-10.98(-42.87,38.85)	-2.26(-2.44,-2.07)
Angola	2.51(0.83,4.64)	0.09(0.03,0.17)	5.98(2.70,10.38)	0.06(0.03,0.11)	16.86(-46.53,120.57)	-1.16(-1.40,-0.91)
Antigua and Barbuda	0.01(0.00,0.01)	0.05(0.04,0.07)	0.01(0.00,0.01)	0.05(0.04,0.06)	-30.33(-58.44,17.27)	-0.42(-1.32,0.50)
Argentina	3.84(3.27,4.51)	0.06(0.05,0.07)	1.95(1.64,2.30)	0.03(0.02,0.03)	-37.33(-75.41,40.43)	-2.17(-2.42,-1.93)
Armenia	0.06(0.04,0.07)	0.01(0.01,0.01)	0.02(0.02,0.02)	0.01(0.00,0.01)	-14.16(-59.90,64.98)	-0.08(-0.74,0.58)
Australia	0.22(0.18,0.26)	0.01(0.01,0.01)	0.34(0.27,0.44)	0.01(0.01,0.01)	29.83(-3.81,88.37)	0.45(-0.19,1.09)
Austria	0.03(0.03,0.04)	0.00(0.00,0.00)	0.05(0.04,0.06)	0.01(0.00,0.01)	-46.19(-69.71,-11.77)	3.36(2.38,4.35)
Azerbaijan	0.08(0.04,0.12)	0.01(0.00,0.01)	0.07(0.04,0.10)	0.00(0.00,0.01)	-71.44(-82.97,-50.97)	-1.24(-1.95,-0.53)
Bahamas	0.06(0.05,0.07)	0.11(0.09,0.13)	0.04(0.03,0.05)	0.07(0.05,0.09)	114.86(-1.25,288.67)	-2.29(-2.96,-1.61)
Bahrain	0.05(0.04,0.08)	0.05(0.03,0.08)	0.07(0.05,0.10)	0.03(0.02,0.05)	-12.14(-57.90,46.49)	-1.25(-1.57,-0.93)
Bangladesh	13.20(5.81,25.87)	0.04(0.02,0.09)	14.34(8.17,22.90)	0.05(0.03,0.07)	52.17(-30.36,243.14)	0.56(0.37,0.75)
Barbados	0.05(0.04,0.06)	0.11(0.09,0.14)	0.02(0.01,0.03)	0.06(0.04,0.08)	121.30(10.11,304.90)	-0.48(-1.13,0.17)
Belarus	0.65(0.37,1.12)	0.04(0.02,0.07)	0.24(0.19,0.29)	0.02(0.02,0.03)	77.63(3.59,295.23)	-1.88(-2.23,-1.53)
Belgium	0.19(0.16,0.22)	0.02(0.01,0.02)	0.45(0.37,0.52)	0.03(0.03,0.04)	-4.38(-44.21,99.72)	2.73(1.88,3.58)
Belize	0.02(0.01,0.02)	0.03(0.03,0.04)	0.02(0.01,0.02)	0.02(0.01,0.02)	284.64(98.31,640.84)	-1.40(-1.91,-0.88)
Benin	0.96(0.46,1.64)	0.07(0.03,0.11)	2.80(1.41,4.67)	0.07(0.04,0.12)	37.49(-21.04,173.47)	0.43(0.33,0.52)
Bermuda	0.00(0.00,0.00)	0.02(0.02,0.03)	0.00(0.00,0.00)	0.02(0.01,0.03)	173.54(47.46,420.65)	-0.24(-0.83,0.36)

location	1990		2021		1990-2021	
	Death cases	Death rate	Death cases	Death rate	Cases change	EAPC
Bhutan	0.05(0.01,0.10)	0.03(0.01,0.06)	0.05(0.02,0.07)	0.04(0.02,0.06)	27.91(-31.90,187.50)	0.48(-0.10,1.07)
Bolivia (Plurinational State of)	3.42(2.02,5.32)	0.20(0.12,0.32)	2.67(1.81,3.88)	0.12(0.08,0.17)	188.00(58.28,493.80)	-1.74(-1.87,-1.60)
Bosnia and Herzegovina	0.05(0.03,0.08)	0.01(0.00,0.01)	0.01(0.01,0.02)	0.00(0.00,0.01)	-62.68(-72.10,-49.38)	-1.38(-1.66,-1.09)
Botswana	0.59(0.30,1.01)	0.16(0.08,0.27)	0.61(0.35,0.92)	0.13(0.07,0.20)	-17.64(-56.35,49.67)	-0.29(-0.50,-0.08)
Brazil	44.16(39.61,48.54)	0.12(0.11,0.14)	22.56(18.85,25.70)	0.07(0.06,0.08)	-68.74(-80.27,-49.29)	-1.08(-1.63,-0.54)
Brunei Darussalam	0.04(0.02,0.06)	0.07(0.04,0.11)	0.02(0.01,0.03)	0.03(0.01,0.04)	-43.22(-67.49,-5.07)	-1.98(-2.38,-1.58)
Bulgaria	0.28(0.23,0.34)	0.02(0.02,0.03)	0.21(0.16,0.26)	0.03(0.02,0.04)	-3.65(-26.73,38.94)	1.30(0.59,2.02)
Burkina Faso	2.05(0.93,3.74)	0.07(0.03,0.13)	5.51(2.89,8.87)	0.09(0.05,0.14)	-10.27(-49.21,77.76)	1.01(0.85,1.18)
Burundi	4.81(2.10,8.34)	0.31(0.14,0.54)	6.25(3.33,10.42)	0.17(0.09,0.28)	60.37(-38.67,200.56)	-1.87(-1.97,-1.77)
Cabo Verde	0.07(0.04,0.13)	0.08(0.04,0.13)	0.08(0.05,0.13)	0.08(0.05,0.13)	37.99(-2.27,105.05)	-0.59(-0.96,-0.22)
Cambodia	1.67(0.99,2.72)	0.06(0.03,0.10)	1.55(0.81,2.76)	0.05(0.02,0.08)	-29.88(-50.92,0.10)	-0.91(-1.04,-0.78)
Cameroon	2.37(1.03,4.21)	0.08(0.04,0.15)	7.89(4.04,12.74)	0.09(0.05,0.15)	-82.17(-89.74,-66.22)	0.57(0.20,0.94)
Canada	0.61(0.45,0.83)	0.02(0.01,0.02)	0.40(0.33,0.47)	0.01(0.01,0.01)	-72.31(-82.81,-50.14)	-2.00(-2.19,-1.81)
Central African Republic	0.76(0.28,1.38)	0.11(0.04,0.19)	1.37(0.55,2.53)	0.09(0.04,0.18)	-25.08(-45.52,2.92)	-0.40(-0.51,-0.28)
Chad	1.51(0.66,2.68)	0.09(0.04,0.16)	6.30(3.12,10.62)	0.12(0.06,0.20)	-51.85(-62.91,-41.07)	1.12(0.94,1.30)
Chile	0.36(0.30,0.43)	0.01(0.01,0.02)	0.33(0.27,0.41)	0.01(0.01,0.02)	-21.59(-40.91,1.46)	0.32(-0.36,1.01)
China	135.98(68.04,186.43)	0.07(0.03,0.09)	23.94(16.31,38.34)	0.01(0.01,0.02)	-56.10(-65.27,-44.61)	-6.22(-6.69,-5.76)
Colombia	8.01(7.01,9.28)	0.11(0.09,0.12)	8.40(6.86,10.17)	0.12(0.10,0.14)	-57.97(-75.65,-24.42)	0.82(0.28,1.36)
Comoros	0.32(0.11,0.55)	0.24(0.08,0.42)	0.24(0.14,0.38)	0.15(0.09,0.24)	-74.72(-86.31,-59.06)	-1.93(-2.47,-1.39)
Congo	0.47(0.18,0.82)	0.07(0.03,0.12)	0.76(0.37,1.31)	0.06(0.03,0.10)	-49.42(-55.45,-44.02)	-0.65(-0.95,-0.34)
Cook Islands	0.01(0.01,0.02)	0.26(0.15,0.43)	0.01(0.00,0.01)	0.21(0.11,0.34)	-56.59(-67.34,-40.02)	-1.30(-1.60,-1.00)
Costa Rica	0.54(0.46,0.63)	0.08(0.06,0.09)	0.54(0.46,0.64)	0.08(0.06,0.09)	-82.77(-90.70,-67.32)	0.44(0.14,0.75)
Croatia	0.07(0.06,0.08)	0.01(0.01,0.01)	0.03(0.03,0.04)	0.01(0.01,0.01)	-62.31(-77.65,-27.84)	0.03(-0.47,0.53)

location	1990		2021		1990-2021	
	Death cases	Death rate	Death cases	Death rate	Cases change	EAPC
Cuba	0.54(0.42,0.67)	0.03(0.03,0.04)	0.33(0.27,0.41)	0.03(0.02,0.03)	-55.38(-67.41,-41.02)	-0.15(-0.62,0.32)
Cyprus	0.04(0.02,0.06)	0.03(0.02,0.04)	0.02(0.01,0.03)	0.01(0.01,0.02)	-63.63(-80.20,-33.17)	-1.49(-2.32,-0.65)
Czechia	0.30(0.25,0.35)	0.02(0.02,0.02)	0.24(0.19,0.29)	0.02(0.02,0.03)	-75.95(-81.37,-69.28)	0.06(-0.42,0.55)
C 么 te d'Ivoire	2.86(1.25,4.91)	0.08(0.04,0.14)	7.30(3.51,12.18)	0.10(0.05,0.17)	-82.59(-86.82,-76.57)	0.89(0.69,1.09)
Democratic People's Republic of Korea	2.60(1.49,3.96)	0.07(0.04,0.11)	1.50(0.67,3.06)	0.05(0.02,0.09)	-55.94(-65.40,-42.68)	-1.37(-1.54,-1.20)
Democratic Republic of the Congo	9.71(3.86,17.96)	0.09(0.04,0.17)	18.34(8.77,34.14)	0.08(0.04,0.14)	-41.37(-54.08,-24.32)	-0.32(-0.50,-0.15)
Denmark	0.06(0.04,0.07)	0.01(0.01,0.01)	0.10(0.09,0.13)	0.02(0.01,0.02)	19.76(10.83,28.03)	1.76(0.95,2.57)
Djibouti	0.21(0.10,0.36)	0.19(0.09,0.33)	0.33(0.19,0.53)	0.12(0.07,0.20)	-26.73(-58.17,31.43)	-1.64(-1.93,-1.36)
Dominica	0.01(0.01,0.02)	0.08(0.05,0.11)	0.01(0.01,0.01)	0.09(0.06,0.13)	-56.65(-80.86,-13.79)	0.29(0.09,0.49)
Dominican Republic	1.47(1.01,2.06)	0.09(0.06,0.12)	1.70(1.04,2.59)	0.09(0.05,0.14)	-32.81(-36.55,-28.68)	-0.39(-0.66,-0.11)
Ecuador	1.53(1.31,1.79)	0.06(0.05,0.07)	2.57(2.13,3.11)	0.08(0.06,0.09)	-85.20(-92.83,-64.04)	1.10(0.20,2.00)
Egypt	27.10(15.88,43.44)	0.20(0.12,0.32)	16.16(10.07,25.07)	0.07(0.04,0.11)	-47.26(-58.77,-32.86)	-3.45(-3.56,-3.34)
El Salvador	0.55(0.37,0.78)	0.04(0.03,0.06)	0.33(0.22,0.47)	0.03(0.02,0.04)	57.23(18.49,109.83)	-0.72(-1.14,-0.29)
Equatorial Guinea	0.11(0.04,0.20)	0.10(0.04,0.18)	0.18(0.06,0.52)	0.05(0.02,0.13)	-4.98(-26.26,26.29)	-3.32(-3.65,-2.98)
Eritrea	2.13(0.95,3.69)	0.22(0.10,0.38)	2.32(1.30,3.72)	0.14(0.08,0.23)	-45.19(-72.00,19.25)	-1.28(-1.37,-1.19)
Estonia	0.12(0.10,0.13)	0.05(0.04,0.06)	0.03(0.02,0.03)	0.02(0.02,0.02)	74.28(36.28,119.80)	-2.25(-2.73,-1.77)
Eswatini	0.40(0.18,0.68)	0.17(0.08,0.28)	0.47(0.25,0.75)	0.17(0.09,0.28)	139.17(88.11,203.92)	0.37(0.25,0.50)
Ethiopia	26.51(10.66,44.76)	0.18(0.07,0.30)	34.60(19.97,57.53)	0.12(0.07,0.20)	-49.29(-69.32,-15.42)	-1.58(-1.71,-1.45)
Fiji	0.29(0.17,0.49)	0.16(0.09,0.26)	0.45(0.25,0.71)	0.25(0.14,0.39)	84.88(37.50,152.96)	2.00(1.71,2.28)
Finland	0.02(0.02,0.03)	0.00(0.00,0.00)	0.05(0.04,0.05)	0.01(0.01,0.01)	91.31(43.37,157.93)	2.41(1.46,3.38)
France	1.68(1.32,2.12)	0.02(0.02,0.03)	1.71(1.43,2.02)	0.02(0.02,0.03)	2.08(-23.66,43.83)	-0.08(-0.26,0.10)
Gabon	0.14(0.06,0.23)	0.06(0.02,0.09)	0.18(0.08,0.33)	0.04(0.02,0.08)	52.03(17.11,103.63)	-0.81(-0.97,-0.66)
Gambia	0.24(0.11,0.42)	0.09(0.04,0.15)	0.64(0.36,1.05)	0.10(0.06,0.16)	-53.23(-69.88,-27.45)	0.40(0.10,0.70)

location	1990		2021		1990-2021	
	Death cases	Death rate	Death cases	Death rate	Cases change	EAPC
Georgia	0.14(0.09,0.20)	0.02(0.01,0.02)	0.04(0.04,0.05)	0.01(0.01,0.01)	45.27(13.09,89.59)	-1.20(-3.26,0.91)
Germany	0.88(0.70,1.10)	0.01(0.01,0.01)	1.34(1.12,1.56)	0.02(0.01,0.02)	39.24(7.87,78.57)	2.70(1.80,3.61)
Ghana	3.28(1.65,5.24)	0.08(0.04,0.13)	4.05(2.50,6.16)	0.05(0.03,0.07)	178.56(114.88,261.64)	-1.58(-1.78,-1.38)
Greece	0.23(0.16,0.36)	0.02(0.01,0.02)	0.11(0.09,0.14)	0.01(0.01,0.01)	-16.35(-38.21,10.09)	-0.70(-1.06,-0.34)
Greenland	0.00(0.00,0.00)	0.00(0.00,0.01)	0.00(0.00,0.00)	0.00(0.00,0.01)	58.50(23.16,106.03)	0.17(-0.08,0.42)
Grenada	0.04(0.03,0.05)	0.17(0.14,0.21)	0.02(0.01,0.02)	0.10(0.08,0.12)	341.11(223.09,518.80)	-1.45(-1.83,-1.07)
Guam	0.02(0.01,0.04)	0.08(0.04,0.16)	0.02(0.01,0.04)	0.09(0.03,0.18)	97.56(56.81,145.81)	3.70(2.51,4.91)
Guatemala	1.89(1.31,2.70)	0.07(0.05,0.11)	2.25(1.79,2.74)	0.07(0.05,0.08)	-15.54(-26.07,-4.77)	-0.05(-0.31,0.21)
Guinea	1.55(0.66,2.76)	0.10(0.04,0.17)	4.17(1.95,7.13)	0.11(0.05,0.19)	-18.73(-33.37,-0.65)	0.71(0.52,0.89)
Guinea-Bissau	0.34(0.14,0.60)	0.12(0.05,0.20)	0.60(0.31,1.26)	0.11(0.05,0.22)	43.70(17.53,78.50)	-0.12(-0.40,0.17)
Guyana	0.11(0.09,0.14)	0.06(0.05,0.08)	0.25(0.19,0.32)	0.18(0.13,0.23)	-35.12(-51.72,-8.74)	4.20(2.82,5.59)
Haiti	3.76(2.10,6.34)	0.23(0.13,0.38)	6.69(3.83,10.61)	0.24(0.14,0.38)	53.19(24.47,95.61)	0.49(0.39,0.59)
Honduras	1.16(0.75,1.81)	0.08(0.05,0.13)	0.97(0.49,1.69)	0.04(0.02,0.08)	68.77(47.62,86.26)	-2.04(-2.26,-1.81)
Hungary	0.39(0.33,0.44)	0.03(0.02,0.03)	0.17(0.14,0.20)	0.02(0.01,0.02)	-49.17(-60.25,-36.41)	0.41(-0.24,1.07)
Iceland	0.00(0.00,0.00)	0.01(0.01,0.01)	0.00(0.00,0.01)	0.01(0.01,0.01)	-8.71(-30.20,19.11)	2.17(0.79,3.57)
India	89.59(47.37,136.79)	0.04(0.02,0.07)	72.83(48.34,95.49)	0.03(0.02,0.04)	-52.53(-63.43,-37.76)	-1.26(-1.56,-0.95)
Indonesia	32.56(21.21,49.24)	0.07(0.05,0.11)	28.99(17.87,48.65)	0.06(0.04,0.11)	-34.96(-53.40,-8.06)	-0.31(-0.48,-0.14)
Iran (Islamic Republic of)	11.08(6.96,14.26)	0.07(0.04,0.09)	4.69(2.98,6.38)	0.03(0.02,0.05)	-19.31(-34.65,-3.59)	-1.69(-2.18,-1.21)
Iraq	3.01(1.69,4.65)	0.06(0.03,0.09)	2.77(1.76,4.05)	0.03(0.02,0.04)	-5.10(-29.25,27.05)	-1.98(-2.38,-1.58)
Ireland	0.04(0.03,0.04)	0.01(0.00,0.01)	0.05(0.04,0.07)	0.01(0.01,0.01)	-30.65(-49.40,-4.77)	1.89(0.73,3.06)
Israel	0.08(0.06,0.09)	0.01(0.01,0.01)	0.21(0.17,0.26)	0.01(0.01,0.01)	-56.99(-71.54,-35.80)	2.37(1.25,3.49)
Italy	1.92(1.53,2.52)	0.03(0.02,0.04)	1.60(1.42,1.76)	0.03(0.03,0.03)	-11.69(-36.10,14.58)	-0.02(-0.38,0.34)
Jamaica	0.92(0.72,1.15)	0.16(0.13,0.21)	0.35(0.28,0.44)	0.09(0.07,0.11)	-38.43(-55.15,-11.13)	-2.19(-2.58,-1.80)

location	1990		2021		1990-2021	
	Death cases	Death rate	Death cases	Death rate	Cases change	EAPC
Japan	2.16(2.09,2.24)	0.01(0.01,0.01)	1.45(1.37,1.53)	0.01(0.01,0.01)	-29.62(-58.33,30.00)	-1.08(-1.93,-0.23)
Jordan	2.35(1.56,3.36)	0.23(0.15,0.32)	2.71(1.91,3.66)	0.11(0.08,0.14)	15.27(-29.62,104.85)	-2.87(-3.22,-2.52)
Kazakhstan	0.77(0.57,1.14)	0.02(0.02,0.03)	0.44(0.30,0.58)	0.01(0.01,0.02)	-59.46(-70.56,-42.68)	-3.06(-3.76,-2.35)
Kenya	6.59(3.33,9.73)	0.10(0.05,0.14)	9.93(6.16,13.84)	0.08(0.05,0.11)	113.55(47.16,199.98)	0.19(-0.15,0.53)
Kiribati	0.02(0.01,0.04)	0.13(0.08,0.21)	0.05(0.03,0.09)	0.19(0.11,0.32)	77.91(7.15,210.49)	1.01(0.85,1.18)
Kuwait	0.18(0.15,0.22)	0.05(0.04,0.06)	0.23(0.19,0.29)	0.04(0.03,0.05)	-61.58(-72.40,-46.76)	0.74(-0.63,2.14)
Kyrgyzstan	0.13(0.10,0.16)	0.01(0.01,0.02)	0.13(0.11,0.15)	0.01(0.01,0.01)	-13.75(-42.13,19.80)	-0.99(-1.42,-0.56)
Lao People's Democratic Republic	1.27(0.70,2.13)	0.11(0.06,0.19)	1.49(0.73,2.55)	0.10(0.05,0.17)	20.07(-7.09,56.67)	-0.20(-0.34,-0.07)
Latvia	0.10(0.08,0.13)	0.03(0.02,0.04)	0.02(0.01,0.02)	0.01(0.01,0.01)	4.73(-33.72,91.29)	-1.62(-2.29,-0.95)
Lebanon	0.40(0.25,0.60)	0.06(0.04,0.09)	0.31(0.21,0.44)	0.04(0.02,0.05)	-54.14(-68.27,-31.64)	-1.63(-1.76,-1.51)
Lesotho	0.59(0.26,1.11)	0.14(0.06,0.25)	0.84(0.37,1.43)	0.20(0.09,0.34)	-21.73(-54.83,40.76)	1.80(1.57,2.04)
Liberia	0.68(0.30,1.13)	0.10(0.04,0.17)	1.65(0.76,2.91)	0.12(0.05,0.21)	68.16(33.42,120.30)	0.36(0.12,0.60)
Libya	1.07(0.71,1.51)	0.09(0.06,0.13)	0.99(0.63,1.49)	0.09(0.06,0.14)	-26.40(-59.83,23.29)	0.32(0.14,0.51)
Lithuania	0.10(0.09,0.12)	0.02(0.02,0.02)	0.05(0.04,0.05)	0.02(0.01,0.02)	4.89(-17.31,31.79)	0.22(-0.34,0.78)
Luxembourg	0.01(0.01,0.01)	0.02(0.01,0.02)	0.01(0.01,0.01)	0.02(0.01,0.02)	-0.58(-20.32,27.35)	-0.66(-1.46,0.15)
Madagascar	9.38(4.18,15.59)	0.28(0.13,0.47)	20.44(11.90,32.29)	0.27(0.16,0.42)	-40.44(-64.05,-1.03)	-0.24(-0.40,-0.08)
Malawi	4.69(1.95,7.95)	0.18(0.07,0.30)	7.00(3.42,11.98)	0.13(0.06,0.22)	18.97(-23.82,80.64)	-0.83(-1.01,-0.65)
Malaysia	5.01(3.28,7.24)	0.12(0.08,0.17)	3.49(2.39,4.88)	0.07(0.05,0.09)	-15.73(-61.03,59.70)	-1.55(-1.85,-1.26)
Maldives	0.04(0.02,0.06)	0.06(0.03,0.10)	0.02(0.01,0.04)	0.03(0.02,0.05)	-11.35(-22.82,1.44)	-1.96(-2.35,-1.57)
Mali	1.94(0.80,3.33)	0.08(0.03,0.14)	5.74(2.94,10.07)	0.08(0.04,0.14)	-44.22(-63.43,-11.04)	0.22(0.02,0.42)
Malta	0.01(0.00,0.01)	0.01(0.01,0.01)	0.03(0.02,0.04)	0.07(0.05,0.09)	39.41(1.43,80.59)	4.65(3.47,5.83)
Marshall Islands	0.01(0.01,0.03)	0.09(0.05,0.18)	0.02(0.01,0.04)	0.21(0.12,0.34)	53.84(13.71,109.96)	2.41(2.11,2.71)
Mauritania	0.42(0.20,0.70)	0.08(0.04,0.13)	0.76(0.40,1.25)	0.06(0.03,0.10)	-48.92(-57.08,-40.83)	-0.70(-0.92,-0.49)

location	1990		2021		1990-2021	
	Death cases	Death rate	Death cases	Death rate	Cases change	EAPC
Mauritius	0.02(0.02,0.03)	0.01(0.01,0.01)	0.06(0.05,0.07)	0.04(0.03,0.05)	-3.09(-36.02,42.66)	8.91(5.82,12.09)
Mexico	13.61(12.69,14.60)	0.06(0.06,0.07)	12.06(10.80,13.50)	0.05(0.05,0.06)	-45.07(-70.19,-4.85)	0.78(-0.07,1.64)
Micronesia (Federated States of)	0.04(0.02,0.07)	0.13(0.07,0.23)	0.04(0.02,0.06)	0.18(0.11,0.29)	25.00(-19.49,115.38)	1.22(1.07,1.37)
Monaco	0.00(0.00,0.00)	0.04(0.02,0.06)	0.00(0.00,0.00)	0.03(0.02,0.04)	-40.36(-69.36,4.66)	-1.96(-2.47,-1.45)
Mongolia	0.13(0.07,0.20)	0.02(0.01,0.04)	0.11(0.08,0.16)	0.02(0.01,0.02)	-57.71(-70.35,-39.67)	-1.14(-1.76,-0.52)
Montenegro	0.01(0.01,0.01)	0.01(0.01,0.01)	0.00(0.00,0.00)	0.00(0.00,0.01)	-7.98(-52.92,58.19)	-2.83(-3.50,-2.15)
Morocco	7.48(4.55,11.71)	0.12(0.07,0.19)	3.19(1.91,5.31)	0.05(0.03,0.08)	15.26(-23.65,79.81)	-2.47(-2.78,-2.15)
Mozambique	4.76(1.97,8.18)	0.13(0.05,0.22)	9.71(4.16,16.95)	0.11(0.05,0.19)	28.57(-2.70,80.05)	-0.27(-0.48,-0.06)
Myanmar	12.17(6.98,20.28)	0.13(0.07,0.21)	10.45(6.12,17.84)	0.10(0.06,0.17)	-22.32(-53.82,33.29)	-0.86(-1.10,-0.61)
Namibia	0.51(0.21,0.90)	0.14(0.05,0.24)	0.70(0.38,1.17)	0.13(0.07,0.21)	-7.88(-45.75,59.58)	-0.09(-0.21,0.04)
Nauru	0.00(0.00,0.01)	0.15(0.07,0.26)	0.01(0.00,0.01)	0.26(0.15,0.41)	-57.33(-78.34,-25.41)	1.97(1.55,2.40)
Nepal	2.12(1.07,3.52)	0.04(0.02,0.07)	2.44(1.27,3.82)	0.04(0.02,0.06)	36.48(-26.37,132.02)	0.44(0.10,0.78)
Netherlands	0.36(0.31,0.43)	0.02(0.02,0.02)	0.72(0.60,0.85)	0.04(0.03,0.05)	-44.07(-69.46,-2.82)	2.40(1.37,3.44)
New Zealand	0.10(0.08,0.12)	0.02(0.02,0.02)	0.10(0.08,0.12)	0.01(0.01,0.02)	102.73(20.36,295.01)	-0.78(-1.87,0.32)
Nicaragua	1.30(0.90,1.83)	0.11(0.08,0.16)	0.72(0.50,1.05)	0.05(0.04,0.08)	-42.30(-68.89,2.06)	-1.21(-1.57,-0.86)
Niger	2.46(0.91,4.73)	0.10(0.04,0.20)	6.81(2.74,12.27)	0.09(0.04,0.16)	-64.21(-79.38,-40.75)	-0.43(-0.63,-0.24)
Nigeria	26.08(12.65,38.52)	0.11(0.05,0.17)	69.03(30.37,102.37)	0.11(0.05,0.16)	-51.09(-80.14,-10.21)	-0.41(-0.62,-0.19)
Niue	0.00(0.00,0.00)	0.13(0.07,0.24)	0.00(0.00,0.00)	0.79(0.53,1.18)	-64.78(-82.30,-39.35)	2.60(1.41,3.81)
North Macedonia	0.04(0.03,0.07)	0.01(0.01,0.02)	0.02(0.01,0.03)	0.01(0.01,0.01)	4.22(-49.27,89.05)	-0.86(-1.30,-0.43)
Northern Mariana Islands	0.00(0.00,0.00)	0.03(0.01,0.06)	0.00(0.00,0.01)	0.05(0.03,0.07)	92.26(13.62,224.78)	1.74(-0.36,3.89)
Norway	0.01(0.01,0.01)	0.00(0.00,0.00)	0.01(0.01,0.01)	0.00(0.00,0.00)	198.30(71.18,413.59)	-1.19(-1.73,-0.64)
Oman	0.10(0.05,0.17)	0.02(0.01,0.03)	0.05(0.04,0.08)	0.01(0.00,0.01)	8.58(-43.50,100.79)	-2.17(-2.49,-1.84)
Pakistan	12.79(7.37,20.53)	0.04(0.02,0.07)	37.98(21.72,59.33)	0.07(0.04,0.11)	-7.84(-61.91,211.46)	1.96(1.76,2.16)

location	1990		2021		1990-2021	
	Death cases	Death rate	Death cases	Death rate	Cases change	EAPC
Palau	0.00(0.00,0.00)	0.09(0.05,0.16)	0.00(0.00,0.00)	0.08(0.05,0.13)	-18.71(-41.97,14.83)	0.12(-0.18,0.42)
Palestine	0.14(0.09,0.21)	0.02(0.02,0.04)	0.19(0.13,0.28)	0.02(0.01,0.02)	15.21(-43.98,113.88)	-1.09(-1.40,-0.78)
Panama	0.57(0.47,0.70)	0.10(0.09,0.13)	0.79(0.64,0.94)	0.10(0.08,0.12)	196.99(98.76,372.55)	0.22(-0.07,0.51)
Papua New Guinea	1.25(0.62,2.42)	0.12(0.06,0.23)	4.81(2.42,8.75)	0.20(0.10,0.37)	138.69(29.22,362.53)	2.10(1.84,2.35)
Paraguay	1.90(1.34,2.54)	0.18(0.13,0.24)	1.84(1.30,2.58)	0.14(0.10,0.19)	80.68(3.01,215.72)	-0.58(-0.96,-0.20)
Peru	6.99(4.28,10.00)	0.13(0.08,0.19)	5.14(3.25,7.38)	0.08(0.05,0.12)	62.96(-8.22,180.39)	-1.25(-1.44,-1.06)
Philippines	18.15(9.29,26.44)	0.11(0.06,0.17)	23.57(15.17,30.19)	0.10(0.07,0.13)	88.87(6.74,228.50)	0.40(0.18,0.63)
Poland	1.10(1.05,1.16)	0.02(0.02,0.02)	0.56(0.50,0.60)	0.01(0.01,0.02)	65.52(-27.39,363.14)	-0.26(-1.09,0.57)
Portugal	0.23(0.20,0.27)	0.02(0.01,0.02)	0.19(0.16,0.22)	0.02(0.02,0.02)	29.08(-27.35,124.93)	0.91(0.07,1.75)
Puerto Rico	0.22(0.19,0.26)	0.03(0.03,0.04)	0.07(0.06,0.08)	0.02(0.02,0.02)	29.93(-25.46,136.59)	-1.61(-2.44,-0.77)
Qatar	0.03(0.01,0.04)	0.03(0.02,0.06)	0.05(0.03,0.08)	0.02(0.01,0.03)	-23.22(-57.49,68.27)	-1.81(-2.57,-1.06)
Republic of Korea	5.99(3.01,9.26)	0.07(0.04,0.11)	0.89(0.51,1.42)	0.02(0.01,0.03)	54.22(-14.76,178.02)	-4.84(-5.14,-4.53)
Republic of Moldova	0.20(0.16,0.23)	0.02(0.02,0.03)	0.12(0.10,0.14)	0.03(0.03,0.04)	8.91(-38.11,100.35)	1.81(1.16,2.46)
Romania	0.56(0.44,0.69)	0.01(0.01,0.02)	0.24(0.20,0.29)	0.01(0.01,0.01)	30.49(-29.11,167.95)	-0.34(-0.75,0.06)
Russian Federation	4.80(4.62,5.00)	0.02(0.02,0.02)	5.75(5.37,6.10)	0.03(0.03,0.03)	50.64(1.03,145.62)	1.40(0.70,2.10)
Rwanda	7.22(3.08,12.76)	0.35(0.15,0.62)	3.91(2.35,6.18)	0.12(0.07,0.19)	117.99(20.39,290.21)	-3.84(-4.06,-3.62)
Saint Kitts and Nevis	0.00(0.00,0.01)	0.05(0.04,0.05)	0.00(0.00,0.00)	0.04(0.03,0.05)	49.15(-15.94,173.15)	-0.44(-0.92,0.03)
Saint Lucia	0.03(0.02,0.03)	0.08(0.06,0.10)	0.02(0.02,0.03)	0.11(0.09,0.14)	137.66(80.14,207.33)	0.52(-0.16,1.20)
Saint Vincent and the Grenadines	0.01(0.01,0.01)	0.04(0.03,0.04)	0.01(0.01,0.02)	0.07(0.06,0.09)	104.04(14.58,263.48)	1.53(0.44,2.63)
Samoa	0.06(0.03,0.09)	0.12(0.07,0.20)	0.08(0.05,0.13)	0.15(0.09,0.25)	-45.79(-73.95,10.43)	1.03(0.92,1.15)
San Marino	0.00(0.00,0.00)	0.02(0.01,0.03)	0.00(0.00,0.00)	0.01(0.01,0.01)	-70.33(-84.66,-48.66)	-1.92(-2.07,-1.76)
Sao Tome and Principe	0.03(0.02,0.05)	0.09(0.04,0.14)	0.03(0.01,0.05)	0.05(0.02,0.09)	91.84(8.48,244.86)	-1.39(-1.59,-1.20)
Saudi Arabia	1.53(0.91,2.54)	0.04(0.02,0.06)	0.88(0.54,1.37)	0.02(0.01,0.03)	95.25(9.68,237.43)	-2.18(-2.56,-1.80)

location	1990		2021		1990-2021	
	Death cases	Death rate	Death cases	Death rate	Cases change	EAPC
Senegal	2.29(1.13,3.96)	0.10(0.05,0.18)	3.81(2.11,6.28)	0.09(0.05,0.15)	105.88(13.99,258.20)	-0.15(-0.41,0.12)
Serbia	0.38(0.24,0.55)	0.03(0.02,0.04)	0.06(0.04,0.11)	0.01(0.00,0.01)	72.66(-9.55,225.37)	-4.22(-4.59,-3.85)
Seychelles	0.01(0.00,0.01)	0.04(0.02,0.06)	0.00(0.00,0.00)	0.01(0.01,0.02)	3.27(-41.00,83.30)	-1.27(-2.19,-0.35)
Sierra Leone	1.17(0.49,1.99)	0.11(0.05,0.19)	2.86(1.35,5.15)	0.13(0.06,0.23)	41.52(-22.24,160.78)	0.75(0.55,0.94)
Singapore	0.13(0.11,0.15)	0.03(0.02,0.03)	0.07(0.06,0.08)	0.01(0.01,0.02)	37.39(-26.36,150.06)	-2.96(-4.05,-1.86)
Slovakia	0.19(0.13,0.28)	0.02(0.01,0.03)	0.07(0.05,0.10)	0.01(0.01,0.02)	-7.81(-34.99,23.51)	-0.74(-1.16,-0.32)
Slovenia	0.02(0.02,0.03)	0.01(0.01,0.01)	0.01(0.01,0.01)	0.00(0.00,0.01)	17.23(-31.30,104.21)	-0.95(-1.45,-0.45)
Solomon Islands	0.07(0.03,0.13)	0.07(0.03,0.13)	0.19(0.09,0.32)	0.11(0.06,0.20)	219.39(78.49,457.63)	1.65(1.53,1.77)
Somalia	4.33(1.79,8.10)	0.18(0.08,0.34)	8.30(3.98,14.87)	0.13(0.06,0.24)	190.85(72.17,413.46)	-1.06(-1.37,-0.74)
South Africa	15.71(10.73,20.39)	0.18(0.12,0.23)	14.48(11.49,18.84)	0.14(0.11,0.18)	168.38(66.65,347.19)	-1.92(-2.57,-1.27)
South Sudan	2.40(1.05,4.21)	0.15(0.07,0.26)	4.47(2.10,7.98)	0.16(0.08,0.29)	233.55(95.35,497.56)	-0.15(-0.71,0.41)
Spain	0.77(0.65,0.88)	0.01(0.01,0.02)	1.10(0.95,1.28)	0.02(0.02,0.03)	7.18(-39.32,92.63)	2.42(1.48,3.37)
Sri Lanka	3.95(2.49,5.84)	0.10(0.07,0.16)	2.12(1.36,3.15)	0.06(0.04,0.09)	317.07(155.45,599.34)	-1.56(-2.29,-0.83)
Sudan	6.95(3.87,11.05)	0.13(0.07,0.20)	10.29(6.21,15.95)	0.09(0.06,0.15)	155.59(54.43,314.42)	-0.84(-0.97,-0.70)
Suriname	0.05(0.03,0.06)	0.05(0.03,0.07)	0.05(0.03,0.07)	0.05(0.03,0.07)	166.57(48.80,346.59)	-0.17(-0.48,0.15)
Sweden	0.15(0.12,0.19)	0.02(0.01,0.02)	0.10(0.08,0.12)	0.01(0.01,0.01)	23.62(-34.85,116.91)	-1.64(-2.13,-1.15)
Switzerland	0.25(0.21,0.29)	0.03(0.03,0.04)	0.38(0.33,0.43)	0.04(0.04,0.05)	168.46(55.00,380.36)	1.25(0.51,2.00)
Syrian Arab Republic	1.65(0.93,2.51)	0.04(0.02,0.07)	0.59(0.37,0.84)	0.02(0.01,0.03)	75.40(-4.09,246.16)	-2.78(-3.14,-2.43)
Taiwan (Province of China)	1.14(0.98,1.31)	0.03(0.03,0.03)	1.70(1.45,1.97)	0.08(0.07,0.10)	141.78(32.68,338.29)	5.47(4.42,6.52)
Tajikistan	0.06(0.03,0.12)	0.00(0.00,0.01)	0.10(0.06,0.17)	0.00(0.00,0.01)	196.09(64.87,408.65)	-0.59(-0.97,-0.20)
Thailand	43.32(22.89,66.29)	0.37(0.20,0.57)	12.37(8.39,16.83)	0.18(0.12,0.24)	80.44(5.50,214.20)	-2.50(-2.66,-2.33)
Timor-Leste	0.17(0.10,0.28)	0.09(0.05,0.15)	0.36(0.20,0.63)	0.11(0.06,0.19)	176.95(51.37,395.62)	1.06(0.90,1.22)
Togo	1.01(0.47,1.73)	0.09(0.04,0.16)	1.86(0.88,3.27)	0.09(0.04,0.15)	164.70(79.02,260.67)	-0.21(-0.38,-0.04)

location	1990		2021		1990-2021	
	Death cases	Death rate	Death cases	Death rate	Cases change	EAPC
Tokelau	0.00(0.00,0.00)	0.12(0.06,0.23)	0.00(0.00,0.00)	0.86(0.47,1.43)	-12.19(-66.91,88.39)	2.69(1.22,4.19)
Tonga	0.02(0.01,0.03)	0.07(0.04,0.13)	0.02(0.01,0.04)	0.10(0.06,0.16)	66.36(-4.92,187.82)	1.23(0.93,1.54)
Trinidad and Tobago	0.38(0.30,0.48)	0.14(0.11,0.18)	0.17(0.14,0.22)	0.09(0.07,0.11)	144.76(41.26,330.08)	-1.15(-1.53,-0.78)
Tunisia	1.69(1.07,2.57)	0.08(0.05,0.13)	0.82(0.41,1.42)	0.04(0.02,0.08)	83.34(9.27,217.77)	-1.75(-1.98,-1.53)
Turkey	0.16(0.10,0.28)	0.02(0.01,0.03)	0.23(0.17,0.32)	0.02(0.02,0.03)	121.32(-10.59,541.67)	-0.17(-0.87,0.53)
Turkmenistan	0.00(0.00,0.00)	0.13(0.07,0.21)	0.00(0.00,0.01)	0.19(0.11,0.29)	-40.01(-62.49,-14.67)	1.68(1.50,1.86)
Tuvalu	17.18(10.41,26.91)	0.13(0.08,0.20)	6.05(3.84,9.65)	0.05(0.03,0.07)	-50.84(-73.46,-12.40)	-2.64(-3.01,-2.28)
Uganda	8.11(3.63,14.97)	0.17(0.08,0.31)	16.70(9.28,26.85)	0.13(0.07,0.21)	-8.84(-58.08,114.99)	-1.27(-1.52,-1.03)
Ukraine	1.12(0.69,1.80)	0.01(0.01,0.02)	0.82(0.66,1.01)	0.02(0.01,0.02)	3.94(-72.00,232.79)	0.98(0.70,1.26)
United Arab Emirates	0.28(0.16,0.45)	0.08(0.04,0.13)	0.29(0.16,0.49)	0.03(0.02,0.05)	13.51(-33.91,102.44)	-2.43(-2.90,-1.96)
United Kingdom	0.66(0.64,0.68)	0.01(0.01,0.01)	1.11(0.99,1.21)	0.01(0.01,0.01)	76.06(-3.83,240.19)	2.55(1.48,3.62)
United Republic of Tanzania	13.52(6.87,21.72)	0.19(0.09,0.30)	26.39(14.64,41.82)	0.17(0.09,0.27)	192.70(71.18,514.14)	-0.10(-0.24,0.05)
United States of America	7.55(6.66,8.90)	0.02(0.02,0.02)	6.09(5.65,6.56)	0.01(0.01,0.02)	74.20(-15.61,391.39)	-1.14(-1.32,-0.96)
United States Virgin Islands	0.03(0.02,0.04)	0.12(0.08,0.18)	0.00(0.00,0.00)	0.03(0.01,0.05)	-31.03(-58.28,40.47)	-3.69(-4.14,-3.23)
Uruguay	0.31(0.26,0.36)	0.06(0.05,0.07)	0.15(0.12,0.18)	0.03(0.03,0.04)	-69.25(-76.05,-60.38)	-2.03(-2.58,-1.47)
Uzbekistan	1.63(1.26,2.10)	0.03(0.02,0.04)	1.14(0.90,1.40)	0.02(0.01,0.02)	-41.55(-54.77,-21.60)	-1.28(-1.91,-0.64)
Vanuatu	0.04(0.02,0.08)	0.11(0.05,0.19)	0.12(0.07,0.22)	0.17(0.09,0.29)	-47.08(-69.21,-5.49)	1.65(1.44,1.87)
Venezuela (Bolivarian Republic of)	1.62(1.37,1.92)	0.04(0.03,0.04)	2.49(1.95,3.16)	0.06(0.04,0.07)	411.97(200.08,921.55)	1.50(1.03,1.97)
Viet Nam	6.15(3.60,10.48)	0.04(0.02,0.06)	5.40(2.76,9.64)	0.03(0.02,0.06)	83.84(-3.54,265.90)	0.01(-0.19,0.21)
Yemen	3.72(2.01,6.09)	0.09(0.05,0.14)	7.16(4.22,10.81)	0.08(0.05,0.12)	-90.56(-96.32,-81.37)	-0.33(-0.44,-0.22)
Zambia	3.25(1.34,5.54)	0.14(0.06,0.25)	5.60(2.99,8.63)	0.10(0.06,0.16)	85.92(6.79,209.78)	-1.12(-1.38,-0.85)
Zimbabwe	1.34(0.85,2.08)	0.04(0.03,0.07)	4.28(2.55,6.78)	0.10(0.06,0.17)	48.10(-21.87,170.15)	4.06(3.23,4.90)
