# Supplementary material for: The global burden of pediatric infective endocarditis (5–14 years): epidemiological patterns from 1990 to 2021 and projected trajectories
Source: Front Cardiovasc Med. 2025 Oct 20;12:1657644. doi: 10.3389/fcvm.2025.1657644 (PMC12580122; doi:10.3389/fcvm.2025.1657644)
Supplement: Supplementary file 3 [file Table3.pdf]

Table S3. DALYs of Infective endocarditis in children between 1990 and 2021 at the national level

location	1990		2021		1990-2021	
	DALY cases	DALY rate	DALY cases	DALY rate	Cases change	EAPC
Afghanistan	159.75(89.67,261.69)	5.41(2.76,7.40)	482.19(298.11,733.62)	1.15(0.82,1.79)	201.83(74.18,417.62)	-0.26(-0.55,0.02)
Albania	29.73(17.24,44.68)	5.92(3.45,8.93)	6.39(3.82,9.62)	3.87(1.82,7.67)	-78.51(-86.20,-63.07)	-0.95(-1.95,0.06)
Algeria	542.20(320.58,880.06)	2.45(2.13,2.79)	311.76(205.10,474.41)	7.62(6.49,8.70)	-42.50(-68.14,-4.14)	-2.45(-2.58,-2.33)
American Samoa	0.60(0.26,1.20)	4.76(2.84,7.74)	1.34(0.78,2.18)	3.73(1.99,6.58)	123.97(-7.00,524.71)	4.97(4.13,5.82)
Andorra	0.10(0.06,0.15)	5.81(3.83,8.71)	0.07(0.05,0.10)	5.22(3.28,8.69)	-29.54(-57.84,28.93)	-1.44(-1.60,-1.29)
Angola	204.55(71.10,374.91)	9.05(4.98,15.20)	501.87(241.07,852.32)	8.17(4.08,14.02)	145.35(35.12,365.95)	-1.07(-1.30,-0.84)
Antigua and Barbuda	0.57(0.46,0.69)	9.85(6.62,14.17)	0.54(0.44,0.65)	5.88(4.17,8.04)	-3.98(-26.91,24.00)	-0.28(-1.10,0.54)
Argentina	323.38(278.03,377.67)	4.53(2.33,7.74)	175.08(149.65,205.69)	2.73(1.59,4.38)	-45.86(-57.21,-33.02)	-2.03(-2.26,-1.80)
Armenia	4.96(3.96,6.14)	10.03(5.76,16.76)	1.88(1.63,2.13)	8.10(4.75,13.69)	-62.19(-71.18,-50.16)	-0.14(-0.76,0.48)
Australia	19.68(16.26,23.74)	9.17(4.72,13.31)	36.75(29.16,45.54)	8.34(5.40,10.62)	86.74(47.64,138.33)	1.08(0.41,1.76)
Austria	2.82(2.39,3.25)	8.55(5.42,12.67)	6.07(5.05,7.53)	5.07(3.34,7.33)	115.39(72.96,168.72)	3.91(2.97,4.85)
Azerbaijan	6.57(3.78,10.15)	30.40(16.29,46.25)	5.55(3.46,8.13)	15.25(10.67,20.28)	-15.42(-53.55,48.33)	-1.15(-1.82,-0.47)
Bahamas	5.27(4.43,6.12)	7.02(4.14,11.85)	3.76(2.93,4.74)	8.60(4.82,14.91)	-28.63(-46.32,-4.65)	-2.15(-2.76,-1.53)
Bahrain	4.51(2.99,6.93)	3.00(1.84,5.00)	5.94(4.22,8.44)	2.79(1.52,4.86)	31.68(-13.33,120.62)	-1.06(-1.37,-0.75)
Bangladesh	1073.85(478.47,2101.27)	12.32(7.30,20.62)	1174.75(689.71,1856.38)	19.34(10.80,30.80)	9.40(-43.26,99.78)	0.58(0.40,0.76)
Barbados	4.14(3.29,5.04)	10.61(6.40,16.20)	1.89(1.40,2.42)	15.03(8.84,24.76)	-54.40(-68.55,-34.33)	-0.45(-1.03,0.13)
Belarus	57.10(34.13,95.45)	7.47(3.75,13.90)	25.78(20.83,31.69)	16.23(9.63,26.47)	-54.85(-74.61,-22.76)	-1.30(-1.52,-1.09)
Belgium	16.83(14.61,19.60)	10.30(5.38,18.00)	49.01(40.76,59.33)	14.14(8.57,22.70)	191.24(132.55,260.87)	3.60(2.74,4.47)
Belize	1.49(1.24,1.79)	9.35(4.64,17.99)	1.40(1.15,1.67)	15.79(7.99,28.58)	-5.80(-28.48,19.71)	-1.27(-1.75,-0.78)
Benin	79.71(39.48,135.18)	9.81(5.23,15.75)	235.79(122.97,385.38)	12.25(7.27,19.69)	195.82(79.00,407.42)	0.48(0.39,0.57)
Bermuda	0.18(0.14,0.22)	5.63(2.49,10.37)	0.13(0.09,0.17)	8.98(4.46,15.37)	-29.44(-48.85,-7.08)	0.17(-0.34,0.68)

location	1990		2021		1990-2021	
	DALY cases	DALY rate	DALY cases	DALY rate	Cases change	EAPC
Bhutan	4.09(1.30,7.82)	5.86(3.19,9.99)	3.84(2.09,5.99)	8.14(4.85,12.95)	-6.11(-60.24,199.58)	0.55(-0.01,1.12)
Bolivia (Plurinational State of)	280.57(167.73,437.12)	8.30(3.96,14.60)	225.38(152.37,326.40)	13.18(7.08,23.01)	-19.67(-53.28,43.52)	-1.66(-1.79,-1.53)
Bosnia and Herzegovina	5.04(3.31,7.48)	0.75(0.60,0.93)	1.65(1.22,2.19)	0.46(0.40,0.52)	-67.28(-77.22,-48.43)	-0.93(-1.14,-0.71)
Botswana	48.89(25.78,82.80)	0.43(0.25,0.67)	52.11(31.90,76.31)	0.34(0.21,0.50)	6.58(-37.93,82.08)	-0.21(-0.40,-0.02)
Brazil	3694.71(3308.17,4059.16)	1.37(0.92,1.89)	1986.61(1680.77,2269.54)	0.85(0.71,0.98)	-46.23(-54.61,-38.13)	-0.96(-1.49,-0.44)
Brunei Darussalam	3.12(1.72,5.09)	1.95(1.45,2.86)	1.45(0.89,2.27)	1.06(0.75,1.40)	-53.45(-78.12,-11.61)	-1.83(-2.20,-1.45)
Bulgaria	24.17(20.00,28.77)	1.07(0.86,1.28)	19.58(15.86,23.84)	0.76(0.66,0.90)	-18.98(-38.42,8.55)	1.53(0.87,2.20)
Burkina Faso	169.28(78.59,302.41)	1.86(1.05,2.90)	457.57(244.51,726.23)	1.36(0.94,1.93)	170.30(68.91,340.33)	1.03(0.87,1.19)
Burundi	390.62(175.08,666.77)	0.38(0.21,0.74)	517.94(289.88,848.49)	0.39(0.24,0.64)	32.59(-22.45,138.10)	-1.79(-1.89,-1.69)
Cabo Verde	6.53(3.57,10.95)	1.48(0.96,2.51)	7.39(4.87,11.18)	1.90(1.46,2.71)	13.24(-32.87,90.65)	-0.28(-0.59,0.02)
Cambodia	134.93(80.60,219.46)	2.63(2.04,3.36)	125.77(67.01,221.61)	1.56(1.24,1.88)	-6.79(-58.72,72.36)	-0.90(-1.02,-0.78)
Cameroon	194.48(87.87,344.76)	4.17(2.42,6.26)	656.94(345.14,1038.49)	2.12(1.27,3.19)	237.80(101.66,490.59)	0.60(0.24,0.96)
Canada	56.18(42.87,74.99)	0.68(0.45,1.01)	46.61(38.20,58.11)	0.49(0.36,0.65)	-17.04(-40.45,12.41)	-1.16(-1.31,-1.01)
Central African Republic	61.76(23.25,110.85)	2.02(1.67,2.40)	112.25(47.93,204.66)	2.91(2.35,3.54)	81.75(6.00,213.49)	-0.38(-0.49,-0.27)
Chad	124.24(56.25,217.99)	0.96(0.85,1.09)	519.17(263.20,864.02)	0.98(0.79,1.22)	317.87(157.51,587.39)	1.12(0.94,1.29)
Chile	32.23(26.82,37.64)	1.86(1.57,2.15)	31.44(26.08,37.39)	3.13(2.39,4.13)	-2.44(-23.38,22.54)	0.51(-0.13,1.15)
China	11176.43(5699.36,15286.84)	2.29(2.00,2.56)	2096.00(1484.15,3254.51)	2.31(1.85,2.91)	-81.25(-90.09,-57.78)	-6.02(-6.48,-5.56)
Colombia	671.65(588.58,773.83)	1.09(0.77,1.65)	725.83(600.83,875.02)	0.80(0.55,1.14)	8.07(-13.45,34.58)	0.89(0.33,1.45)
Comoros	25.88(9.29,44.78)	0.84(0.58,1.13)	20.38(12.16,31.18)	0.41(0.28,0.59)	-21.26(-55.39,66.02)	-1.84(-2.36,-1.32)
Congo	38.79(15.66,67.27)	1.59(1.48,1.74)	65.08(33.27,108.94)	1.68(1.42,2.03)	67.76(-2.66,179.70)	-0.55(-0.83,-0.26)
Cook Islands	0.89(0.53,1.49)	1.26(1.01,1.54)	0.46(0.27,0.75)	1.21(0.99,1.45)	-48.06(-70.92,-10.78)	-1.06(-1.34,-0.78)
Costa Rica	48.08(41.06,55.48)	2.15(1.40,3.11)	47.79(41.26,56.84)	0.73(0.49,1.11)	-0.61(-17.94,24.61)	0.44(0.15,0.72)
Croatia	6.56(5.84,7.49)	1.88(1.31,2.73)	4.06(3.26,5.05)	1.44(1.04,1.96)	-38.20(-49.46,-26.07)	0.76(0.32,1.21)

location	1990		2021		1990-2021	
	DALY cases	DALY rate	DALY cases	DALY rate	Cases change	EAPC
Cuba	45.51(36.14,56.50)	0.75(0.63,0.88)	30.66(25.19,37.74)	0.65(0.51,0.83)	-32.62(-48.78,-7.64)	0.08(-0.37,0.53)
Cyprus	3.43(2.33,4.95)	3.57(2.14,5.97)	2.08(1.50,2.87)	2.32(1.87,2.85)	-39.54(-61.24,-9.27)	-1.04(-1.70,-0.38)
Czechia	28.94(24.41,33.42)	4.38(3.69,5.00)	36.18(27.58,47.68)	2.33(1.86,2.94)	25.02(-4.98,57.17)	1.72(1.31,2.13)
C 么 te d'Ivoire	236.46(108.53,396.64)	2.38(1.78,2.94)	610.83(308.77,1005.94)	1.03(0.83,1.28)	158.32(58.91,309.64)	0.92(0.73,1.11)
Democratic People's Republic of Korea	213.76(124.73,322.68)	1.66(1.40,1.92)	126.13(59.46,250.10)	1.96(1.59,2.43)	-40.99(-74.58,16.45)	-1.33(-1.48,-1.17)
Democratic Republic of the Congo	799.30(328.78,1461.58)	2.14(1.77,2.51)	1539.97(770.16,2804.87)	3.11(2.62,3.68)	92.67(11.20,227.97)	-0.27(-0.44,-0.11)
Denmark	4.97(3.99,5.98)	1.81(1.72,1.92)	11.77(9.71,14.51)	3.13(2.81,3.54)	136.71(81.34,211.00)	2.76(2.00,3.52)
Djibouti	17.57(8.52,28.79)	1.30(0.84,2.00)	27.84(16.56,44.10)	1.58(1.31,1.94)	58.50(-9.53,175.08)	-1.55(-1.82,-1.28)
Dominica	1.10(0.71,1.55)	5.56(3.06,9.07)	0.76(0.50,1.12)	2.28(1.40,3.57)	-30.83(-57.79,24.19)	0.22(0.04,0.40)
Dominican Republic	122.40(85.83,168.05)	1.14(1.09,1.20)	143.48(91.27,213.39)	1.41(1.25,1.63)	17.22(-26.70,102.82)	-0.32(-0.58,-0.05)
Ecuador	127.68(108.99,148.87)	6.21(3.21,9.38)	223.86(186.80,267.84)	2.14(1.44,3.07)	75.32(40.35,125.57)	1.18(0.31,2.06)
Egypt	2184.76(1286.39,3491.93)	2.40(2.04,2.84)	1323.72(836.36,2048.34)	1.39(1.17,1.70)	-39.41(-68.68,6.25)	-3.39(-3.50,-3.28)
El Salvador	45.89(31.51,63.77)	0.78(0.64,0.94)	28.13(19.64,39.97)	1.13(0.90,1.40)	-38.71(-61.26,-1.09)	-0.67(-1.06,-0.27)
Equatorial Guinea	9.08(3.42,16.36)	1.64(1.39,1.92)	15.91(5.88,42.10)	1.44(1.18,1.77)	75.15(-20.61,353.02)	-3.07(-3.38,-2.76)
Eritrea	173.63(80.18,295.69)	1.46(0.87,2.24)	193.26(111.80,303.00)	0.91(0.65,1.27)	11.30(-35.74,100.88)	-1.21(-1.30,-1.13)
Estonia	10.02(8.43,11.42)	0.31(0.26,0.36)	3.42(2.73,4.32)	0.70(0.58,0.87)	-65.88(-73.16,-55.86)	-1.36(-1.75,-0.97)
Eswatini	32.82(15.60,54.73)	1.39(1.21,1.62)	38.89(21.47,61.58)	3.71(3.09,4.49)	18.51(-29.59,102.35)	0.40(0.28,0.52)
Ethiopia	2128.89(873.09,3588.87)	2.56(1.74,3.69)	2816.31(1661.02,4647.85)	1.45(1.04,2.00)	32.29(-27.59,168.26)	-1.52(-1.65,-1.39)
Fiji	23.06(13.67,38.60)	0.84(0.67,1.01)	35.10(19.61,55.90)	1.83(1.51,2.26)	52.24(-29.57,239.42)	2.00(1.72,2.28)
Finland	2.18(1.78,2.59)	0.33(0.27,0.40)	4.82(3.96,5.84)	0.80(0.66,0.97)	121.39(71.43,184.22)	3.22(2.32,4.13)
France	165.89(132.52,207.98)	2.12(1.69,2.66)	235.44(183.36,302.71)	2.92(2.27,3.75)	41.92(8.01,88.30)	1.11(0.95,1.28)
Gabon	11.69(5.08,19.24)	0.94(0.76,1.16)	15.69(7.93,27.51)	1.90(1.57,2.31)	34.29(-22.25,123.83)	-0.70(-0.84,-0.56)
Gambia	19.81(9.47,34.09)	1.39(0.95,2.04)	53.68(31.53,85.55)	1.14(0.91,1.40)	170.96(56.13,342.00)	0.46(0.17,0.75)

location	1990		2021		1990-2021	
	DALY cases	DALY rate	DALY cases	DALY rate	Cases change	EAPC
Georgia	12.35(8.26,16.98)	0.65(0.56,0.76)	4.20(3.49,4.81)	1.02(0.85,1.25)	-65.98(-77.86,-47.20)	-1.01(-2.99,1.01)
Germany	79.96(64.44,98.40)	0.49(0.43,0.55)	150.46(124.23,183.37)	0.99(0.79,1.29)	88.18(48.88,143.38)	3.38(2.52,4.25)
Ghana	276.09(146.43,433.37)	0.70(0.59,0.84)	356.51(231.34,525.97)	1.41(1.13,1.74)	29.13(-28.79,115.48)	-1.47(-1.66,-1.29)
Greece	20.39(13.99,30.00)	2.70(2.17,3.51)	11.12(8.84,13.66)	3.25(2.83,3.83)	-45.46(-63.39,-18.54)	-0.22(-0.55,0.11)
Greenland	0.03(0.01,0.08)	1.62(1.38,1.86)	0.03(0.02,0.07)	3.03(2.09,4.38)	0.82(-52.12,118.46)	0.57(0.35,0.79)
Grenada	3.10(2.51,3.80)	1.01(0.76,1.20)	1.27(1.02,1.53)	6.81(5.25,8.73)	-59.02(-69.64,-43.14)	-1.44(-1.80,-1.08)
Guam	1.56(0.78,3.16)	1.78(1.55,2.06)	1.66(0.67,3.46)	4.39(3.62,5.36)	6.44(-69.52,227.74)	3.72(2.56,4.91)
Guatemala	159.93(112.85,225.58)	0.25(0.22,0.28)	191.80(154.54,232.05)	0.21(0.18,0.26)	19.93(-21.74,77.78)	-0.08(-0.32,0.17)
Guinea	127.63(55.52,224.25)	1.31(1.14,1.51)	345.56(168.69,585.10)	2.14(1.81,2.54)	170.75(58.15,374.48)	0.73(0.55,0.91)
Guinea-Bissau	27.87(11.85,48.05)	1.14(0.98,1.30)	49.62(26.09,101.82)	2.35(2.00,2.75)	78.05(-0.33,245.51)	-0.06(-0.34,0.22)
Guyana	9.54(7.64,11.87)	1.58(1.28,1.92)	20.71(15.84,27.04)	1.10(0.88,1.39)	116.95(51.32,201.89)	4.13(2.79,5.48)
Haiti	305.92(171.73,516.09)	2.83(2.38,3.27)	545.33(314.97,859.50)	4.69(3.94,5.67)	78.26(7.46,208.16)	0.49(0.39,0.60)
Honduras	95.08(61.88,147.58)	0.83(0.79,0.87)	85.16(46.45,142.66)	1.47(1.28,1.74)	-10.43(-56.04,62.36)	-1.84(-2.05,-1.64)
Hungary	34.57(30.28,38.76)	4.83(4.15,5.64)	21.56(17.24,27.16)	2.44(2.08,2.86)	-37.65(-50.02,-21.88)	1.30(0.72,1.89)
Iceland	0.28(0.24,0.32)	1.27(1.06,1.49)	0.47(0.39,0.57)	1.23(1.02,1.46)	68.78(35.20,111.09)	2.77(1.48,4.08)
India	7343.06(3922.81,11140.78)	4.89(4.18,5.62)	6188.57(4281.14,7901.73)	2.84(2.29,3.44)	-15.72(-40.43,19.83)	-1.16(-1.45,-0.87)
Indonesia	2636.70(1738.28,3957.81)	1.47(1.12,1.96)	2370.99(1488.32,3944.36)	1.09(0.89,1.36)	-10.08(-41.55,38.96)	-0.29(-0.45,-0.12)
Iran (Islamic Republic of)	917.95(584.09,1180.83)	1.90(1.66,2.25)	407.92(270.48,547.66)	1.53(1.36,1.75)	-55.56(-68.44,-37.40)	-1.55(-2.00,-1.09)
Iraq	246.71(139.98,377.24)	4.68(3.76,5.70)	236.79(154.81,336.90)	4.68(3.82,5.63)	-4.02(-48.73,62.23)	-1.87(-2.25,-1.49)
Ireland	3.38(2.98,3.84)	9.55(8.03,11.10)	6.94(5.52,8.99)	6.26(4.88,7.88)	105.35(59.93,159.94)	3.00(1.98,4.04)
Israel	7.17(5.95,8.51)	9.68(7.69,11.77)	24.07(19.32,29.67)	5.64(4.17,7.22)	235.87(165.49,315.98)	2.96(1.92,4.01)
Italy	175.25(140.83,227.79)	2.85(2.38,3.42)	176.42(153.77,207.95)	1.65(1.36,1.97)	0.67(-25.63,29.42)	0.62(0.24,1.01)
Jamaica	76.87(60.50,96.01)	2.83(2.25,3.51)	31.60(25.46,39.61)	2.49(2.04,3.06)	-58.90(-69.65,-44.56)	-1.97(-2.33,-1.61)

location	1990		2021		1990-2021	
	DALY cases	DALY rate	DALY cases	DALY rate	Cases change	EAPC
Japan	187.62(179.14,197.86)	6.79(4.40,9.60)	152.90(135.37,176.58)	7.44(4.93,10.97)	-18.51(-26.03,-9.16)	-0.39(-1.21,0.43)
Jordan	195.07(131.42,275.37)	7.20(5.05,9.89)	237.06(175.14,314.74)	7.53(4.79,11.20)	21.53(-17.31,83.39)	-2.67(-3.00,-2.34)
Kazakhstan	64.70(48.20,94.64)	14.50(11.74,17.75)	36.95(26.13,48.55)	8.50(6.85,10.21)	-42.89(-66.06,-7.42)	-3.04(-3.71,-2.35)
Kenya	593.21(324.91,849.54)	5.27(4.22,6.55)	936.83(621.31,1264.18)	14.91(11.40,19.47)	57.92(9.45,139.46)	0.18(-0.10,0.46)
Kiribati	1.88(1.14,2.88)	18.51(10.39,31.23)	4.17(2.45,6.86)	19.60(11.32,30.88)	121.17(10.97,302.33)	1.01(0.85,1.17)
Kuwait	15.10(12.29,18.43)	13.82(10.88,17.26)	20.56(16.98,25.19)	7.66(6.17,9.60)	36.11(3.95,86.70)	0.92(-0.41,2.28)
Kyrgyzstan	11.08(8.90,13.25)	6.94(5.52,8.67)	11.18(9.71,13.26)	9.76(7.82,12.30)	0.90(-21.48,40.82)	-0.85(-1.25,-0.45)
Lao People's Democratic Republic	102.42(56.38,172.12)	3.11(2.60,3.66)	119.79(59.89,205.60)	5.98(4.89,7.21)	16.97(-45.40,118.80)	-0.20(-0.33,-0.08)
Latvia	8.73(6.54,10.79)	4.47(2.83,6.25)	2.09(1.68,2.61)	4.21(2.76,6.12)	-76.05(-81.80,-67.87)	-1.00(-1.54,-0.45)
Lebanon	33.90(21.50,51.46)	11.56(9.24,14.45)	29.54(21.00,40.95)	7.81(6.35,9.71)	-12.85(-46.54,41.92)	-1.26(-1.35,-1.17)
Lesotho	49.01(22.54,90.02)	16.74(10.01,26.08)	68.82(31.73,116.36)	9.83(6.65,14.24)	40.44(-21.60,154.88)	1.75(1.52,1.97)
Liberia	55.92(25.12,90.79)	5.07(4.33,5.91)	136.45(65.86,237.58)	6.57(5.48,7.86)	144.02(36.89,332.84)	0.41(0.18,0.65)
Libya	88.91(59.13,124.24)	10.82(6.72,15.30)	82.06(52.85,121.97)	7.16(4.75,10.05)	-7.71(-44.69,58.14)	0.31(0.14,0.49)
Lithuania	9.00(7.60,10.41)	9.01(7.89,10.38)	5.42(4.39,6.70)	10.12(8.38,12.20)	-39.75(-52.44,-22.68)	1.00(0.63,1.36)
Luxembourg	0.70(0.60,0.80)	6.69(5.72,7.72)	2.06(1.43,2.98)	6.74(5.82,8.02)	195.84(106.68,317.26)	2.08(1.48,2.69)
Madagascar	762.22(347.24,1250.88)	3.31(2.27,4.59)	1688.42(1003.60,2634.84)	2.31(1.61,3.28)	121.51(23.34,289.88)	-0.19(-0.35,-0.03)
Malawi	384.37(167.35,642.03)	6.33(4.47,8.93)	585.88(312.31,978.40)	5.68(4.58,6.88)	52.43(-12.18,173.93)	-0.76(-0.93,-0.58)
Malaysia	412.83(277.22,593.98)	6.86(4.46,10.64)	303.25(214.85,414.42)	3.90(2.13,6.54)	-26.54(-55.17,20.24)	-1.41(-1.68,-1.13)
Maldives	2.87(1.47,4.90)	5.37(5.00,5.81)	1.87(1.09,3.00)	4.75(4.25,5.35)	-34.75(-73.14,43.90)	-1.82(-2.20,-1.44)
Mali	157.64(65.51,268.15)	9.40(6.51,13.05)	470.22(246.97,812.92)	4.94(3.53,6.72)	198.29(70.15,408.77)	0.25(0.05,0.45)
Malta	0.60(0.45,0.71)	8.69(7.17,10.52)	2.86(2.21,3.67)	8.73(7.18,10.34)	378.34(266.12,544.16)	5.36(4.37,6.37)
Marshall Islands	1.08(0.54,2.02)	3.00(2.54,3.51)	1.91(1.13,3.12)	4.74(3.73,5.98)	76.45(3.90,288.02)	2.40(2.11,2.69)
Mauritania	35.19(16.93,56.63)	10.41(9.32,11.44)	66.65(37.59,105.48)	6.28(5.31,7.18)	89.39(13.19,215.62)	-0.54(-0.74,-0.34)

location	1990		2021		1990-2021	
	DALY cases	DALY rate	DALY cases	DALY rate	Cases change	EAPC
Mauritius	2.08(1.76,2.43)	15.20(11.07,20.04)	4.92(3.94,5.88)	11.73(8.56,16.03)	136.57(82.91,199.77)	8.74(5.70,11.86)
Mexico	1161.43(1079.87,1255.86)	7.76(4.59,12.59)	1053.57(942.57,1186.08)	3.63(2.39,5.52)	-9.29(-19.97,2.82)	0.82(-0.00,1.66)
Micronesia (Federated States of)	3.12(1.63,5.45)	4.43(2.93,6.81)	2.99(1.81,4.80)	2.93(2.08,4.16)	-4.19(-43.85,98.11)	1.22(1.07,1.37)
Monaco	0.08(0.05,0.12)	16.01(9.42,25.58)	0.10(0.07,0.13)	5.56(3.51,8.60)	24.43(-23.21,102.31)	-1.46(-1.91,-1.01)
Mongolia	10.44(5.88,16.27)	5.53(3.52,7.11)	9.45(6.54,13.46)	2.91(1.93,3.91)	-9.42(-48.32,73.22)	-1.10(-1.68,-0.50)
Montenegro	0.91(0.63,1.23)	4.84(2.75,7.41)	0.31(0.21,0.44)	2.58(1.69,3.67)	-66.10(-78.40,-49.63)	-2.10(-2.65,-1.55)
Morocco	614.98(378.89,957.25)	18.88(12.72,26.65)	277.70(172.30,443.22)	9.35(6.91,12.41)	-54.84(-75.74,-22.37)	-2.30(-2.59,-2.01)
Mozambique	390.72(166.95,665.17)	4.32(3.51,5.27)	808.32(372.44,1385.25)	3.54(2.93,4.34)	106.88(17.41,261.95)	-0.23(-0.43,-0.02)
Myanmar	976.13(560.40,1631.36)	5.17(3.28,7.84)	841.63(493.11,1422.21)	3.39(2.41,4.70)	-13.78(-59.15,65.25)	-0.84(-1.08,-0.60)
Namibia	42.54(18.31,73.67)	7.57(5.04,10.58)	60.11(33.78,98.64)	7.68(4.95,11.41)	41.29(-21.35,148.59)	-0.01(-0.13,0.11)
Nauru	0.30(0.15,0.53)	9.88(6.09,15.38)	0.52(0.31,0.83)	4.25(2.63,6.78)	75.67(-3.13,236.89)	1.97(1.55,2.39)
Nepal	173.44(89.06,284.43)	2.00(1.29,2.95)	205.01(112.18,314.71)	1.30(0.91,1.84)	18.20(-39.94,115.90)	0.50(0.17,0.83)
Netherlands	31.87(27.69,36.85)	1.61(0.90,2.73)	79.87(65.94,97.53)	0.66(0.47,0.97)	150.60(100.20,207.38)	3.24(2.24,4.26)
New Zealand	8.56(7.24,10.02)	2.87(1.76,4.62)	9.67(7.87,11.84)	1.62(1.09,2.30)	12.95(-9.93,42.90)	-0.21(-1.28,0.87)
Nicaragua	108.81(75.39,151.12)	3.07(1.86,5.05)	65.67(46.89,89.39)	1.60(1.05,2.38)	-39.65(-58.89,-8.34)	-1.06(-1.38,-0.73)
Niger	201.30(76.37,382.34)	3.57(2.03,5.42)	566.80(243.24,1010.55)	1.83(1.17,2.57)	181.57(55.30,398.47)	-0.37(-0.56,-0.18)
Nigeria	2130.68(1045.62,3117.92)	6.83(4.38,10.34)	5694.33(2585.52,8354.21)	3.86(2.12,6.44)	167.25(82.82,261.59)	-0.37(-0.58,-0.16)
Niue	0.06(0.03,0.11)	10.46(6.43,16.17)	0.17(0.11,0.25)	4.17(2.78,6.32)	189.37(70.78,494.17)	2.59(1.41,3.79)
North Macedonia	3.90(2.76,5.87)	6.43(3.68,10.26)	1.83(1.25,2.58)	2.88(1.72,4.62)	-53.15(-70.80,-21.45)	-0.57(-0.95,-0.20)
Northern Mariana Islands	0.17(0.08,0.35)	7.05(3.88,11.49)	0.30(0.18,0.45)	6.50(3.93,9.74)	76.40(-13.27,365.36)	2.02(0.29,3.78)
Norway	1.30(1.16,1.46)	6.16(3.46,10.09)	1.37(1.14,1.66)	5.53(3.42,8.41)	5.16(-8.26,21.73)	-0.41(-0.85,0.04)
Oman	8.25(4.63,14.00)	3.58(1.60,7.01)	5.30(3.73,7.78)	3.74(2.20,5.91)	-35.77(-63.73,6.32)	-1.89(-2.18,-1.60)
Pakistan	1051.10(616.60,1677.10)	2.45(0.78,4.69)	3113.43(1826.19,4807.34)	3.04(1.66,4.75)	196.21(99.96,363.96)	1.95(1.76,2.14)

location	1990		2021		1990-2021	
	DALY cases	DALY rate	DALY cases	DALY rate	Cases change	EAPC
Palau	0.22(0.12,0.37)	3.49(1.87,5.30)	0.15(0.09,0.24)	2.43(1.68,3.10)	-29.79(-56.77,40.03)	0.17(-0.12,0.45)
Palestine	11.59(7.44,17.07)	3.38(1.74,5.54)	16.34(11.41,23.02)	3.35(1.83,5.14)	40.92(-22.00,134.44)	-1.01(-1.30,-0.71)
Panama	47.63(39.32,57.71)	3.41(2.00,5.45)	68.27(56.17,80.85)	5.59(3.28,8.63)	43.34(5.13,83.33)	0.27(-0.01,0.54)
Papua New Guinea	98.37(48.81,189.35)	7.40(2.57,13.57)	378.22(191.41,684.80)	5.22(2.51,8.87)	284.47(99.54,639.69)	2.09(1.84,2.34)
Paraguay	160.04(116.55,210.91)	8.59(3.23,15.42)	159.27(116.20,217.60)	7.77(3.32,14.16)	-0.48(-30.85,43.02)	-0.50(-0.85,-0.14)
Peru	581.77(361.45,822.27)	5.89(2.38,10.22)	446.69(296.32,626.83)	5.02(2.57,8.40)	-23.22(-56.37,25.16)	-1.13(-1.31,-0.95)
Philippines	1463.97(753.68,2125.42)	7.68(3.16,14.04)	1900.02(1230.03,2420.36)	6.30(3.15,11.48)	29.79(-3.54,87.87)	0.39(0.17,0.61)
Poland	105.65(98.28,115.76)	7.92(2.98,14.27)	67.28(57.04,81.34)	4.00(1.48,10.59)	-36.31(-43.83,-28.31)	0.26(-0.52,1.05)
Portugal	20.12(17.45,23.19)	4.65(2.02,7.66)	20.08(16.93,23.80)	3.69(1.86,6.46)	-0.22(-16.69,20.30)	1.84(1.05,2.64)
Puerto Rico	20.22(17.25,23.75)	25.27(11.32,43.13)	7.23(5.94,8.84)	14.02(7.85,22.97)	-64.26(-70.76,-55.77)	-0.95(-1.76,-0.14)
Qatar	2.13(1.31,3.43)	19.87(7.13,34.37)	5.01(3.37,7.13)	12.83(7.66,19.63)	135.35(47.43,326.98)	-1.41(-2.10,-0.71)
Republic of Korea	499.95(258.33,755.10)	16.05(7.78,26.30)	96.68(64.99,138.88)	10.41(6.19,16.49)	-80.66(-89.56,-57.29)	-3.94(-4.23,-3.66)
Republic of Moldova	17.26(14.25,20.17)	17.93(8.28,30.53)	11.47(9.65,13.54)	12.03(6.96,18.86)	-33.55(-45.86,-17.10)	2.15(1.58,2.72)
Romania	47.73(38.30,58.50)	14.46(5.93,24.37)	25.04(20.45,30.04)	9.92(5.85,16.37)	-47.54(-60.10,-30.06)	0.21(-0.15,0.56)
Russian Federation	417.90(397.62,442.72)	8.63(4.72,12.35)	578.05(518.71,654.25)	7.37(4.89,9.94)	38.32(26.26,51.42)	1.82(1.22,2.42)
Rwanda	585.03(255.78,1024.50)	23.10(10.52,37.90)	329.66(206.92,507.21)	22.08(13.13,34.46)	-43.65(-71.76,14.11)	-3.69(-3.90,-3.48)
Saint Kitts and Nevis	0.37(0.32,0.43)	14.50(6.31,24.22)	0.22(0.18,0.27)	10.85(5.78,18.12)	-40.53(-53.57,-21.35)	-0.40(-0.87,0.06)
Saint Lucia	2.35(1.87,2.94)	0.93(0.78,1.08)	2.04(1.63,2.57)	3.44(2.75,4.11)	-13.35(-39.21,17.98)	0.58(-0.07,1.23)
Saint Vincent and the Grenadines	0.88(0.74,1.03)	10.30(4.40,17.53)	1.06(0.87,1.28)	8.90(4.10,15.24)	20.64(-5.77,54.90)	1.53(0.47,2.60)
Samoa	4.49(2.39,7.21)	28.61(12.51,50.10)	6.21(3.69,9.98)	10.23(6.42,15.74)	38.25(-20.24,170.45)	1.05(0.93,1.16)
San Marino	0.05(0.03,0.07)	3.20(1.93,5.11)	0.04(0.03,0.06)	1.15(0.66,1.92)	-20.07(-50.27,23.59)	-0.58(-0.73,-0.43)
Sao Tome and Principe	2.57(1.38,4.23)	15.03(6.44,27.93)	2.46(1.20,4.15)	11.09(5.54,19.45)	-4.29(-60.87,97.44)	-1.14(-1.32,-0.95)
Saudi Arabia	126.99(76.74,208.75)	15.28(7.94,24.41)	82.20(53.68,122.21)	14.17(8.26,22.20)	-35.27(-63.22,12.85)	-1.81(-2.15,-1.46)

location	1990		2021		1990-2021	
	DALY cases	DALY rate	DALY cases	DALY rate	Cases change	EAPC
Senegal	189.15(96.08,321.89)	14.14(6.81,25.43)	325.54(188.76,531.40)	11.43(6.74,17.92)	72.11(0.36,189.44)	-0.04(-0.29,0.22)
Serbia	31.93(20.80,46.31)	11.85(5.12,20.03)	6.96(4.67,10.68)	8.89(5.02,13.42)	-78.21(-86.83,-61.73)	-3.53(-3.84,-3.23)
Seychelles	0.50(0.30,0.80)	12.95(6.83,21.94)	0.18(0.10,0.30)	11.26(6.89,16.49)	-64.48(-78.77,-41.12)	-1.09(-1.90,-0.27)
Sierra Leone	95.72(41.34,162.30)	11.23(5.17,20.63)	235.97(117.11,416.37)	16.13(7.43,27.26)	146.52(44.29,326.28)	0.76(0.57,0.95)
Singapore	10.68(9.07,12.65)	11.36(4.89,19.68)	7.32(6.14,8.92)	10.99(6.18,18.03)	-31.42(-45.00,-14.97)	-2.32(-3.34,-1.28)
Slovakia	17.25(11.99,25.00)	15.13(10.48,19.59)	8.20(5.91,11.19)	12.05(9.76,15.50)	-52.45(-69.23,-18.82)	-0.14(-0.49,0.22)
Slovenia	2.18(1.84,2.55)	13.65(6.49,22.77)	1.39(1.10,1.77)	14.29(7.89,22.62)	-35.92(-50.21,-18.25)	0.09(-0.31,0.48)
Solomon Islands	5.40(2.40,9.96)	3.65(2.38,5.61)	14.78(7.34,25.32)	8.52(5.13,13.41)	173.58(49.37,413.91)	1.65(1.53,1.77)
Somalia	353.21(151.28,656.42)	5.55(2.75,9.42)	687.37(343.79,1206.10)	6.31(3.29,10.31)	94.61(13.74,241.53)	-1.01(-1.32,-0.71)
South Africa	1321.69(915.41,1711.65)	5.96(2.76,10.64)	1234.45(999.81,1588.07)	7.30(3.90,11.59)	-6.60(-32.60,23.11)	-1.74(-2.32,-1.17)
South Sudan	198.80(90.11,336.72)	6.76(3.06,11.99)	366.78(178.98,646.01)	7.64(4.01,12.07)	84.50(7.56,201.23)	-0.15(-0.69,0.38)
Spain	65.85(56.45,74.70)	6.67(3.65,11.20)	108.80(92.72,127.57)	7.45(4.92,11.28)	65.22(36.79,100.60)	2.85(1.93,3.77)
Sri Lanka	322.00(204.22,477.12)	7.38(3.34,12.95)	179.34(118.34,259.20)	9.65(4.89,16.06)	-44.30(-68.47,-10.30)	-1.41(-2.09,-0.72)
Sudan	565.37(315.15,902.90)	6.97(3.20,11.69)	847.56(524.92,1299.72)	8.46(4.28,13.94)	49.91(-19.90,170.94)	-0.80(-0.93,-0.67)
Suriname	3.86(2.45,5.40)	7.19(3.43,12.37)	4.16(2.72,6.04)	8.44(4.96,13.45)	7.68(-29.50,89.61)	-0.08(-0.39,0.22)
Sweden	15.53(12.59,18.88)	6.76(3.59,10.62)	13.59(10.84,17.20)	4.33(2.81,6.38)	-12.48(-33.23,13.75)	-0.98(-1.28,-0.68)
Switzerland	21.46(18.04,24.79)	8.02(3.49,14.09)	41.77(35.07,50.45)	9.10(4.44,15.42)	94.61(58.33,143.60)	2.06(1.36,2.77)
Syrian Arab Republic	134.29(76.25,204.01)	9.47(4.03,16.32)	48.70(31.06,68.38)	8.77(4.61,18.01)	-63.74(-78.84,-40.67)	-2.72(-3.07,-2.38)
Taiwan (Province of China)	95.69(83.27,108.94)	8.40(3.77,13.64)	156.59(133.34,178.69)	9.61(4.64,16.74)	63.64(35.56,94.78)	5.68(4.69,6.69)
Tajikistan	5.28(2.92,10.20)	6.57(2.73,11.17)	8.85(5.47,14.41)	6.72(3.53,11.62)	67.71(-30.57,196.58)	-0.35(-0.70,-0.01)
Thailand	3544.59(1899.38,5391.46)	6.36(3.06,10.23)	1058.33(740.47,1407.07)	5.57(3.14,8.82)	-70.14(-81.68,-49.75)	-2.34(-2.50,-2.19)
Timor-Leste	13.52(7.97,22.82)	8.47(3.21,16.09)	28.88(16.20,50.06)	7.39(3.17,13.18)	113.66(-1.47,284.43)	1.04(0.88,1.20)
Togo	83.38(39.27,140.45)	9.17(4.50,13.42)	155.83(77.84,267.94)	8.83(4.01,12.96)	86.89(14.54,220.34)	-0.15(-0.31,0.01)

location	1990		2021		1990-2021	
	DALY cases	DALY rate	DALY cases	DALY rate	Cases change	EAPC
Tokelau	0.04(0.02,0.07)	7.10(3.81,11.69)	0.20(0.11,0.33)	4.65(2.27,7.84)	406.93(198.16,893.40)	2.70(1.24,4.17)
Tonga	1.56(0.85,2.65)	8.65(4.39,14.72)	2.00(1.19,3.19)	7.96(4.62,13.00)	28.79(-29.18,178.40)	1.25(0.95,1.54)
Trinidad and Tobago	31.56(25.23,39.46)	9.21(3.98,15.62)	14.99(12.18,18.64)	10.57(5.24,18.64)	-52.51(-66.47,-31.18)	-1.04(-1.40,-0.69)
Tunisia	139.27(89.35,210.79)	7.75(3.65,13.05)	72.31(39.67,120.76)	7.29(3.64,12.54)	-48.08(-77.34,-8.40)	-1.58(-1.81,-1.35)
Turkey	13.56(8.82,22.95)	5.25(2.26,10.52)	18.68(14.35,26.66)	12.81(7.47,20.85)	37.75(-2.47,101.66)	-0.16(-0.85,0.53)
Turkmenistan	0.20(0.11,0.33)	2.36(1.78,2.94)	0.36(0.21,0.56)	2.16(1.49,2.84)	84.50(-1.84,264.11)	1.69(1.51,1.87)
Tuvalu	1415.64(870.21,2187.78)	20.48(12.08,34.32)	540.37(360.80,819.92)	17.40(10.07,28.28)	-61.83(-79.70,-36.63)	-2.42(-2.76,-2.07)
Uganda	682.61(328.67,1227.69)	0.38(0.15,0.92)	1430.33(844.28,2242.79)	0.44(0.23,0.86)	109.54(19.98,251.97)	-1.18(-1.40,-0.95)
Ukraine	98.76(63.54,152.29)	6.12(3.07,12.39)	75.25(62.05,92.19)	6.99(2.82,14.53)	-23.80(-54.29,27.69)	1.13(0.85,1.40)
United Arab Emirates	23.24(13.30,37.10)	3.35(2.09,4.97)	26.08(15.61,41.85)	2.92(2.07,3.87)	12.21(-42.71,96.39)	-2.22(-2.67,-1.77)
United Kingdom	58.47(55.78,61.67)	11.43(5.74,20.47)	119.77(103.87,141.68)	20.12(12.08,32.32)	104.85(77.85,133.23)	3.22(2.23,4.21)
United Republic of Tanzania	1110.22(576.76,1774.03)	10.46(5.68,19.03)	2205.12(1285.65,3452.94)	61.88(41.67,91.95)	98.62(15.12,237.39)	-0.04(-0.17,0.10)
United States of America	687.95(599.80,813.56)	2.30(1.08,4.73)	623.26(555.76,713.23)	3.73(2.24,5.61)	-9.40(-26.24,5.70)	-0.79(-0.96,-0.61)
United States Virgin Islands	2.17(1.42,3.17)	7.08(3.75,12.16)	0.28(0.16,0.44)	6.62(3.83,10.28)	-87.28(-93.07,-77.37)	-3.02(-3.41,-2.64)
Uruguay	26.67(22.82,30.67)	2.99(2.55,3.51)	13.22(10.66,16.02)	2.13(1.75,2.60)	-50.45(-60.57,-37.13)	-1.86(-2.36,-1.37)
Uzbekistan	136.56(105.63,174.39)	3.90(3.36,4.57)	97.62(77.59,117.79)	3.22(2.64,4.00)	-28.51(-48.94,0.02)	-1.22(-1.85,-0.59)
Vanuatu	3.41(1.63,5.99)	1.76(1.20,2.52)	9.81(5.26,17.12)	1.28(0.91,1.78)	187.94(59.59,486.96)	1.65(1.44,1.86)
Venezuela (Bolivarian Republic of)	136.93(116.12,160.19)	9.60(4.95,17.98)	210.34(165.65,265.40)	67.82(37.20,112.02)	53.61(15.63,104.79)	1.50(1.04,1.96)
Viet Nam	512.74(314.22,856.00)	9.93(5.53,16.76)	464.22(253.33,807.67)	14.73(8.73,23.08)	-9.46(-54.25,46.52)	0.09(-0.10,0.28)
Yemen	304.39(167.54,496.11)	10.37(6.80,15.16)	590.67(357.14,885.22)	2.92(1.69,4.69)	94.05(15.83,222.21)	-0.28(-0.40,-0.17)
Zambia	266.15(114.92,449.69)	12.38(5.61,20.96)	475.12(268.46,717.35)	13.42(6.55,23.64)	78.52(-3.36,225.61)	-1.01(-1.26,-0.76)
Zimbabwe	111.30(72.73,171.30)	10.45(5.82,16.68)	347.84(209.46,547.62)	7.74(4.79,11.87)	212.53(77.00,438.92)	3.92(3.13,4.72)
